# Thick Ascending Limb Specific Inactivation of *Myh9* and *Myh10* Myosin Motors Results in Progressive Kidney Disease and Drives Sex-specific Cellular Adaptation in the Distal Nephron and Collecting Duct

**DOI:** 10.1093/function/zqae048

**Published:** 2024-11-05

**Authors:** Karla L Otterpohl, Brook W Busselman, Jenna L Zimmerman, Malini Mukherjee, Claire Evans, Kelly Graber, Vedant P Thakkar, Jermaine G Johnston, Arooba Ilyas, Michelle L Gumz, Douglas C Eaton, Jeff M Sands, Kameswaran Surendran, Indra Chandrasekar

**Affiliations:** Enabling Technologies Group, Sanford Research, Sioux Falls, SD 57104, USA; Enabling Technologies Group, Sanford Research, Sioux Falls, SD 57104, USA; Basic Biomedical Sciences Graduate Program, University of South Dakota, Vermillion, SD 57069, USA; Enabling Technologies Group, Sanford Research, Sioux Falls, SD 57104, USA; Functional Genomics and Bioinformatics Core, Sanford Research, Sioux Falls, SD 57104, USA; Histology and Imaging Core, Sanford Research, Sioux Falls, SD 57104, USA; Histology and Imaging Core, Sanford Research, Sioux Falls, SD 57104, USA; Enabling Technologies Group, Sanford Research, Sioux Falls, SD 57104, USA; Department of Physiology and Aging, University of Florida, Gainesville, FL 32610, USA; Enabling Technologies Group, Sanford Research, Sioux Falls, SD 57104, USA; Basic Biomedical Sciences Graduate Program, University of South Dakota, Vermillion, SD 57069, USA; Department of Physiology and Aging, University of Florida, Gainesville, FL 32610, USA; Department of Medicine, Renal Division, Emory University, Atlanta, GA 30322, USA; Department of Medicine, Renal Division, Emory University, Atlanta, GA 30322, USA; Pediatrics and Rare Diseases Group, Sanford Research, Sioux Falls, SD 57104, USA; Department of Pediatrics, USD Sanford School of Medicine, Sioux Falls, SD 57103, USA; Enabling Technologies Group, Sanford Research, Sioux Falls, SD 57104, USA; Department of Pediatrics, USD Sanford School of Medicine, Sioux Falls, SD 57103, USA

**Keywords:** actin-associated myosin motors, thick ascending limb, epithelial cargo trafficking, distal nephron adaptation, sodium cotransporters and channels, sexual dimorphism

## Abstract

Our previous work established a role for myosin motor proteins MYH9 and MYH10 in trafficking of thick ascending limb (TAL) cargoes uromodulin and Na^+^-K^+^-2Cl^-^ cotransporter NKCC2. We have generated a TAL-specific *Myh9&10* conditional knockout (*Myh9&10* TAL-cKO) mouse model to determine the cell autonomous roles for MYH9&10 in TAL cargo trafficking and to understand the consequence of TAL dysfunction in adult kidney. *Myh9&10* TAL-cKO mice develop progressive kidney disease with pathological tubular injury confirmed by histological changes, tubular injury markers, upregulated endoplasmic reticulum (ER) stress/unfolded protein response, and higher blood urea nitrogen and serum creatinine. However, male mice survive twice as long as female mice. We have determined this sexual dimorphism in morbidity is due to adaptation of the distal nephron and collecting duct in response to TAL dysfunction and lower NKCC2 expression. We demonstrate that this triggers a compensatory mechanism involving sex-specific cellular adaptation within the distal nephron and collecting duct to boost sodium reabsorption. While both sexes overcompensate by activating epithelial sodium channel (ENaC) expression in medullary collecting ducts resulting in hypernatremia, this is initially subdued in male *Myh9&10* TAL-cKO mice through higher sodium chloride cotransporter (NCC) expression within the distal nephron. Our results indicate that compromised TAL function ultimately results in maladaptation of medullary collecting duct cells which acquire cortical-like properties including ENaC expression. This work further confirms a cell autonomous role for MYH9&10 in maintenance of NKCC2 expression in the TAL and uncover distal nephron and collecting duct adaptive mechanisms which respond to TAL dysfunction.

## Graphical Abstract

**Graphical Abstract:**
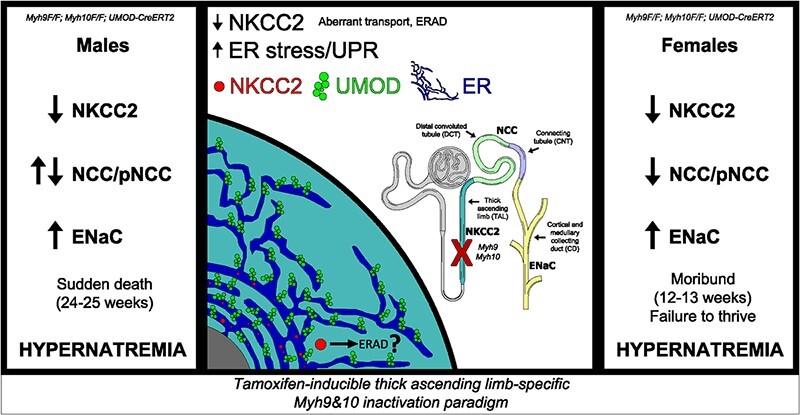

## Introduction

The thick ascending limb (TAL) segment of the kidney utilizes the adenosine triphosphate (ATP)-dependent movement of sodium as well as reabsorption of numerous other ions to facilitate the establishment of the interstitial osmotic gradient along the corticomedullary axis.^1,2^ This gradient is dependent upon the secondary active transport of sodium in the TAL, which is driven by the sodium potassium pump that localizes along the basolateral membrane infoldings.^3^ To meet the metabolic demand required for ion transport, TAL cells have developed an intricate subcellular organelle structure composed of basolateral membrane infoldings that encapsulate long, slender mitochondria.^4–8^ In recent years, the role of actin-associated nonmuscle myosin 2 (NM2) motor proteins, MYH9 and MYH10, in membrane remodeling during subcellular trafficking events has been established.^9–12^ Furthermore, we have shown that MYH9 and MYH10 are uniquely expressed in the renal tubular epithelium and glomerulus, suggesting cell type-specific functional roles for these motor proteins.^13^ In murine kidney, MYH9 expression is limited to podocytes, TAL, and some segments of proximal tubules.^13^ MYH10 is expressed in all renal tubular segments, with little expression in distal tubules and high expression in collecting duct principal cells.^13^

Mutations in the *MYH9* gene are associated with end-stage kidney disease and several studies have explored the underlying pathological mechanism arising from podocytes.^14–23^ We tested the functional role for MYH9&10 proteins in epithelial cell cargo trafficking by generating a renal epithelium-specific conditional knockout (cKO) mouse model of *Myh9&10* (*Myh9&10 Pax8*-cKO).^24^ Loss of these closely related paralogs from the renal epithelium of adult mice resulted in rapidly progressing kidney disease of tubular origin, primarily affecting the trafficking of two TAL proteins, uromodulin (UMOD) and the sodium, potassium, and chloride cotransporter [Na^+^-K^+^-2Cl^-^ cotransporter (NKCC2)].^24^

Here, we aimed to understand the cell autonomous role for MYH9&10 proteins in facilitating a specialized organelle trafficking pathway within TAL cells and to test whether loss of MYH9&10-mediated TAL cargo trafficking is sufficient to drive kidney disease. We generated a tamoxifen-inducible, TAL-specific *Myh9&10* cKO (*Myh9&10* TAL-cKO) mouse model using *UMOD-IRES->CreERT2* transgenic mice.

*Myh9&10* TAL-cKO mice develop progressive kidney disease along with altered expression and localization of UMOD and loss of NKCC2, consistent with TAL cell autonomous functions for MYH9&10. Aberrant trafficking of these cargo proteins also results in upregulation of endoplasmic reticulum (ER) stress/unfolded protein response (UPR) pathway. The hallmark of progressive kidney disease in both male and female mice is histological changes, higher blood urea nitrogen (BUN) and serum creatinine, and higher neutrophil gelatinase-associated lipocalin (NGAL). Interestingly, we also observe a sexually dimorphic phenotype with respect to morbidity in which *Myh9&10* TAL-cKO male mice survive twice as long as female *Myh9&10* TAL-cKO mice (25 weeks compared to 13 weeks, respectively). While characterization of the mouse model indicates a similar pathological course in both sexes, *Myh9&10* TAL-cKO female mice develop hypernatremia earlier than males. Analysis of sodium transporters and channels along the distal nephron and collecting duct of *Myh9&10* TAL-cKO mice reveals adaptation of epithelial cells resulting in higher sodium reabsorption and retention by uncharacteristic epithelial sodium channel (ENaC) expression in the medullary collecting ducts.

Our observations reveal in vivo functional changes within distal nephron and collecting duct segments in response to TAL dysfunction, resulting in progressive kidney disease. Ultimately, our work establishes an essential role for MYH9&10 proteins in cargo trafficking within the TAL epithelium, without which electrolyte imbalances and morbidity occur.

## Materials and Methods

### Experimental Mice

Mice were generated for inducible and tissue-specific knockout of *Myh9* and *Myh10* in the TAL of the loop of Henle using the C57BL/6N-UMOD^em1(cre/ERT2)Amc^/J (*Umod^+/CreERT2^*, Jackson Laboratory strain 030601) transgenic mice and mice containing the *Myh9* and *Myh10* flox alleles.^25–27^ Therefore, the genetic background of the characterized mice was composed of an indeterminate mixture of C57BL/6 and 129 strains. Experimental data was collected from both female and male cKO and control mice at numerous ages. A considerable number of *Umod^+/+^* and *Umod^+/CreERT2^* cohorts were included to characterize the effect of the *CreERT2* allele on kidney structure and function. *Myh9^F/F^; Myh10^F/F^; Umod^+/+^* mice were used as littermate controls for *Myh9^F/F^; Myh10^F/F^; Umod^+/CreERT2^ (Myh9&10* TAL-cKO*)* mice in all experiments unless stated otherwise. All control and *Myh9&10* TAL-cKO animals were provided with food and water ad libitum and housed in the same facility with 12-hour light-dark cycles. Animal weight was monitored for morbidity and animals were humanely euthanized if 20% or greater weight loss was observed. Due to differences in morbidity between female and male *Myh9&10* TAL-cKO mice, all female data is from 12- to 14-week-old mice or younger, whereas male data includes up to 20-24 weeks of age. In these studies, we utilized a minimum of 14 *Umod^+/+^* (control), 16 *Umod^+/CreERT2^* (control), 71 *Myh9&10* (control), and 78 *Myh9&10* TAL-cKO mice. Progressive kidney disease was 100% penetrant in all *Myh9&10* TAL-cKO animals 9 weeks of age and older. Control animals showed no evidence of renal disease. Penetrance at younger ages (6 and 7.5 weeks) was not determined, as disease pathophysiology is less evident in these early stages.

### Tamoxifen Preparation and Injections

Genetic recombination of the floxed alleles was induced by tamoxifen administration. Tamoxifen was prepared fresh the day of injection by dissolving powdered tamoxifen (T5648, Sigma) in a freshly prepared solution of 90% corn oil (C8267, Sigma) and 10% 200-proof ethanol at a final concentration of 30 mg/mL tamoxifen. The solution was protected from light and fully dissolved by mixing at room temperature on an end-over-end tube rotator for 3-4 hours until ready for use. Mice received 3 injections spaced every 72 hours between 5 and 6 weeks of age. Each injection consisted of 100 µL of injection solution (3 mg tamoxifen) given intraperitoneally. *Myh9&10* TAL-cKO, *Umod^+/CreERT2^*, and littermate control (*Myh9*^f/f^; *Myh10*^f/f^; *Umod*^+/+^) mice received tamoxifen unless stated otherwise. Injections were performed on alternating sides of the abdomen to minimize injury. Mice were not subjected to any experimental procedures until 72 hours after the final injection to allow time for tamoxifen elimination from the body.

### Urine Collection

Urine was collected by singly housing mice in metabolic cages with acidified water containing 1% sucrose during the 24-hour period. No food was provided during urine collection. Twenty-four-hour collections were done at 6, 7.5, 9, and 12 weeks of age in female mice and 6, 7.5, 9, 12, 16, and 20 weeks of age in male mice. Mice treated with vehicle or amiloride were provided only with acidified water (no sucrose) during 16-hour urine collection.

### Serum and Tissue Collection

Serum and kidneys were collected from female mice at 6, 7.5, 9, and 12-14 weeks of age and from male mice at 6, 7.5, 9, 12, 16, and 20 weeks of age. Mice were anesthetized with isoflurane and a cardiac puncture was performed to obtain blood for serum analysis. Kidneys were harvested post-exsanguination and were either surface fixed in 4% paraformaldehyde (PFA) or Bouin’s fixative for imaging experiments or flash frozen in liquid nitrogen for immunoblot and messenger RNA (mRNA) analysis.

### Urine and Serum Analysis

Urine pH was determined for fresh samples utilizing a Mettler Toledo micro-pH electrode (01-913-998, Fisher). The ADVIA 1800 chemistry system (Siemens) was used to analyze frozen urine samples for urinary sodium (electrode), potassium (electrode), calcium (10341116, Siemens Healthcare Diagnostics), glucose (10335891, Siemens Healthcare Diagnostics), and total urinary protein (11319151, Siemens Healthcare Diagnostics). The same machine was used to analyze frozen serum samples for sodium (electrode), potassium (electrode), glucose (10335891, Siemens Healthcare Diagnostics), calcium (10341116, Siemens Healthcare Diagnostics), urea nitrogen (10309051, Siemens Healthcare Diagnostics), creatinine (10309050, Siemens Healthcare Diagnostics), and albumin (10311832, Siemens Healthcare Diagnostics). Urine samples were excluded from analysis if total urine produced was less than 100 µL or greater than 8 mL during the collection period. Serum samples with potassium concentration in excess of 5 mmol were excluded from all analyses (4 samples) due to suspected hemolysis and were replaced with samples from additional cohorts. We also excluded samples if volume was insufficient to measure all analytes of interest.

### Amiloride Treatment

Twelve-week-old tamoxifen-treated female and male control (*Myh9*^f/f^; *Myh10*^f/f^; *Umod*^+/+^) and *Myh9&10* TAL-cKO mice were assigned by cage to either vehicle or amiloride (2 mg/kg) treatment groups. Mice received an intraperitoneal injection of 250 µL of vehicle or 250 µL vehicle with amiloride. Animals were immediately placed into metabolic cages with acidified drinking water for 16 hours for urine collection. Control and *Myh9&10* TAL-cKO male mice produced sufficient urine for analysis regardless of treatment. Urine production by female control animals was more limited and many did not produce sufficient volumes of urine for analysis regardless of treatment. Urine production was not significantly different between treatment groups for any given genotype.

### Determination of NGAL Levels

The mouse Lipocalin-2/NGAL ELISA kit (MLCN20, R&D Systems) was used to measure urinary NGAL concentrations. Urine samples were diluted 40-fold and the assay was performed following the protocol provided with the kit. Standards were plotted linearly (log of lipocalin-2 and log of optical density) as recommended in the kit manual. A total of 6 controls and 6 KOs were analyzed for each sex at each timepoint assayed.

### Determination of Copeptin Levels

The mouse Copeptin ELISA kit (LS-F7101, LS Bio) was used to measure serum copeptin concentrations. Serum samples were diluted 10-fold and the assay was performed following the provided protocol. Standards were plotted linearly (log of antigen and log of optical density) as recommended in the kit manual, including the zero standard.

### Histology, Fluorescent Immunohistochemistry, and Image Analysis

Tissues stained for hematoxylin and eosin were fixed in 4% PFA and embedded in paraffin prior to staining. Tissues stained for periodic acid-Schiff (PAS) and Masson’s trichrome were fixed in Bouin’s fixative and embedded in paraffin prior to staining. All histology slides were imaged on a Nikon NiE light microscope or on an Aperio Versa slide scanner.

Immunofluorescence staining was performed following the protocol from Otterpohl *et al*.^13^ Membrane-associated proteins and structures were visualized in sections fixed with Bouin’s fixative, whereas intracellular proteins were visualized on sections fixed with PFA. Table S1 lists the primary antibodies used in this study. We also designed a custom antibody targeted toward mouse MYH9 antigen. Sino Biological developed the polyclonal IgG custom antibody against mouse MYH9 protein using the amino acid sequence CSDEEVDGKADGADAKAAE. This sequence is in the C-terminus of murine MYH9 and is not present in the murine MYH10 protein. BLAST (NCBI) alignment of the chosen peptide sequence did not predict cross-reactivity with any other endogenous mouse proteins.

Secondary antibodies (diluted 1:250) were conjugated to Cy3, Alexa-Fluor488, Cy5, or Alexa-Fluor647 and were procured from Jackson ImmunoResearch or Life Technologies. Nuclei were stained with Hoechst 33342 (62249, Thermo Fisher Scientific). Coverslips were mounted with the fluorescence mounting media, ProLong Gold (P36930, Thermo Fisher Scientific).

Immunostained slides were imaged using a Nikon A1 confocal or a Nikon CSU-W1 SoRa super-resolution microscope and processed using ImageJ/Fiji software. Maximum intensity projections were generated in ImageJ software by merging single-frame images of z-stacks exported from Nikon software (NIS Elements).

### Quantification of CD3^+^ T-cells

CD3^+^ cells were quantified following the protocol used in Otterpohl *et al*.^24^ Briefly, PFA-fixed kidney sections were stained for CD3 and wheat germ agglutinin (WGA) and sections imaged using the 40× objective on a Nikon A1R microscope (images are 317.4 × 317.4 µm). For all kidneys evaluated, 15-20 images (fields) were obtained across both the medullary and cortical regions of the section. For the 9-week-old samples, 3 control (60 fields) and 3 *Myh9&10* TAL-cKO (60 fields) female kidneys and 3 control (60 fields) and 3 *Myh9&10* TAL-cKO (56 fields) male kidneys were analyzed. For the analysis of 12-week-old kidneys, 4 control (70 fields) and 5 *Myh9&10* TAL-cKO (92 fields) female kidneys and 3 control (59 fields) and 4 *Myh9&10* TAL-cKO (80 fields) male kidneys were analyzed for CD3^+^ cells. For the analysis of 20-week-old male kidneys, 4 control (71 fields) and 4 *Myh9&10* TAL-cKO (70 fields) kidneys were analyzed. Counts were performed manually using the ImageJ software.

### Protein Lysate and Immunoblotting

Whole kidney lysates were prepared following a previously published protocol.^28^ Total protein concentration was quantified using the Pierce BCA Assay Kit (Pierce, Thermo Fisher Scientific). Lysates containing 20 µg of total protein were mixed 1:1 with 2× Laemmli (Bio-Rad) containing 2-mercaptoethanol (Bio-Rad), heated at 95°C for 5 minutes, and loaded on 10- or 15-well precast 10% Mini-PROTEAN TGX polyacrylamide gels (Bio-Rad). When blotting for epithelial sodium channel gamma subunit (γENaC), samples were heated for 3 minutes at 95°C prior to loading and were loaded on 10-well 4%-20% Mini-PROTEAN TGX polyacrylamide gels. To better assess the cleavage of γENaC,^29^ 20 µg of protein was treated with 0.5 µL of PNGaseF (New England Biolabs) following the manufacturer’s protocol. PNGaseF-treated samples were then mixed 1:1 with 2× Laemmli (Bio-Rad) containing 2-mercaptoethanol (Bio-Rad), heated at 95°C for 3 minutes, and 28 µL of each sample was loaded on 10-well 4%-20% Mini-PROTEAN TGX polyacrylamide gels.

Urine samples were concentrated using Pierce Protein Concentrators (Fisher, 88512) according to manufacturer instructions and total protein was quantified using the Pierce BCA Assay Kit. Twenty micrograms of urinary protein was mixed 1:1 with Laemmli buffer and loaded on a 15-well 10% Mini-PROTEAN TGX polyacrylamide gel (Bio-Rad). Table S1 contains information pertaining to primary antibodies. All secondary antibodies were conjugated to HRP and were used at a dilution ranging between 1:4000 and 1:8000 (Jackson Immuno Research). Tubulin, GAPDH, and sometimes Ponceau stain were utilized to assess variability in loading. If loading inconsistencies were observed, samples were quantified by BCA assay again and the blot(s) repeated.

#### Quantification

Immunoblots were imaged using the LI-COR Odyssey system. Tiff images were exported and converted to 32 bit in ImageJ for analysis. The gel analysis tool in ImageJ was used to determine the relative density of the bands. To normalize immunoblot results, the control (see figure legends for respective control genotypes) intensity values determined by the ImageJ gel analysis tool were averaged, and the intensity of each band was divided by the control mean value. This process was repeated for individual blots so that each was normalized to its respective control mean value. Where multiple blots were statistically analyzed together, normalization was performed first in Excel, followed by GraphPad Prism 10 statistical analysis.

### Gene Expression

Whole mouse kidneys were homogenized in 4 mL RLT buffer (Qiagen) using an Ultra Turrax T25 homogenizer for 45 seconds. The resulting homogenate was passed 10 times through a 20-gauge needle (309 575 BD) and then centrifuged at 4000 × *g* for 20 minutes at room temperature. A portion of the lysate was used for ribonucleic acid (RNA) extraction using the Qiagen RNeasy Mini Kit following the manufacturer’s instructions. On-column DNAse digestion was performed to eliminate genomic DNA. The extracted RNA was quantified using a nanodrop spectrophotometer. Three to five micrograms of total RNA was reverse transcribed using up to 5 gene-specific primers with GoScript Reverse Transcription Kit (Promega) in a 20 µL volume. The reverse transcription reaction was diluted to 65 and 4 µL of the template was used per well for quantitative Polymerase Chain Reaction (PCR) using iTaq Universal SYBR Green Supermix (Bio-Rad) in a 20 µL reaction. Each sample was run in triplicate in a Bio-Rad CFX96 instrument. Change in gene expression is represented after normalization to housekeeping gene (GAPDH). Primer sequences are provided in Table S2.

### Cell Culture Experiments

Mouse inner medullary collecting duct cells (IMCD3) were seeded into 23-mm permeable Falcon cell culture inserts (Corning, Tewksbury, MA, USA) and maintained in a DMEM/Ham’s F-12 50/50 mix culture media with L-glutamine and HEPES (Genesee Scientific, El Cajon, CA, USA) at pH 7.4 containing 5% FBS (GIBCO) and 1% Pen Strep (Penicillin Streptomycin; GIBCO, Grand Island, NY, USA) at 37°C with 5% CO_2_. Equal amounts of 1 m urea and 1 m NaCl solutions were added to the cell culture media to increase the osmolality from the 300 mmol/kg baseline following previously published protocol,^30^ which was confirmed using a VAPRO Vapor Pressure Osmometer (ELITechGroup, Logan, UT, USA). Inner medullary collecting duct cells were grown on inserts in 6-well plates containing media at 300, 450, or 600 mmol/kg osmolality for 4-5 days to achieve >75% confluence. After the 4-5 day growth period, RNA was isolated from cells. Only cells with a passage number of <40 were used for experiments.

#### RNA Isolation and qPCR

Total RNA was isolated from IMCD3 cells using TRIzol (Invitrogen) according to the manufacturer’s instructions. RNA was treated with DNA-free DNAse I (Ambion). DNAse I-treated RNA samples were used as a template for reverse transcription with the High Capacity cDNA Reverse Transcription Kit (Applied Biosystems). TaqMan probes for *Scnn1a* (Mm00803386_m1, Applied Biosystems), *Scnn1b* (Mm00441215_m1, Applied Biosystems), and *Scnn1g* (Mm00441228_m1, Applied Biosystems) were used for gene expression analysis. Cycle threshold (Ct) values were normalized using the housekeeping gene β-actin (Mm02619850_g1, Applied Biosystems), and relative expression was quantified using the ΔΔCt method.^31^ Gene expression levels were made relative to IMCD3 cells grown in 300 mmol/kg osmolality media.

### Statistics

Statistical analyses were conducted in GraphPad Prism 10. Descriptive statistics such as group mean, standard deviation, and standard error were generated, and the normality of data was checked using Shapiro-Wilk and Kolmogorov-Smirnov tests. Serum, urine, and most immunoblot data was analyzed using unpaired *t*-test with Welch’s correction to account for unequal standard deviation or Mann-Whitney U test, depending on the parametricity of the sample groups. One-way ANOVA was used for immunoblots and transcript analyses that compared more than 2 groups, followed by post-hoc Dunnett’s T3 multiple comparisons test. Kruskal-Wallis with post-hoc Dunn’s test was used when data were not normally distributed. Two-way ANOVA with Tukey’s multiple comparison test was used for gene expression and amiloride treatment analysis and in immunoblot analysis, in which more than 2 variables were compared.

### Study Approval

All experiments involving mice were approved by the Sanford Research Institutional Animal Care and Use Committee (IACUC).

## Results

### *Umod-creERT2* Transgene Efficiently Inactivates *Myh9* and *Myh10* in the TAL

We crossed *Myh9^f/f^* and *Myh10^f/f^* mice with *Umod^+/CreERT2^* mice (C57BL/6N-UMOD^em1(cre/ERT2)Amc^/J (*Umod^+/CreERT2^*, JAX strain 030 601) to conditionally inactivate the *Myh9* and *Myh10* genes in the TAL tubules. Mice harboring *Umod^+/CreERT2^* allele were generated as a part of the GUDMAP consortium and results from the preliminary characterization can be found online (https://www.atlas-d2k.org/gudmap/Docs/Mouse_Strains/UmodIRESCE_strain_report_051017.FNa_APM.J.pdf). In our experiments, we included considerable numbers of age-matched *Umod^+/+^* and *Umod^+/CreERT2^* genetic control cohorts to characterize the effect of the *CreERT2* allele on kidney structure and function, and we present data from these cohorts throughout the results section. *Myh9^F/F^; Myh10^F/F^; Umod^+/+^* mice were used as littermate controls for *Myh9^F/F^; Myh10^F/F^; Umod^+/CreERT2^ (Myh9&10* TAL-cKO*)* mice.

**Figure 1.**
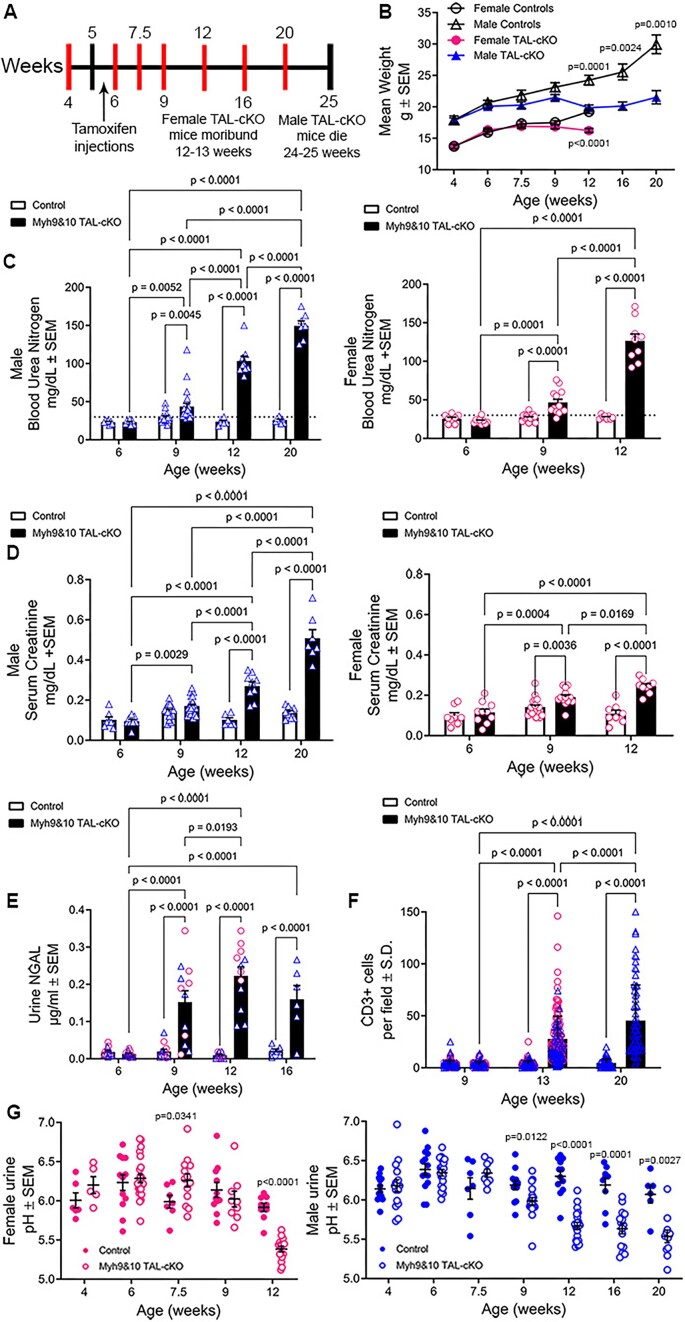
*Myh9&10* TAL-cKO mice develop progressive kidney disease and show sexual dimorphism in morbidity. (A) Timeline depicting experimental protocol for *Myh9&10* TAL-cKO (TAL-cKO) characterization. (B) *Myh9&10* TAL-cKO (TAL-cKO) female mice fail to gain weight compared to littermate controls (*Myh9*^f/f^; *Myh10*^f/f^; *Umod*^+/+^). At 12 weeks of age TAL-cKO females are significantly smaller than littermate controls and meet euthanasia criteria by 14 weeks of age. TAL-cKO male mice also fail to gain weight and are smaller than littermate controls starting at 12 weeks of age and remain significantly smaller at 16 and 20 weeks of age. Male TAL-cKO mice spontaneously die around 24-25 weeks of age. (C) Blood urea nitrogen (BUN) levels become progressively higher during disease progression in female and male TAL-cKO mice compared to littermate controls. Female and male TAL-cKO mice exhibit higher BUN starting at 9 weeks of age. The dotted line indicates normal BUN value (30mg/dL). (D) Serum creatinine levels in both female and male TAL-cKO mice become progressively higher compared to littermate controls. (E) Quantification of the tubular injury marker NGAL in the urine shows elevated concentration in both male (triangles) and female (circles) TAL-cKO mice as early as 9 weeks of age compared to littermate controls, and NGAL was further elevated at later time points in both female and male mice (n=6 each sex). (F) Quantification of CD3+ cell counts show immune cell infiltration at 13 weeks of age in female (circles) and male (triangles) and 20-week-old male TAL-cKO mice compared to littermate controls. (G) 24-hour urine samples collected from female and male TAL-cKO mice indicate higher urine acidity compared to littermate controls. Body weight was analyzed independently for each sex by age using an unpaired t test with Welch’s correction or Mann-Whitney test and graphed together. BUN and creatinine were analyzed independently for each sex by two-way ANOVA. Male and female urine NGAL data was combined and analyzed by two-way ANOVA with post-hoc Tukey’s test. Male and female CD3 data was combined and analyzed by two-way ANOVA with post-hoc Tukey’s test. Sample numbers for CD3+ counts can be found in materials and methods. Mean + SEM values are graphed with individual samples; significant p values are also listed on graphs. Sample numbers for serum and urine data at each timepoint can be found in the supplemental tables. Urine pH was analyzed independently for each sex and age using an unpaired t test with Welch’s correction or Mann-Whitney test.

*Myh9&10* gene inactivation within TAL cells was induced using 3 consecutive tamoxifen injections between 5 and 6 weeks of age (Figure 1A). Samples were collected from cohorts of *Myh9&10* TAL-cKO and littermate controls at varying time points (Figure 1A). We confirmed the loss of MYH9 and MYH10 proteins within the TAL segment using immunostaining of *Myh9&10* TAL-cKO kidney sections and littermate controls (Figure S1A-F). Reporter analysis from the preliminary characterization of the transgenic mice has shown cre recombinase expression within the distal nephron (DCT1) segment (link above). Therefore, we carefully analyzed the transitional tubular region between TAL and DCT1. Based on our previous work, MYH9 is not expressed in the distal convoluted tubule (DCT) and MYH10 is expressed only at low levels on the apical membrane of DCT cells.^13^ Thick ascending limb tubules with NKCC2-positive staining, which abruptly transitioned into sodium chloride cotransporter (NCC)-positive DCT1 tubules, show loss of MYH10 protein in the TAL region but maintain intact MYH10 expression in the DCT1 segment (Figure S1G-J). We also performed coimmunostaining to visualize the parvalbumin- and NCC-positive DCT1 segment and our results show that DCT1 cells maintain MYH10 expression (Figure S1K-R). We also confirmed that MYH9 and MYH10 protein expression is intact in untreated (no tamoxifen) *Myh9&10* TAL-cKO mouse kidneys, indicating that loss of protein happens only with tamoxifen induction (Figure S2).

### Thick Ascending Limb Specific *Myh9* and *Myh10* cKO in Mice Results in Progressive Kidney Disease

The experimental paradigm to characterize *Myh9^f^*^/f^; *Myh10^f^*^/f^; *Umod^+/CreERT2^* (*Myh9&10* TAL-cKO) and control littermates is described in Figure 1A. We observe sexual dimorphism in life span (time of morbidity) in *Myh9&10* TAL-cKO mice compared to littermate controls. Female *Myh9&10* TAL-cKO mice experience a >20% reduction in body weight by 12-13 weeks of age indicating a failure to thrive phenotype and necessitating euthanization (Figure 1A and B). When compared to littermate controls, *Myh9&10* TAL-cKO male mice have a significantly lower body weight starting at 12 weeks of age and a reduced lifespan of 24-25 weeks of age (Figure 1A and B). Due to the spontaneity in time of death in male mice (24-25 weeks), we selected 20 weeks of age as the end point for phenotypic characterization of male *Myh9&10* TAL-cKO mice and their littermate controls.

**Figure 2.**
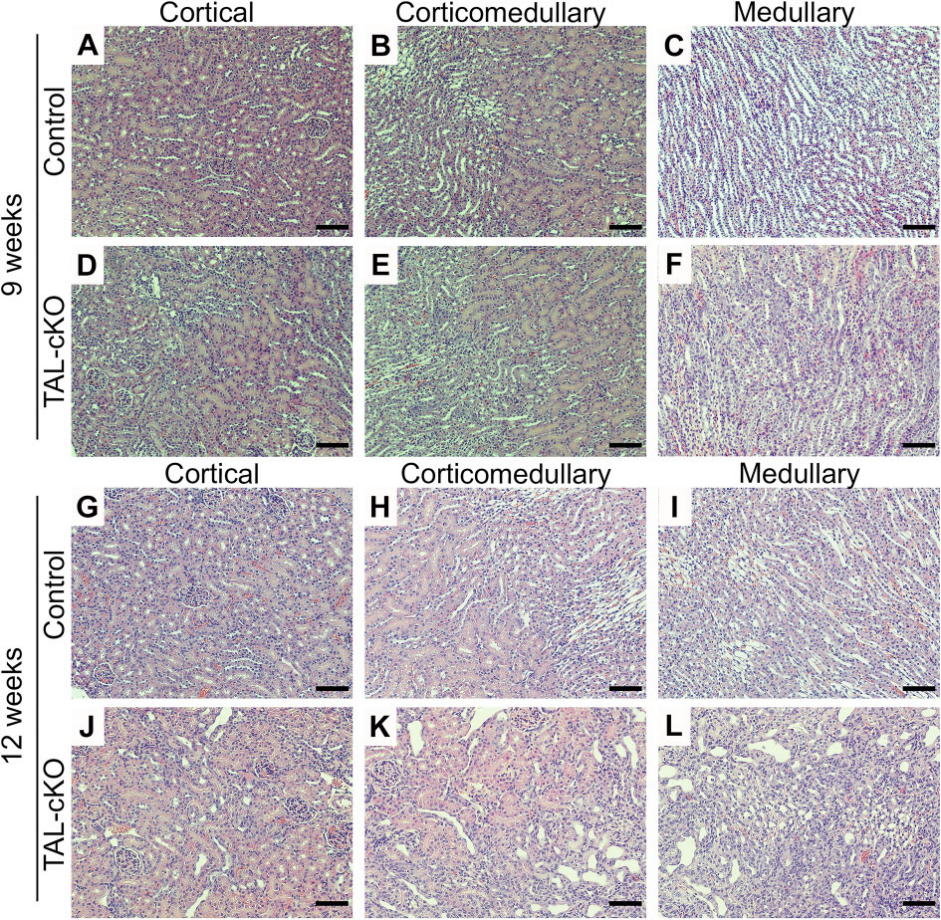
Hematoxylin and eosin (H&E) staining of female Myh9&10 TAL-cKO kidneys shows progressive histological changes. (A-C) Representative images from 9-week-old littermate control (*Myh9*^f/f^; *Myh10*^f/f^; *Umod*^+/+^) kidneys show healthy renal tissue in the cortical, corticomedullary, and medullary regions. (D-F) 9-week-old *Myh9&10* TAL-cKO (TAL-cKO) mice have minimal tubular dilation and cellular density. (G-I) Representative images of 12-week-old controls for the cortical, corticomedullary, and medullary regions also show healthy renal tissue. (J-L) Evaluation of 12-week-old TAL-cKO female kidneys shows focal areas of tubular dilation and extensive damage with infiltrating cells in the corticomedullary and medullary regions Scale bars = 100µm. (n= 3-4 kidneys each).

Our experiments show that conditional genetic inactivation of *Myh9* and *Myh10* in the TAL segment results in progressive kidney disease. Serum chemistry reveals higher BUN levels in male and female *Myh9&10* TAL-cKO mice starting at 9 weeks compared to littermate controls (Figure 1C and Tables S6 and S7), and levels become progressively higher, reaching 126.6 ± 9.1 mg/dL (mean ± SEM) at 12 weeks in female *Myh9&10* TAL-cKO mice and 149.4 ± 6.7 mg/dL (mean ± SEM) at 20 weeks in male *Myh9&10* TAL-cKO mice, whereas age-matched control littermates (*Myh9^f^*^/f^; *Myh10^f^*^/f^) maintain normal BUN levels between 22 and 32 mg/dL (Figure 1C, Tables S6 and S7). Mean serum creatinine progressively becomes higher in both female and male *Myh9&10* TAL-cKO mice starting at 9 and 12 weeks of age, respectively. Control mice maintain normal serum creatinine values throughout all time points (Figure 1D, Tables S6 and S7). Histological examination of hematoxylin and eosin stained kidneys reveals progressive structural abnormalities that become evident at 9 weeks of age, with minor changes in tubular dilation and cellular infiltration that are more severe by 12 weeks of age in both female and male *Myh9&10* TAL-cKO mice compared to controls (Figure 2, Figures S3 and S4). Tubular injury originates in the medullary region and progresses into the corticomedullary and cortical regions of the kidney in both female and male *Myh9&10* TAL-cKO mice (Figure 2, Figures S3 and S4). Masson’s trichrome staining at 13 weeks of age in male and female *Myh9&10* TAL-cKO mice reveals interstitial cellular infiltration as well as fibrosis, as evidenced by light blue staining of the interstitium (Figure S5). In males, the progressive tubular dilation, cellular infiltration, and fibrosis were present also at 20 weeks of age (Figure S5).

Consistent with the development of progressive kidney disease in *Myh9&10* TAL-cKO mice, we observe higher urinary NGAL starting at 9 weeks of age (Figure 1E) and higher CD3-positive T-cell infiltration by 13 weeks of age in male and female mice (Figures 1F and S6). Additionally, the urine pH is significantly lower starting at 9 weeks of age in *Myh9&10* TAL-cKO males and 12 weeks of age in *Myh9&10* TAL-cKO females (Figure 1G, Tables S8 and S9).

Immunoblot analysis of urine samples from 13-week-old *Myh9&10* TAL-cKO mice and littermate controls was done to detect kidney injury molecule 1 (KIM1), a proximal tubule injury marker. Unlike the pan renal tubular *Myh9&10* cKO mice (*Myh9&10 Pax8*-cKO), which showed extensive damage to brush borders in the proximal tubules and had high levels of urinary KIM1,^24^ 13-week-old female and male *Myh9&10* TAL-cKO mice exhibit relatively low levels of urinary KIM1 protein (Figure S7). Periodic acid-Schiff staining also confirms tubular dilation and cellular infiltration at 13 weeks of age in both male and female *Myh9&10* TAL-cKO kidneys and shows lower staining intensity of proximal tubule brush border, though there is no loss of brush border (Figure S8). However, proximal tubular function may be compromised in female *Myh9&10* TAL-cKO mice at 9 weeks of age, as evidenced by significantly higher urinary excretion of protein and glucose (Table S8). Overall, these observations are consistent with the development of progressive kidney disease of tubular origin without severe proximal tubular injury.

We also characterized cre-only control mice (*Umod*^+/CreERT2^) along with control littermates (*Umod^+/+^*) treated with tamoxifen. H&E staining (Figures S9A and S9B) reveals normal renal histology in *Umod^+/CreERT2^* and *Umod^+/+^* mice, while serum chemistries reveal normal levels of BUN and creatinine in *Umod^+/CreERT2^* mice, together confirming normal kidney function (Figures S9E and S9F). Other metabolic parameters analyzed by urine and serum chemistry also show no differences between *Umod^+/+^* and *Umod^+/CreERT2^* kidney function (Tables S3-S5). Thus, the presence of floxed *Myh9&10* alleles and the *Umod*^+/CreERT2^ transgene together are required for development of the observed kidney disease.

### Uromodulin Expression and Localization in *Myh9&10* TAL-cKO Mice Are Influenced by Both *Umod*^+/CreERT2^ Transgene and Loss of MYH9&10 Proteins

**Figure 3.**
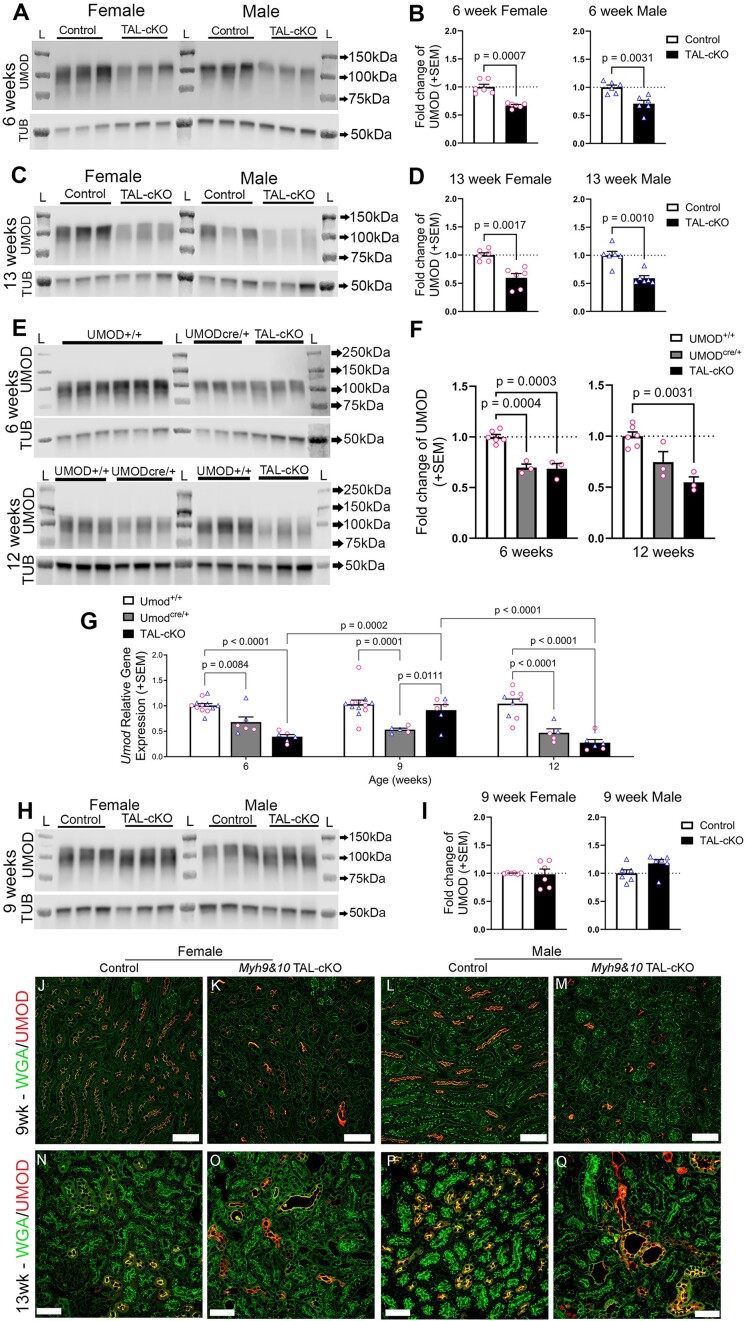
Uromodulin (UMOD) expression and localization in Myh9&10 TAL-cKO mice are influenced by both Umod+/CreERT2 transgene and loss of MYH9&10 proteins. (A-D) Representative immunoblots from whole kidney lysates from female and male kidneys show changes in UMOD expression at 6 and 13 weeks. UMOD protein expression is lower in both male and female kidneys at 6 (A, B) and 13 (C, D) weeks of age compared to control kidneys (n=6 each sex). Tubulin (TUB) is loading control and L = Ladder Lane. (E) Immunoblots of whole kidney lysates from female control (UMOD+/+ and UMODcre/+) and TAL-cKO (TAL-cKO) mice at 6 weeks of age (upper panel) and 12 weeks of age (lower panel) showing changes in UMOD protein levels. (F) Quantification of immunoblots confirms lower UMOD protein levels at 6 and 12 weeks of age in UMODcre/+ and TAL-cKO kidneys compared to UMOD+/+ kidneys. (G) Quantitative PCR analysis of Umod gene expression in female (circles) and male (triangles) kidneys. Significantly lower gene expression is observed in Umodcre/+ kidneys compared to Umod+/+ kidneys at all ages analyzed, indicating that presence of the cre transgene reduces transcript levels. At 6 and 12 weeks of age, expression is also lower in TAL-cKO kidneys compared to Umod+/+ kidneys. At 9 weeks of age there is significantly higher gene expression in TAL-cKO kidneys compared to Umodcre/+ kidneys. In TAL-cKO kidneys, Umod gene expression is significantly higher between 6 and 9 weeks of age and significantly lower from 9 and 12 weeks of age (n≥5 kidneys per genotype and age). (H-I) Representative immunoblot shows that in 9-week-old TAL-cKO male and female kidneys UMOD protein expression is restored to control kidney levels (H, I). (J-M) Fluorescence immunostaining shows variable expression of UMOD in TAL tubules of female and male Myh9&10 TAL-cKO (TAL-cKO) kidneys at 9 weeks of age compared to littermate controls (Myh9f/f; Myh10f/f; Umod+/+). Tubules also show accumulation of UMOD while others show loss of UMOD protein. (N-Q) Representative images from immunostained 13-week old male and female kidneys show dilated tubules with increased UMOD accumulation as well tubules with loss of UMOD protein in TAL-cKO kidneys compared to controls. Scale bars = 50µm (n= 3 kidneys each). Immunoblot results (B, D, I) were normalized to controls (n=3 per blot, n=6 total) and analyzed by unpaired t test with either Welch’s correction or Mann-Whitney test. Immunoblots results (F) were normalized to controls (UMOD+/+) and analyzed independently by age using one-way ANOVA with post-hoc Dunnett’s T3 multiple comparisons test (n=3-6 kidneys each). Transcript data (G) was analyzed by two-way ANOVA with post hoc Tukey’s multiple comparisons test.

Uromodulin is a TAL-specific, GPI-anchored, glycosylated protein, and immunoblotting detects both the glycosylated mature (∼100 kDa) and immature (85-87 kDa) forms.^24,32^ We assessed the expression levels of UMOD in whole kidney lysates from *Myh9&10* TAL-cKO and control mice and our results show reduced UMOD protein levels in both male and female *Myh9&10* TAL-cKO kidneys compared to controls at 6 weeks (Figure 3A and B) and 13 weeks (Figure 3C and D) of age. In our previous pan renal tubular *Myh9&10 Pax8*-cKO mouse model,^24^ we demonstrated significant accumulation and aberrant processing and localization of UMOD with loss of MYH9&10 proteins. We hypothesized that reduction in UMOD protein expression in the *Myh9^F/F^; Myh10^F/F^; Umod*^+/CreERT2^ (*Myh9&10* TAL-cKO) mouse line may be due to the insertion of the *IRES-CreERT2* transgene into the 3′ untranslated region of the endogenous *Umod* gene (JAX 030601). We analyzed *Umod*^+/CreERT2^ (cre-only) and its respective control (*Umod*^+/+^) kidneys via immunoblot assays along with *Myh9&10* TAL-cKO kidneys and confirmed that at 6 and 12 weeks of age, UMOD protein levels are indeed reduced in *Umod*^+/CreERT2^ kidneys compared to *Umod*^+/+^ kidneys (Figure 3E and F). Analysis of transcription using quantitative reverse transcription PCR (qRT-PCR) shows that *Umod* mRNA levels in *Umod*^+/CreERT2^ kidneys are significantly lower compared to *Umod*^+/+^ mice at 6, 9, and 12 weeks of age (Figure 3G), indicating reduced *Umod* gene expression and/or transcript instability in *Umod*^+/CreERT2^ kidneys. Interestingly, while *Umod* mRNA levels are significantly lower in 6- and 13-week-old *Myh9&10* TAL-cKO kidneys compared to *Umod*^+/+^ kidneys (Figure 3G), *Umod* mRNA levels are higher in 9-week-old *Myh9&10* TAL-cKO mouse kidneys compared to *Umod*^+/CreERT2^ kidneys (Figure 3G). Consistent with transcript levels, immunoblots also show UMOD protein levels in *Myh9&10* TAL-cKO kidney lysates at 9 weeks of age are not significantly different from control kidney levels (Figure 3H and I). The relative increase in mRNA level happens only in *Myh9&10* TAL-cKO kidneys at 9 weeks of age and not in *Umod^+/CreERT2^* kidneys, suggesting that loss of *Myh9&10* also has a direct influence on the transcriptional regulation of UMOD expression.

We next assessed the localization pattern of UMOD in *Myh9&10* TAL-cKO and control littermate kidneys to demonstrate any changes in UMOD trafficking to the apical membrane. Immunostained 9-week-old mouse kidney sections show accumulation of UMOD protein within some *Myh9&10* TAL-cKO TAL tubules, while others exhibit UMOD loss (Figure 3K and M) compared to control kidneys, which exhibit localization of UMOD to the apical membrane (Figure 3J and L). In 13-week-old kidneys, the tubular dilation and accumulation of UMOD become severe in some tubules (Figure 3O and Q) compared to controls (Figure 3N and P), while other tubules show loss of protein. Localization of UMOD protein was not affected in 12-week-old *Umod*^+/CreERT2^ and *Umod^+/+^* kidneys (Figures S9C, S9D, and S9G), suggesting that this trafficking defect is related to loss of MYH9&10 proteins.

### ER Stress and UPR Pathway Components and ER Chaperones and ER Tubules Are Altered in *Myh9&10* TAL-cKO Mouse Kidneys

**Figure 4.**
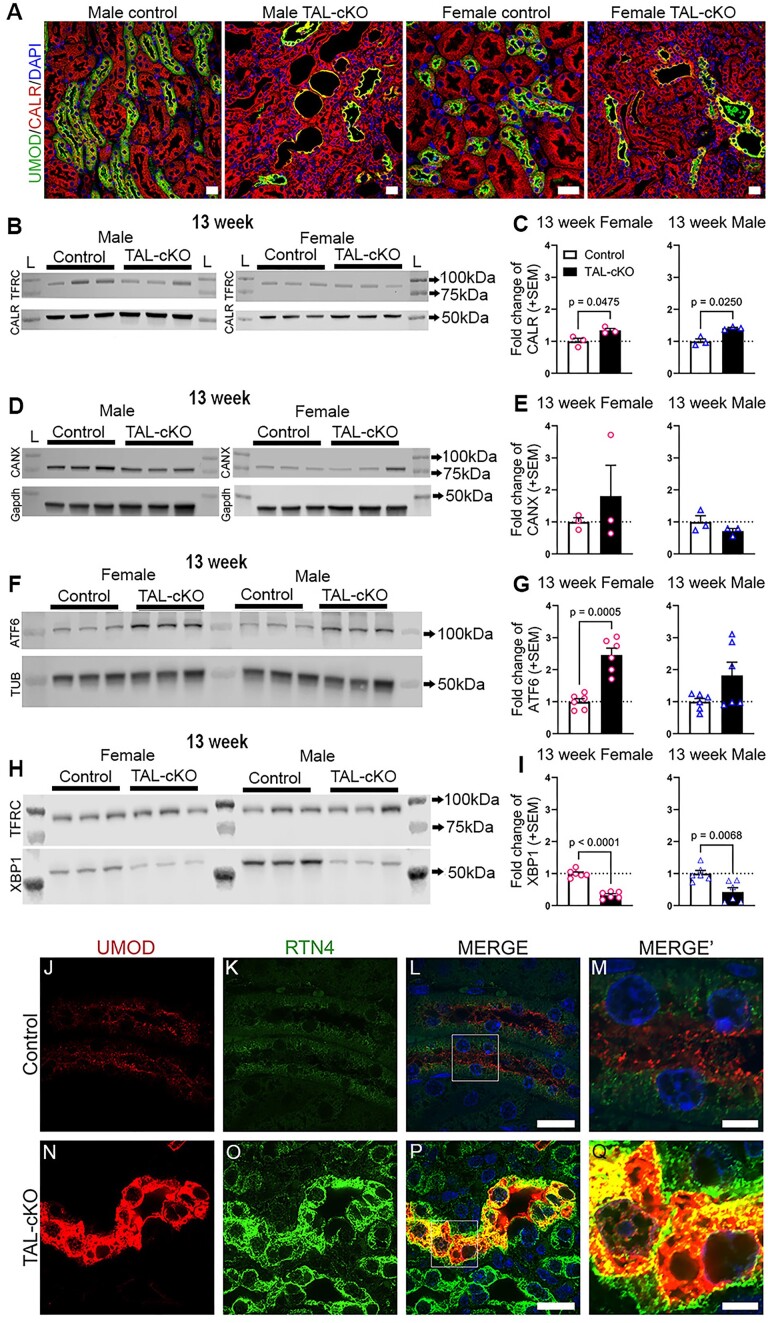
Changes in ER chaperone expression and ER stress/unfolded protein response proteins are observed in *Myh9&10* TAL-cKO mice. (A) Immunostained kidney sections from 13-week-old male and female *Myh9&10* TAL-cKO (TAL-cKO) and littermate controls (*Myh9*^f/f^; *Myh10*^f/f^; *Umod*^+/+^) show higher levels of the ER chaperone calreticulin (CALR) and accumulation within dilated tubules that also have uromodulin (UMOD) accumulation. CALR co-localizes with the accumulated UMOD in TAL-cKO kidneys. There are also dilated tubules with loss of UMOD that show varying levels of CALR expression. Scale bars = 20µm. (n=3 each). (B, C) Western blot analysis of whole kidney lysates shows higher CALR expression in 13-week-old male and female TAL-cKO mice compared to the littermate control kidneys (n=3 each). (D, E) Immunoblots of whole kidney lysates from 13-week-old male and female control and TAL-cKO kidneys show no changes in total calnexin (CANX) protein levels (~78kDa) (n=3 each). (F, G) Immunoblot analysis of whole kidney lysates from 13-week-old male and female control and TAL-cKO kidneys shows ER unfolded protein response (UPR) protein ATF6 levels (~105kDa) are significantly higher in female TAL-cKO kidneys compared to littermate control kidneys (n=6 each). (H, I) Immunoblot analysis of ER UPR response protein XBP1 in whole kidney lysates from 13-week-old TAL-cKO mice shows significantly lower XBP1 protein levels (~40kDa) (indicating apoptotic phase) compared to littermate control kidneys (n=6 each). Transferrin receptor (TFRC), tubulin (TUB), and Gapdh are loading controls and L = Ladder Lane. (J-Q) Super resolution images of 13-week-old control and TAL-cKO kidneys immunostained for UMOD and the endoplasmic reticulum (ER) tubule-associated protein reticulon 4 (RTN4). (J-M) In control kidney TAL cells, UMOD is enriched along the apical (luminal) membrane while RTN4 is present as intracellular puncta. (N-Q) In TAL-cKO kidneys, UMOD accumulates within TAL segments, the cells of which exhibit more intense RTN4 staining that appears to partially colocalize with intracellular UMOD, suggesting retention of UMOD within the ER. Scale bars: L, P = 20µm, M, Q (insets) = 5µm. Immunoblot results were normalized to controls in individual blots (n=3 per blot, n=3-6 total) and analyzed by unpaired t test with either Welch’s correction or Mann-Whitney test.

Aberrant processing and trafficking of mutant UMOD is known to cause tubular kidney disease in humans and mouse models through elevated ER stress and upregulation of the UPR pathway.^28,32,33^ Therefore, we assessed the expression of ER chaperone proteins calreticulin (CALR) and calnexin (CANX) in control and *Myh9&10* TAL-cKO kidneys. Immunostaining for CALR in 13-week-old kidneys shows higher expression levels that colocalize with accumulated UMOD in *Myh9&10* TAL-cKO mouse kidneys compared to littermate controls (Figure 4A). Calreticulin and UMOD do not accumulate in *Umod^+/^*^CreERT2^ kidneys (Figure S9G). Calreticulin immunoblots confirm the observed higher protein expression in *Myh9&10* TAL-cKO kidneys (Figure 4B and C). Immunostaining and immunoblot analysis of *Myh9&10* TAL-cKO kidneys compared to controls do not show significant changes in the expression of CANX (Figure 4D and E). Expression of the ER stress/UPR pathway protein ATF6 is higher in 13-week-old female *Myh9&10* TAL-cKO kidneys than in littermate controls (Figure 4F and G). Similarly, IRE1α/Xbp1 protein levels are found to be significantly lower in 13-week-old *Myh9&10* TAL-cKO kidneys compared to littermate controls, consistent with the upregulation of the apoptotic stage of ER stress/UPR pathway (Figure 4H and I). Super-resolution microscopy of immunostained 13-week-old littermate control kidneys show punctate staining of ER tubule marker reticulon 4 (RTN4) and UMOD within TAL cells, with the majority of UMOD protein localized along the apical membrane and RTN4 observed within the cytosol (Figure 4J-M, Supplemental Movie 1). Thirteen-week-old *Myh9&10* TAL-cKO kidney sections show higher expression of RTN4 within expanded TAL ER tubules where RTN4 partially colocalizes with accumulated UMOD protein, confirming an ER accumulation phenotype (Figure 4N-Q, Supplemental Movie 2).

### Sodium, Potassium, and Chloride Cotransporter Protein Expression Is Reduced in *Myh9&10* TAL-cKO Mouse Kidneys

**Figure 5.**
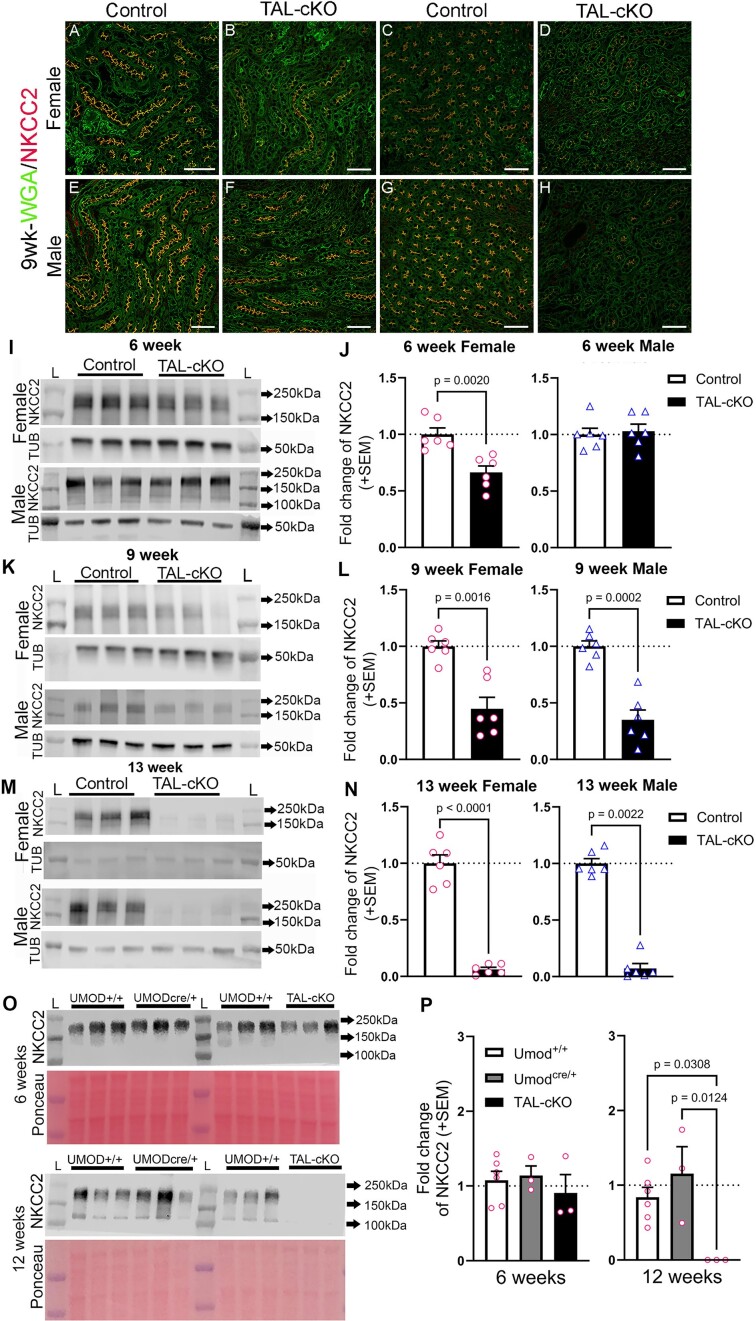
NKCC2 protein expression and apical membrane localization are reduced in *Myh9&10* TAL-cKO kidneys. (A-H) Fluorescence immunohistochemistry shows variable expression of NKCC2 in 9-week-old female and male *Myh9&10* TAL-cKO (TAL-cKO) mice compared to littermate controls (*Myh9*^f/f^; *Myh10*^f/f^; *Umod*^+/+^). (A, C) Expression of NKCC2 in control female TAL tubules is localized to the apical membrane. (B, D) Female TAL-cKO TAL segments show reduction in expression and apical membrane localization of NKCC2, some regions have more pronounced reduction in NKCC2 than others. (E, G) Male control kidneys also have robust expression of NKCC2 on the apical membrane of the TAL-segment. (F, H) TAL-cKO male kidneys have regions of NKCC2 expression that are drastically reduced than other regions of the kidney. Scale bars = 50µm. (n= 3 kidneys each). (I-N) Representative immunoblots for NKCC2 in male and female TAL-cKO and control littermate kidneys show progressive reduction of NKCC2. Quantification of immunoblots shows significant loss of NKCC2 in female TAL-cKO mice at 6, 9, and 13 weeks of age and in male TAL-cKO mice at 9 and 13 weeks of age compared to littermate controls (*Myh9*^f/f^; *Myh10*^f/f^; *Umod*^+/+^), (n=6 each sex). Immunoblot results were normalized to controls in each blot and analyzed by unpaired t test with either Welch’s correction or Mann-Whitney test. Tubulin (TUB) is loading control and L= Ladder Lane. (O) Immunoblots of whole kidney lysates from female control (UMOD+/+ and UMODcre/+) and *Myh9&10* TAL-cKO (TAL-cKO) mice at 6 weeks of age (upper panel) and 12 weeks of age (lower panel) showing NKCC2 protein levels appear unchanged at 6 weeks of age and only reduced in TAL-cKO kidneys compared to UMOD+/+ and UMODcre/+ kidneys at 12 weeks of age. (P) Quantification of immunoblots confirms no change in NKCC2 protein levels at 6 weeks of age and a significantly lower NKCC2 protein levels at 12 weeks of age in TAL-cKO kidneys compared to UMOD+/+ kidneys. No significant difference was observed in NKCC2 protein levels between UMOD+/+ and UMODcre/+ kidneys. Immunoblot results (O) were normalized to their respective controls (UMOD+/+, n=6 kidneys per age) and analyzed by one-way ANOVA with post hoc Dunnett T3 multiple comparisons test.

We previously observed that NKCC2 levels were reduced in pan-renal tubular *Myh9&10 Pax8*-cKO mice with age.^24^ Here, we assessed NKCC2 expression using immunostaining and immunoblot methods in littermate control and *Myh9&10* TAL-cKO mice. Immunostained 9-week-old *Myh9&10* TAL-cKO kidney sections show both lower NKCC2 expression and apical membrane localization compared to control mouse kidneys (Figure 5A-H). We observe focal areas of TAL segments with appreciably less apical membrane-associated NKCC2 (Figure 5D and H), while other areas show only mild to moderate loss (Figure 5B and F) compared to littermate control kidneys (Figure 5A, C, E, and G). Alteration of the apical localization of NKCC2 is not due to a general defect in trafficking of apical membrane proteins, as evidenced by normal expression and localization of ROMK1 in both the TAL and proximal convoluted tubule (PCT) of *Myh9&10* TAL-cKO kidneys (Figure S10). Immunoblot analysis of whole kidney lysates shows significantly lower NKCC2 protein levels in *Myh9&10* TAL-cKO kidneys compared to controls (Figure 5I-N). The extent of NKCC2 loss in *Myh9&10* TAL-cKO kidneys relative to age- and sex-matched controls is greater with age, indicative of compromised trafficking of NKCC2. To assess the influence of the *Umod^CreERT2^* allele on NKCC2 expression, we performed immunoblots of *Umod^+/CreERT2^, Umod^+/+^*, and *Myh9&10* TAL-cKO kidneys (Figure 5O and P). Our results indicate no significant differences in NKCC2 expression levels among the genotypes at 6 weeks of age (Figure 5O and P). However, at 12 weeks of age, *Myh9&10* TAL-cKO kidneys show complete loss of NKCC2 protein compared to *Umod^+/CreERT2^* and *Umod^+/+^* control kidneys (Figure 5O and P). Analysis of *Slc12a1* (NKCC2) mRNA levels in *Umod^+/CreERT2^, Umod^+/+^*, and *Myh9&10* TAL-cKO kidneys showed no differences in mRNA levels in control (*Umod^+/CreERT2^, Umod^+/+^*) mice (Figure S9H). These results indicate that the creERT2-transgene alone does not influence NKCC2 mRNA or protein levels. In *Myh9&10* TAL-cKO kidneys, *Slc12a1* mRNA levels were significantly lower in 12-week-old females and 9-week-old males compared to littermate controls (Figure S9H), perhaps in response to feedback from the pathological changes in the kidney. Taken together, these results confirm the cell autonomous role for MYH9&10 proteins in NKCC2 trafficking and apical membrane localization within the TAL.

### Female *Myh9&10* TAL-cKO Mice Develop Hypernatremia and Morbidity Earlier Than Males

We next examined whether changes in the TAL of *Myh9&10* TAL-cKO mice, including lower UMOD and NKCC2 protein levels, perturb electrolyte homeostasis. Surprisingly, we observe higher serum sodium concentrations starting at 9 weeks of age in female and 12 weeks of age in male *Myh9&10* TAL-cKO mice (Tables 1 and 2). Female *Myh9&10* TAL-cKO mice develop severe hypernatremia at 12 weeks, the time at which they become moribund, with an average serum sodium level of 157.3 ± 0.99 mmol (mean ± SEM) compared to controls at 150.5 ± 0.6 mmol (mean ± SEM) (Table 1). In male *Myh9&10* TAL-cKO mice, higher serum sodium is evident starting at 12 weeks and becomes severe at 20 weeks with mean values of 162.1 ± 3.7 mmol (mean ± SEM) in *Myh9&10* TAL-cKO mice compared to 151.9 ± 1.34 mmol (mean ± SEM) in control mice (Table 2). Male *Myh9&10* TAL-cKO mice experience sudden death between 24 and 25 weeks of age. We also observe significantly higher serum potassium concentrations in female *Myh9&10* TAL-cKO mice at 12-weeks of age (Table 1).

**Table 1.** Mean serum sodium and potassium concentrations in female *Myh9&10 TAL-cKO* and littermate control mice. P values of less than 0.05 are shown in bold.

Age (weeks)	Control *n* =	*Myh9&10* TAL-cKO *n* =	Serum Sodium ± SEM		*P* value	Serum Potassium ± SEM		*P* value
			Control	*Myh9&10* TAL-cKO		Control	Control *Myh9&10* TAL-cKO	
6	8	10	150.1 ± 0.6	150.1 ± 0.4	0.9718	4.60 ± 0.08	4.57 ± 0.09	0.8111
9	18	13	150.3 ± 0.4	152.8 ± 1.0	**0.0289**	4.19 ± 0.09	4.28 ± 0.11	0.5421
12	8	9	150.5 ± 0.7	157.3 ± 1.0	**<0.0001**	4.19 ± 0.19	4.71 ± 0.08	**0.0329**

Statistical significance determined by unpaired *t* test with Welch’s correction.

**Table 2.** Mean serum sodium and potassium concentrations in male *Myh9&10 TAL-cKO* and littermate control mice. P values of less than 0.05 are shown in bold.

Age (weeks)	Control *n* =	*Myh9&10* TAL-cKO *n* =	Serum Sodium ± SEM		*P* value	Serum Potassium ± SEM		*P* value
			Control	*Myh9&10* TAL-cKO		Control	Control *Myh9&10* TAL-cKO	
6	7	8	150.4 ± 0.9	149.0 ± 0.5	0.1912	4.54 ± 0.08	4.51 ± 0.10	0.8094
9	15	22	150.0 ± 0.4	150.6 ± 0.4	0.2525	4.42 ± 0.10	4.52 ± 0.07	0.4173
12	6	9	150.8 ± 0.4	154.1 ± 1.0	**0.0092**	4.38 ± 0.11	4.58 ± 0.06	0.1635
20	9	7	151.9 ± 1.3	162.1 ± 3.8	**0.0348**	4.46 ± 0.11	4.77 ± 0.10	0.0578

Statistical significance determined by unpaired *t* test with Welch's correction.

Hypernatremia in *Myh9&10* TAL-cKO mice is an unexpected result, as there is a decline in NKCC2 protein expression and apical membrane localization in these mice starting at 9 weeks of age. Loss of hydration could account for hypernatremia; however, we ruled out water loss as we did not observe changes in urine osmolality (Figure S11), water intake, or urine volume (Table S8) in female *Myh9&10* TAL-cKO mice at any of the analyzed time points. In male *Myh9&10* TAL-cKO mice, we observed higher water intake and urine volume starting at 16 weeks of age (Table S9). Urinary sodium and potassium excretion in male *Myh9&10* TAL-cKO mice is also reduced at 20 weeks of age (Figure S11 and Table S9).

### Sodium Chloride Cotransporter Protein Expression Is Altered in *Myh9&10* TAL-cKO Mouse Kidneys

**Figure 6.**
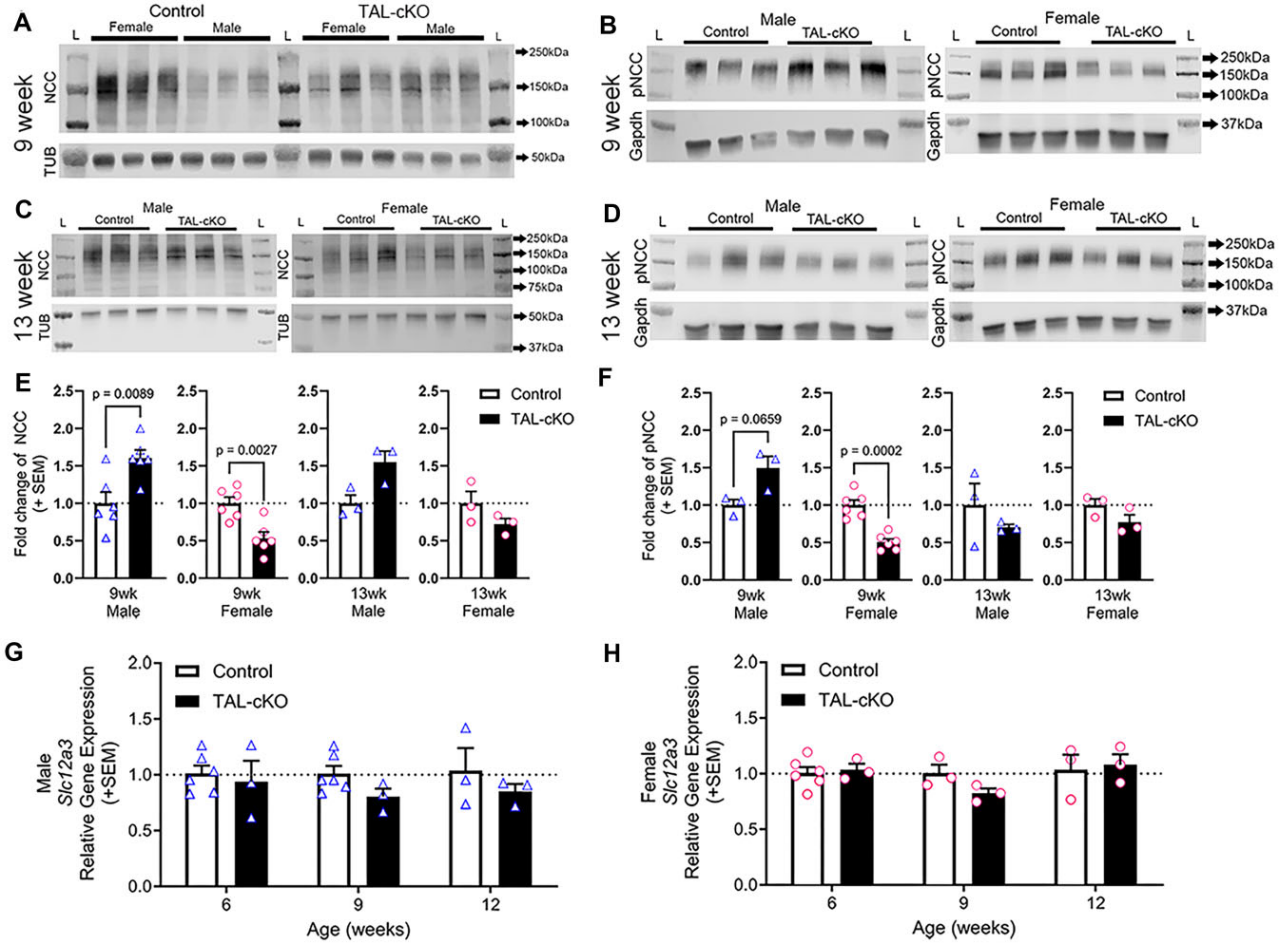
Sodium chloride cotransporter expression is differentially altered in female versus male *Myh9&10* TAL-cKO kidneys. (A) Representative immunoblot from 9-week-old whole kidney lysates shows lower NCC expression in female *Myh9&10* TAL-cKO (TAL-cKO) kidneys and higher expression in male TAL-cKO kidneys compared to littermate controls (*Myh9*^f/f^; *Myh10*^f/f^; *Umod*^+/+^). The blot also confirms that female control mice have higher NCC levels compared to the male control mice. (B) Similar trends were observed for phosphorylated-NCC (pNCC) where TAL-cKO male kidneys had higher pNCC levels and female kidneys had lower pNCC levels compared to littermate controls. (C) Representative immunoblot from 13-week-old whole kidney lysates shows persistently higher NCC expression in male and lower NCC expression in female TAL-cKO kidneys compared to littermate controls. (D) Immunoblot from 13-week-old whole kidneys lysates showing pNCC expression in male and female control and TAL-cKO mice. Tubulin (TUB) and Gapdh are loading controls for the immunoblots and L = ladder Lane. (E) Quantification of NCC immunoblots shows significantly higher NCC expression in male TAL-cKO (triangles) kidneys and significantly lower NCC expression in female TAL-cKO (circles) kidneys at 9 weeks of age compared to littermate controls. No significant difference is seen in 13-week-old male or female TAL-cKO kidney NCC levels compared to littermate controls (n=3-6 each age and sex). (F) Quantification of pNCC immunoblots shows significantly higher pNCC expression in male TAL-cKO (triangles) kidneys and significantly lower pNCC expression in female TAL-cKO (circles) kidneys at 9 weeks of age compared to littermate controls. No significant difference is seen in 13-week-old male or female TAL-cKO kidney pNCC levels compared to controls (n=3-6). Immunoblot results were normalized to their respective controls in the blot and analyzed by unpaired t test with Welch’s correction or Mann-Whitney test (n=3 per blot, n=3-6 total). (G, H) Quantitative PCR of NCC (Slc12a3) gene expression shows no significant difference at 6, 9, or 12 weeks of age (n=3-6). Lack of significant difference in gene expression was determined by two-way ANOVA.

To determine whether hypernatremia in female and male *Myh9&10* TAL-cKO mice was due to distal nephron adaptation, we analyzed the primary DCT sodium chloride cotransporter NCC. We confirmed sex-based differences in NCC expression in mice as reported previously,^34,35^ with control female mice expressing more total NCC protein than control male mice (Figure 6A). Our results also showed that at 9 weeks of age, NCC levels in male *Myh9&10* TAL-cKO mice were significantly higher than controls (Figure 6A). However, female *Myh9&10* TAL-cKO kidneys showed lower NCC protein expression at 9 weeks of age compared to littermate controls (Figure 6A). At 13 weeks of age, NCC expression levels trend higher in male *Myh9&10* TAL-cKO mice compared to controls, while NCC trends lower in females, but neither value is statistically significant (Figure 6C and E). We also performed immunoblot analysis of phosphorylated NCC (pNCC) to assess cotransporter activity. Levels of pNCC were significantly lower in female *Myh9&10* TAL-cKO mice and elevated in male *Myh9&10* TAL-cKO mice at 9 weeks of age compared to littermate controls (Figure 6B and F). No significant differences were observed in pNCC levels at 13 weeks of age in either male or female *Myh9&10* TAL-cKO mice compared to littermate controls (Figure 6D and F). Transcript analysis using qRT-PCR reveals that changes in NCC protein levels are not due to changes in mRNA expression in *Myh9&10* TAL-cKO kidneys (Figure 6G and H). Ultimately, lower NCC/pNCC protein levels at 9 weeks of age do not explain the observed hypernatremia in female *Myh9&10* TAL-cKO mice, as lower NCC levels would be predicted to result in lower urinary excretion of sodium. Furthermore, higher NCC/pNCC levels at 9 weeks of age in *Myh9&10* TAL-cKO male mice (Figure 6A) fail to explain the delayed onset of hypernatremia observed at 12 weeks of age (Table 2). Interestingly, considering there is a statistically significant loss of potassium in the urine of female *Myh9&10* TAL-cKO mice compared to littermate controls at 9 weeks of age (*P* value = 0.029) (Figure S11 and Table S8), we hypothesize that the collecting duct segment might be responsible for the hypernatremia in female *Myh9&10* TAL-cKO mice.

### Epithelial Sodium Channel Gamma Subunit Expression Is Higher in *Myh9&10* TAL-cKO Mice

**Figure 7.**
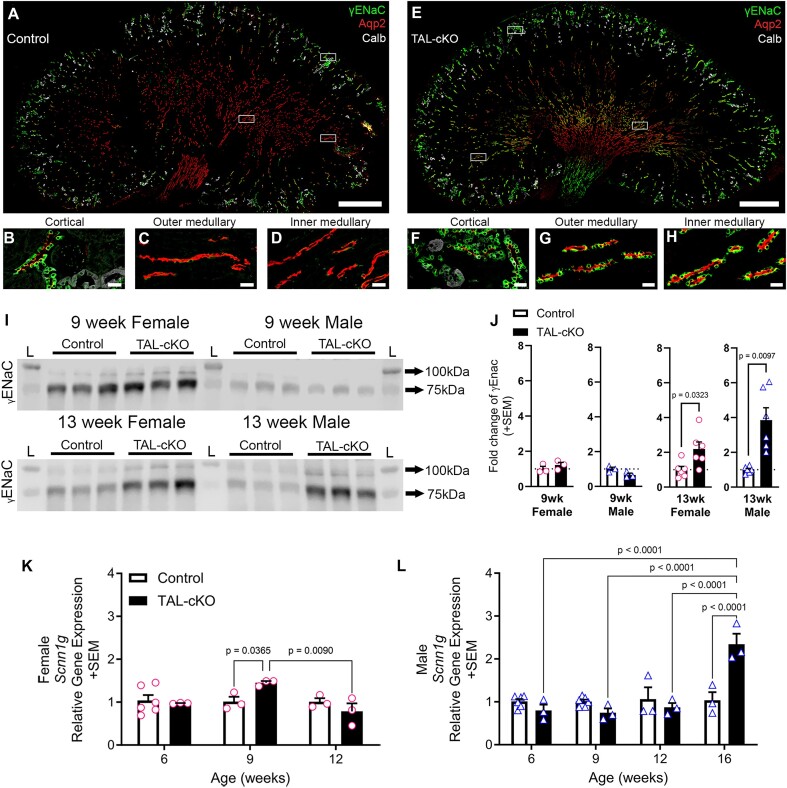
Epithelial sodium channel gamma subunit is expressed in *Myh9&10* TAL-cKO medullary collecting duct**s**. (A) Immunostaining of 13-week-old control (*Myh9*^f/f^; *Myh10*^f/f^; *Umod*^+/+^) male kidney shows γENaC expression in calbindin- and AQP2-positive cells of the cortex and outer medulla. White boxes are enlarged regions in panels B-D. (B) γENaC is expressed in the cortical distal tubules and collecting ducts. (C) Little γENaC expression is observed in the outer medullary collecting ducts. (D) Inner medullary collecting ducts of control animals do not express γENaC. (E) Immunostaining of 13-week-old male *Myh9&10* TAL-cKO (TAL-cKO) kidney sections shows higher γENaC expression in cortical epithelial cells and unexpected expression in the inner and outer medullary collecting ducts. White boxes are enlarged regions in panels F-H. (F) γENaC expression in TAL-cKO mice is higher in cortical distal tubules and collecting ducts. (G) γENaC expression is observed within the outer medulla of TAL-cKO mice. (H) γENaC expression is observed within the inner medulla of TAL-cKO mice (n=3-4 kidneys each). Scale bars in A,E=1000µm. Scale bars in B-D and F-H=25µm. (I) Western blot analysis of whole kidney lysates shows no difference in γENaC expression (~75kDa cleaved form) in 9-week-old male or female TAL-cKO mice compared to controls. Whole kidney lysates from 13-week-old male and female TAL-cKO mice show higher γENaC expression compared to controls. Ponceau stain was used for loading control assessment (see supplemental data). (J) Immunoblot quantification shows significantly higher γENaC protein in both male (triangles) and female (circles) 13-week-old TAL-cKO mice compared to controls (n=6 each sex). (K) γENaC (*Scnn1g*) gene expression is significantly higher in 9-week-old female TAL-cKO mice compared to controls and compared to 12-week-old TAL-cKO female mice. (L) *Scnn1g* gene expression is higher in 16-week-old male TAL-cKO mice compared to controls. Immunoblot results were normalized to controls and analyzed by age using unpaired t test with either Welch’s correction or Mann-Whitney test (n=3 per blot, n=3-6 total). Gene expression data was analyzed by two-way ANOVA with post-hoc Tukey’s test (n≥3 per condition).

We hypothesize that higher ENaC activity may contribute to the observed hypernatremia in *Myh9&10* TAL-cKO female mice at 9 weeks of age accompanied by higher secretion of K+, and so we evaluated ENaC expression. Murine ENaC is a heterotrimeric complex formed of subunits (α, β, and γ) and is expressed in the aldosterone-sensitive DCT and cortical collecting duct.^36^ We assessed the expression of the γ subunit (γENaC) using immunostaining and immunoblotting methods in control and *Myh9&10* TAL-cKO kidneys (Figure 7). Our results show expression of γENaC within calbindin and AQP2-positive cells in the cortex, as well as in a few AQP2-positive calbindin-negative tubules in the outer medullary region of 13-week-old control kidneys (Figure 7A-D and Figure S12A-D). Epithelial sodium channel gamma subunit is not expressed in the IMCD3 epithelium (Figure 7A and D) and is typically restricted only to the cortical tubules (Figure 7A and B); however, in both male and female *Myh9&10* TAL-cKO kidneys, these cells uncharacteristically express high levels of γENaC protein (Figure 7H and S12H). In *Myh9&10* TAL-cKO kidneys, we also observe higher expression of γENaC in the calbindin- and AQP2-positive tubules of the cortex, as well as in many AQP2-positive tubules of the outer and inner medullary regions [Figure 7E-H and Figure S12 (insets)].

Additionally, we used super-resolution microscopy to visualize the intracellular distribution of γENaC in control and *Myh9&10* TAL-cKO kidneys. Z-stack movies (Supplemental Movies 3-4) and maximum intensity projections of the z-stacks (Figure S12I and S12J) from both control and *Myh9&10* TAL-cKO cortical collecting ducts show expression of the majority of γENaC within intracellular punctate structures similar to previous reports.^37,38^ In medullary collecting ducts of control kidneys (Supplementary Movie 5 and Figure S12I), there is no observable γENaC expression. However, in *Myh9&10* TAL-cKO medullary collecting ducts, we observe greater γENaC expression with the majority localized to intracellular puncta (Supplementary Movie 6 and Figure S12J).

Gamma-ENaC activity is controlled through proteolytic cleavage by furin as well as subsequent processing by additional proteases.^36^ Immunoblots show that uncleaved γENaC has an apparent molecular weight of ∼85 kDa by SDS-PAGE, while the cleaved subunit is approximately 75 kDa. Immunoblots from 9-week-old female and male *Myh9&10* TAL-cKO kidneys did not show a change in γENaC expression compared to littermate controls (Figure 7I and J). However, at 13 weeks of age, immunoblots show higher expression of both the cleaved and uncleaved forms of γENaC in both male and female *Myh9&10* TAL-cKO mice compared to littermate controls (Figure 7I and J). Furthermore, N-linked glycosylation was enzymatically removed via PNGaseF treatment of whole kidney lysates in order to more clearly identify the bands representing cleaved and uncleaved γENaC to assess whether proteolytic cleavage of γENaC^29^ differed between littermate control and *Myh9&10* TAL-cKO kidneys. Results confirm the presence of higher expression levels of γENaC subunit in *Myh9&10* TAL-cKO, but we do not observe significant changes in the cleavage of γENaC in *Myh9&10* TAL-cKO kidneys (Figure S12). Next, we analyzed γENaC (*Scnn1g*) subunit transcript levels in whole kidneys using qRT-PCR. Our results show significantly higher expression of *Scnn1g* mRNA in 9-week-old female (Figure 7K) and 16-week-old male (Figure 7L) *Myh9&10* TAL-cKO kidneys compared to littermate controls. Although immunostaining and immunoblots show greater γENaC levels in both male and female *Myh9&10* TAL-cKO mice at 13 weeks of age, transcript analysis indicates that higher *Scnn1g* gene expression occurs earlier in female *Myh9&10* TAL-cKO mice (at 9 weeks) compared to male *Myh9&10* TAL-cKO mice (at 16 weeks), potentially contributing to the sex-specific differences seen in the onset of hypernatremia.

### Acute Amiloride Treatment Confirms Higher ENaC Activity in *Myh9&10* TAL-cKO Mice

**Figure 8.**
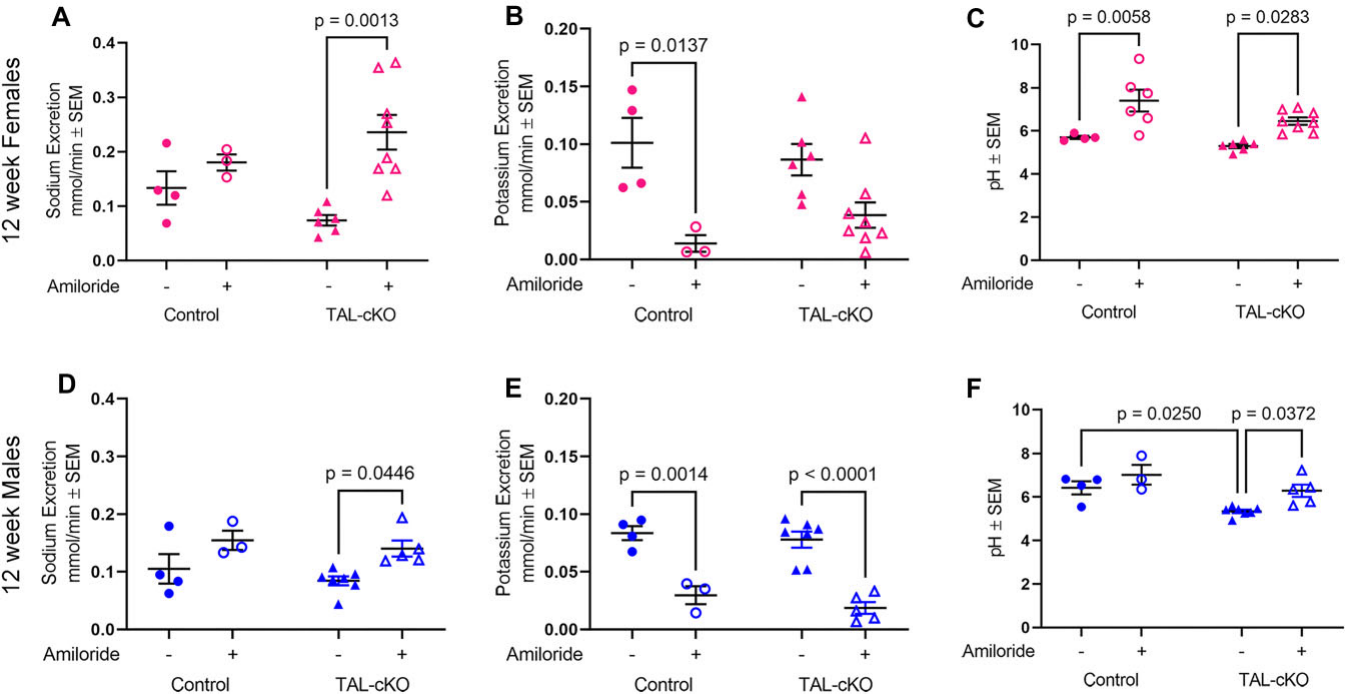
Acute amiloride treatment confirms higher ENaC activity in *Myh9&10* TAL-cKO mice. (A-F) To assess ENaC function, 12-week-old female and male control and*Myh9&10* TAL-cKO (TAL-cKO) mice were administered vehicle (-) or amiloride (+). (A) Sodium excretion is significantly higher in female TAL-cKO mice that received amiloride compared to those that received vehicle, while sodium excretion is not significantly different between control mice that received amiloride or vehicle. (B) Potassium excretion is significantly lower in female control mice treated with amiloride compared to those that received vehicle, but not significantly different between female TAL-cKO mice treated with amiloride and those that received vehicle. (C) Urine pH is higher in control and TAL-cKO females treated with amiloride compared to those receiving vehicle. (D) Sodium excretion is higher in male TAL-cKO mice treated with amiloride compared to those treated with vehicle only. No significant difference is seen in control animals treated with amiloride compared to vehicle. (E) Potassium excretion is significantly lower in control and TAL-cKO male mice receiving amiloride compared to those treated with vehicle. (F) Urine pH is significantly higher in amiloride-treated TAL-cKO male mice and is significantly lower in TAL-cKO vehicle mice compared to control vehicle mice. N ≥ 3 for each group in amiloride studies. Two-way ANOVA post-hoc Tukey’s test with multiple comparisons was used to analyze effects of genotype and amiloride treatment on urine parameters.

We acutely treated control and *Myh9&10* TAL-cKO mice with amiloride to confirm a role for ENaC in mediating excessive sodium reabsorption and retention in these mice. Amiloride blocks ENaC activity, promotes natriuresis, and has an indirect effect on potassium efflux resulting in lowered potassium excretion.^39^ Urine chemistry analysis shows significantly higher sodium excretion in female *Myh9&10* TAL-cKO mice that received amiloride treatment compared to the littermate control group that received amiloride (Figure 8A). As expected, potassium excretion was significantly lower in the amiloride-treated female control mice but not in *Myh9&10* TAL-cKO mice; the change in potassium excretion was more robust in control mice treated with amiloride than in vehicle-treated *Myh9&10* TAL-cKO mice (Figure 8B) indicating a blunted anti-kaliuretic response. Urine pH is significantly higher in female control and *Myh9&10* TAL-cKO mice that received amiloride compared to mice that received only vehicle (Figure 8C). Male *Myh9&10* TAL-cKO mice at 12 weeks of age also showed changes in sodium and potassium excretion (Figure 8D and E, respectively) with amiloride treatment compared to the control group treated with amiloride; however, the difference in sodium excretion was not as profound compared to 12-week-old *Myh9&10* TAL-cKO females that received amiloride. This suggests that male *Myh9&10* TAL-cKO mice have more moderate hypernatremia at 12 weeks than the female *Myh9&10* TAL-cKO mice, also confirmed by serum sodium measurements (Tables 1 and 2). Amiloride treatment in Male *Myh9&10* TAL-cKO mice also results in higher urinary pH compared to vehicle treatment (Figure 8F).

### Altered Expression of Osmolality-Responsive Genes in *Myh9&10* TAL-cKO Kidneys

**Figure 9.**
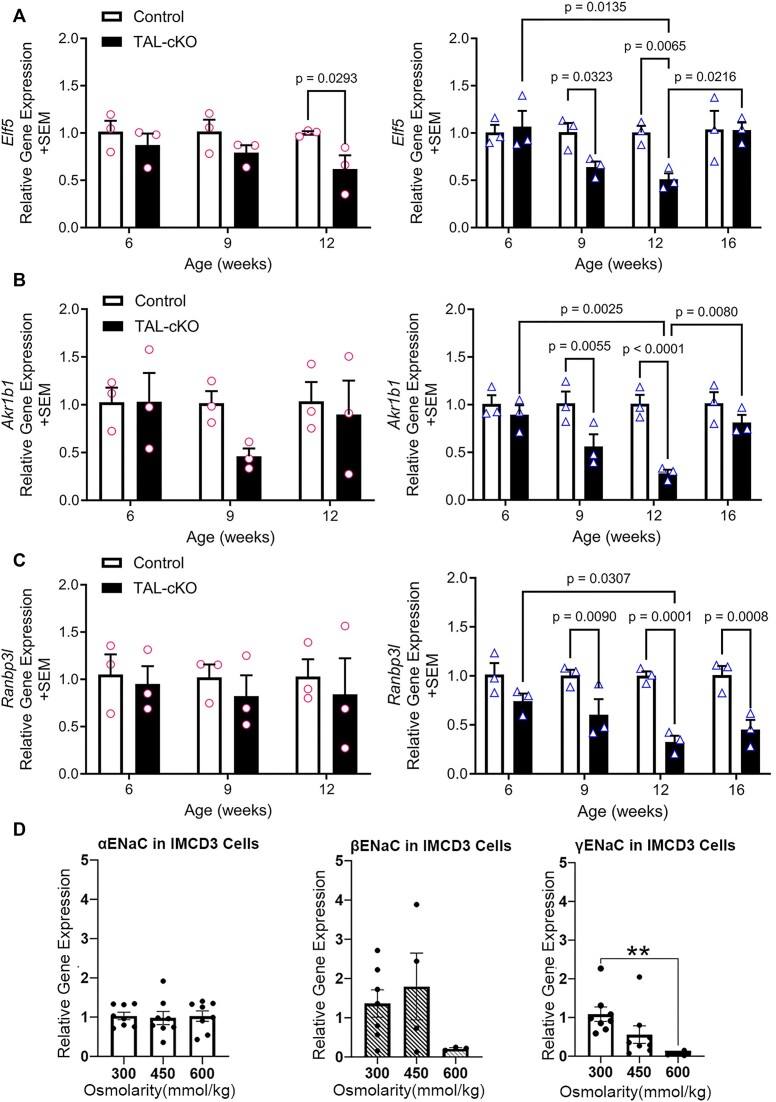
Osmo-responsive genes show altered expression profile in *Myh9&10* TAL-cKO kidneys and ENaC subunits are osmo-responsive in IMCD3 cells highlighting the cellular plasticity of collecting duct epithelium. (A) *Elf5* gene expression is lower in female (circles) *Myh9&10* TAL-cKO (TAL-cKO) kidneys at 12 weeks of age and in male (triangles) TAL-cKO kidneys at 9 and 12 weeks of age compared to control kidneys (*Myh9*^f/f^; *Myh10*^f/f^; *Umod*^+/+^). (B) *Akr1b1* gene expression did not show statistically significant changes in female kidneys but trended lower in 9 weeks female TAL-cKO compared to control kidneys. In male TAL-cKO kidneys, *Akr1b1* gene expression was significantly reduced at 9, and 12-weeks of age, which increased at 16 weeks compared to controls. (C) *Ranbp3l* expression is significantly lower in 6, 9, 12- and 16-week-old male TAL-cKO mouse kidneys while it varies in female TAL-cKO mice; no significant reduction was observed in female TAL-cKO mice compared to littermate controls (*Myh9*^f/f^; *Myh10*^f/f^; *Umod*^+/+^), (n=3 each). Two-way ANOVA with post-hoc Tukey’s test was used to determine differences in gene expression between control and TAL-cKO mice. (D) IMCD3 cells cultured in 300mOsm media show detectable levels of *Scnn1g* (γENaC) gene expression. *Scnn1g* expression is significantly lower in cells cultured in 600mOsm media compared to those cultured in 300mOsm media. The expression of the αENaC and βENaC subunits are less influenced by changes in extracellular osmolarity. n=6-8 biological replicates. One-way ANOVA with post-hoc Dunnett’s T3 multiple comparisons was used for statistical analysis of gene expression.

Considering that the TAL segment is critical for generation of an osmotic gradient along the corticomedullary axis, we hypothesized that the expansion of γENaC expression into the inner medulla of *Myh9&10* TAL-cKO kidneys is part of a direct response to reduced interstitial osmolality in the medulla secondary to TAL deregulation. Several genes undergo transcriptional regulation in response to extracellular osmolality.^30^ Elf5 is a collecting duct principal cell-specific transcription factor that is known to be upregulated under hyperosmolar conditions in IMCD3 cells.^30,40–42^ Akr1b1 (aldose reductase) protects cells exposed to hyperosmolar conditions^43^ and Ranbp3l was recently identified to be highly upregulated in IMCD3 cells cultured in hypertonic conditions.^30^ We examined whether these 3 genes are downregulated in *Myh9&10* TAL-cKO kidneys. We observe markedly lower *Elf5* transcript in 12-week-old female and in 9- and 12-week-old male *Myh9&10* TAL-cKO mice compared to controls (Figure 9A). *Akr1b1* transcript expression in male *Myh9&10* TAL-cKO mice is reduced at 9 and 12 weeks of age (Figure 9B), and the reduction in *Elf5* and *Akr1b1* transcripts is restored at 16 weeks in male *Myh9&10* TAL-cKO mice (Figure 9A and B). *Akr1b1* transcript levels trended lower in 9-week-old female TAL-cKO mice compared to controls but is not statistically significant (Figure 9B). The expression of *Ranbp3l* is significantly lower in 9-, 12-, and 16-week-old male *Myh9&10* TAL-cKO mice compared to littermate controls (Figure 9C), but no differences were seen in female TAL-cKO mice. The downregulation of hyperosmolality-responsive genes is consistent with the idea that medullary interstitial osmolality is lower in the kidneys of *Myh9&10* TAL-cKO male mice. In female *Myh9&10* TAL-cKO mice, while *Elf5* is significantly lower at 12 weeks and *Akr1b1* is trending lower at 9 weeks of age (Figure 9A and B), the changes in gene expression are not as pronounced as seen in males. The robust reduction in the male *Myh9&10* TAL-cKO mice in the osmo-responsive genes compared to females may be the result of higher expression of γENaC between 6 and 9 weeks of age, leading to other confounding signals.

Because urea contributes to the activity of the urinary concentrating mechanism, we also assessed the expression of UT-A1 and UT-A3 urea transporters in control and *Myh9&10* TAL-cKO kidneys using immunostaining methods with 2 different antibodies directed against the c-terminus (L194) and n-terminus (L446) of the transporters, respectively. We did not observe robust changes in the expression of UT-A1. However, there is lower UT-A3 expression in 13-week-old *Myh9&10* TAL-cKO kidneys compared to littermate control kidneys (Figure S13).

To confirm that changes in extracellular osmolality have the potential to alter the expression of *Scnn1g* (γENaC) transcripts, we utilized the IMCD3 cell culture system.^44^ Inner medullary collecting duct cells were gradually acclimated to culture media with increased osmolality (450 and 600 mmol/kg compared to the physiological 300 mmol/kg). There were no statistically significant changes in *Scnn1a* (αENaC) mRNA levels between the 3 different conditions (Figure 9D). *Scnn1b* (βENaC) subunit expression trended lower in cells cultured in higher osmolality, but this did not reach statistical significance (Figure 9D). Interestingly, *Scnn1g* (γENaC) expression is significantly lower upon exposure to higher osmolality conditions (Figure 9D). These results indicate that extracellular osmolality is indeed capable of regulating ENaC subunit gene expression in an in vitro model of the medullary collecting duct. Hence, the higher relative osmolality that is normally present in the medullary and papillary regions of the kidney may typically suppress ENaC expression, while conditions that reduce interstitial osmolality may inhibit suppression and lead to ENaC expression.

## Discussion

Our previous work established a role for NM2 proteins, MYH9 and MYH10, in cargo trafficking within the renal tubular epithelium utilizing a pan-renal tubular, *Myh9&10 Pax8*-cKO mouse model.^24^ To better understand the cell autonomous role for NM2 proteins specifically in the TAL segment, we generated a tamoxifen-inducible, TAL segment-specific *Myh9* and *Myh10* conditional inactivation mouse model using the *UMOD-IRES->CreERT2* JAX mouse strain (*Myh9&10* TAL-cKO). Histology, BUN, serum creatinine, and tubular injury marker (NGAL) and increase in CD3+ cells reveal disease progression similar to that of our previous *Myh9&10 Pax8*-cKO mouse model.^24^ Furthermore, analysis of TAL-specific cargoes shows loss of apical membrane localization and lower NKCC2 protein levels at 9 and 13 weeks of age in *Myh9&10* TAL-cKO kidneys, but not in *Umod^+/CreERT2^* kidneys. This supports the idea that loss of NKCC2 is a direct consequence of loss of MYH9 and MYH10 (Figure 5).

Interestingly, we observe variations in *Umod* mRNA (Figure 3) and protein expression in *Myh9&10* TAL-cKO mice (Figure 3). We confirm that modification of the 3′ regulatory region of the *Umod* allele results in lower transcript expression and/or transcript stability (Figure 3). The recently characterized *SLC12a1-IRES->CreERT2* mouse model also showed lower transcript and protein levels associated with modification of the endogenous gene via insertion of the *IRES->CreERT2* transgene in the 3′ untranslated region of the *Slc12a1* gene.^45^ However, lower expression of UMOD did not impair kidney function in *Umod^+/CreERT2^* mice compared to littermate controls (Figure S9 and Tables S3-S5). Furthermore, our results reveal both transient UMOD mRNA increases at 9 weeks of age (Figure 3) and compromised trafficking of UMOD protein along with intracellular accumulation of UMOD within some TAL tubules of *Myh9&10* TAL-cKO mice at 9 and 13 weeks of age (Figure 3). We also observe the upregulation of the ER chaperone protein CALR and the ER stress/UPR markers ATF6 and spliced XBP1 (Figure 4). ER tubule expansion and higher expression of RTN4 (ER tubule marker) colocalized with accumulated UMOD is also evident in *Myh9&10* TAL-cKO kidneys at 13 weeks of age (Figure 4, Supplemental Movie 2). These results indicate that while *IRES->CreERT2* transgene influences the UMOD mRNA transcription, there are also transcriptional and trafficking defects arising from loss of MYH9&10 proteins in the TAL tubules.

Intriguingly, we observe a striking sexual dimorphism in morbidity in female *Myh9&10* TAL-cKO mice along with early development of hypernatremia (Figure 1B, Table 1). Since the development of hypernatremia seems contradictory to the loss of NKCC2, we initially suspected volume loss might be responsible. However, evaluation of metabolic input and output of *Myh9&10* TAL-cKO mice shows no significant changes in urine osmolality (Table S8). This could be attributed to the progressive nature of the chronic kidney disease (Figure 1C and D) and perhaps lower glomerular filtration rate in these mice. Early reports have provided evidence for the production of hypertonic urine with reduction in fluid delivered to the diluting segment (TAL) without any influence from anti-diuretic hormone (vasopressin).^46^ We also did not observe any significant changes in copeptin levels in *Myh9&10* TAL-cKO mice compared to littermate controls (Figure S12). We hypothesized that loss of NKCC2 could result in aberrant adaptation of the distal nephron and collecting duct, resulting in hypernatremia. Careful characterization of NCC in the distal nephron and ENaC in the collecting duct of *Myh9&10* TAL-cKO mice indicates a sexually dimorphic response to lower levels of NKCC2 protein (Figure 6, Figure 7, Figure S12).

Significant evidence exists in the literature demonstrating sex-specific differences in the expression of several transporters and channels in the mammalian kidney.^34,47–50^ Furthermore, elegant work by several laboratories using diuretics and transgenic knockout rodent models have highlighted the adaptability of the distal nephron and collecting duct segments to preserve sodium and potassium balance.^47,48,51^ Although *Slc12a3* (NCC) mRNA levels are similar across all genotypes tested (Figure 6G and H), our results show altered regulation of NCC protein in male and female *Myh9&10* TAL-cKO mice. Male *Myh9&10* TAL-cKO mice show higher NCC protein levels at 9 weeks of age, adapting to loss of NKCC2 in the TAL segment. However, in female *Myh9&10* TAL-cKO mice, NCC and pNCC levels decrease at 9 weeks of age compared to littermate controls (Figure 6E and F). We confirmed that although *UMOD-IRES->CreERT2* allele is active in DCT1 segment, we do not observe loss of MYH10 in tubular segments other than the TAL in *Myh9&10* TAL-cKO mouse kidneys (Figure S1). This might be due to the slow turnover of MYH10 protein.^52,53^ Furthermore, based on the low expression and localization pattern of MYH10,^13^ we speculate that its functional role in DCT is minimal and does not influence NCC trafficking. Consistent with this, in our assessment of pan-renal tubular *Myh9&10 Pax8*-cKO mice,^24^ we found no changes in pNCC localization. MYH9 protein is not expressed in the DCT segments and is limited to a few PCT segments and TAL in murine kidneys.^13^ Ultimately, the signals responsible for lower NCC protein levels in female *Myh9&10* TAL-cKO mice are unclear and further experiments are required to better understand NCC regulation in both sexes.

Loss of NCC in mice (NCC knockout) is known to trigger pendrin-mediated chloride reabsorption to ensure ENaC-mediated sodium retention.^51^ Moreover, in SPAK knockout mice, loss of NCC phosphorylation also triggers an increase in the number of pendrin-positive intercalated cells as well as ENaC upregulation.^54^ However, we did not observe any major changes in pendrin localization and expression in *Myh9&10* TAL-cKO mice (Figure S14). These reports support the notion of upregulation of ENaC in response to lower sodium reabsorption in the DCT. Interestingly, we also observe higher expression of γENaC, particularly in the IMCD3 cells of *Myh9&10* TAL-cKO kidneys, which under normal conditions (control kidneys) do not express detectable levels of ENaC protein (Figure 7). Our work demonstrates expression of γENaC in adult kidney medullary collecting duct cells in response to deregulated trafficking of UMOD and NKCC2 in the TAL. Moreover, acute amiloride treatment of *Myh9&10* TAL-cKO mice results in higher sodium excretion, confirming that the sodium retention in these mice is a direct effect of ENaC activity (Figure 8A and D). In addition, the anti-kaliuretic effect of amiloride is blunted in female *Myh9&10* TAL-cKO mice compared to littermate controls, possibly suggesting that the uncharacteristic presence of ENaC in the medullary region impairs its influence over potassium secretion (Figure 8B). We attribute uncharacteristic γENaC expression in the medullary collecting duct cells to loss of the osmotic gradient along the corticomedullary axis in the kidney, which we hypothesize is a direct consequence of compromised TAL function due to lower NKCC2 protein levels. We speculate that IMCD cells are transitioning into a more “cortical collecting duct—like” cell type due to the lack of high interstitial osmolality typically present in the medullary region. Consistent with this, mRNA transcript levels of γENaC are significantly lower in IMCD3 cells cultured under higher osmolality (600 mmol/kg) conditions compared to the lower osmolality conditions (300 mmol/kg) (Figure 9D). In addition, we observe changes in osmolality-responsive genes *Akr1b1, Ranbp3l* in male *Myh9&10* TAL-cKO mice and *Elf5* in male and female TAL-cKO mice.

Greater ENaC expression in the cortical and medullary collecting ducts rather than sustained NCC upregulation is suggestive that distal nephron adaptation does not have the capacity to compensate for chronic TAL dysfunction. Although NCC protein levels are higher in male *Myh9&10* TAL-cKO mice, this is not sustained, and eventually ENaC expression is initiated, causing hypernatremia. In contrast, females downregulate NCC expression potentially in response to the loss of potassium in the urine at 9 weeks of age, which is an interesting association that warrants further characterization (Figure S11 and Table S8). Consistent with this, a recent report showed that female mice are more sensitive to low potassium diet and develop collecting duct-associated kidney disease.^55^ Our results suggest that females are more sensitive to loss of NKCC2 and compromised TAL function, highlighting the importance of sex-inclusive characterization of renal transgenic knockout animal models and making this an essential focus for future research. Additional experiments are also warranted to further test whether the observed functional adaptability seen in IMCD cells in response to TAL dysfunction is mediated by lower interstitial osmolality in the renal medulla. We attribute the observed sexual dimorphism in *Myh9&10* TAL-cKO mice to the adaptive capacity of the distal nephron sodium cotransporter NCC in response to TAL dysfunction. In turn, this results in temporal differences in upregulation of γENaC in the medullary collecting ducts. Our work provides compelling evidence for cell plasticity in the IMCD^41^ and may yield insights into impaired natriuretic response to prolonged loop diuretic treatment.^56,57^

Ultimately, this work has confirmed a cell autonomous role for myosin II motor proteins MYH9 and MYH10 in regulating TAL cargo trafficking using the nephron segment-specific *Myh9&10* TAL-cKO mouse model. Actin-associated motor proteins are known to regulate short-range trafficking within cells.^11,52,58^ Given the complex organelle architecture within TAL cells,^4,5,8^ we are presently exploring the role of MYH9&10 proteins in regulating specialized ER, plasma membrane, and mitochondria contact sites using novel electron and light microscopy methods.^59^

## Supplementary Material

zqae048_Supplemental_Files

## Data Availability

All data pertaining to this article are available within the main document and supplementary materials. Uncut western blots are added as supplementary document.
