# Supplementary material for: Thick Ascending Limb Specific Inactivation of Myh9 and Myh10 Myosin Motors Results in Progressive Kidney Disease and Drives Sex-specific Cellular Adaptation in the Distal Nephron and Collecting Duct
Source: Function (Oxf). 2024 Nov 5;6(1):zqae048. doi: 10.1093/function/zqae048 (PMC11815580; doi:10.1093/function/zqae048)
Supplement: zqae048_Supplemental_Files [file zqae048_supplemental_files.zip › Supplemental Document Final 10302024.pdf]

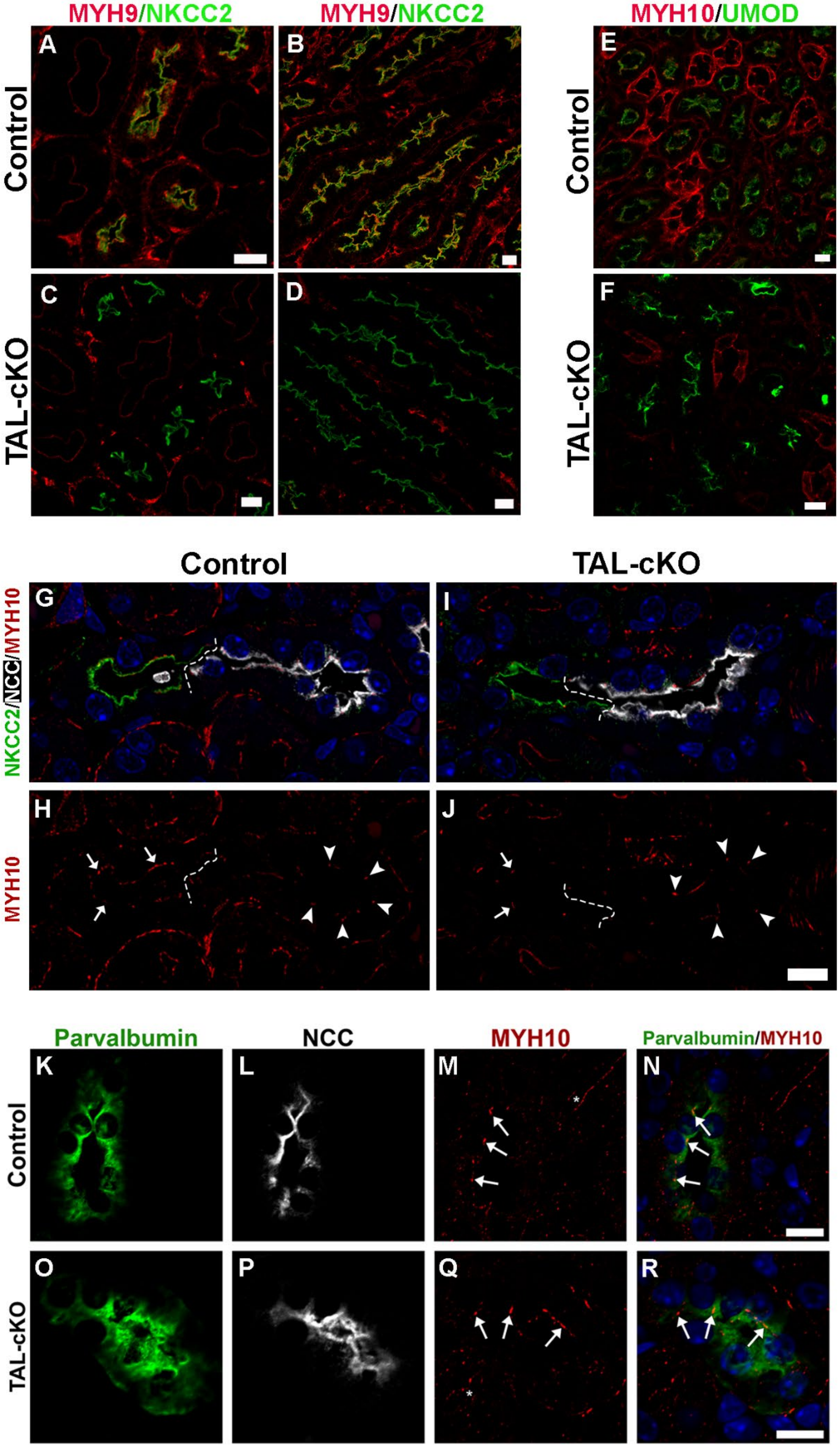
MYH9/NKCC2
MYH9/NKCC2
MYH10/UMOD
Control
TAL-cKO
A
B
C
D
E
F
Control
TAL-cKO
NKCC2/NCC/MYH10
MYH10
G
H
I
J
Parvalbumin
NCC
MYH10
Parvalbumin/MYH10
K
L
M
N
O
P
Q
R

Figure S1. Loss of MYH9 and MYH10 proteins is segment specific and apparent in the TAL epithelium of *Myh9&10* TAL-cKO kidneys. A, B. MYH9 protein localizes to the apical and basolateral membranes in NKCC2-positive control TAL cells. **C, D.** Tamoxifen treated *Myh9&10* TAL-cKO (TAL-cKO) mice have loss of MYH9 protein within the TAL-segment, identified by NKCC2 staining, by 9 weeks of age. **E.** MYH10 is localized to the apical and basolateral membranes of UMOD-positive littermate control (*Myh9*$^{f/f}$; *Myh10*$^{f/f}$; *Umod*$^{+/+}$) TAL cells. **F.** Treatment with tamoxifen results in loss of MYH10 expression in UMOD-positive TAL cells of TAL-cKO kidneys. **G-J.** Co-staining of NKCC2 and NCC was used to identify the transition from TAL to DCT1, respectively, for assessment of MYH10 expression. **G, H.** MYH10 protein localizes to discrete regions of the apical membrane within NCC-positive (DCT1) and NKCC2-positive (TAL) tubules in 9-week-old littermate control kidneys. MYH10 protein localizes to the apical membrane of the TAL (H, arrows) and to apical cell-cell boundaries in DCT1 (H, arrowheads). **I, J.** TAL-cKO TAL cells exhibit markedly lower MYH10 staining (J, arrows) while DCT1 cells maintain expression of MYH10 at 9 weeks of age. Dashed lines approximately mark the TAL-DCT1 boundary. **K-R.** DCT1 cells were additionally identified by immunostaining against parvalbumin and NCC. **K-N.** In littermate control kidneys at 9 weeks of age, DCT1 cells exhibit MYH10 protein localization to the apical region (M, arrows). **O-R.** In TAL-cKO kidneys at 9 weeks of age, MYH10 protein expression in DCT1 cells is maintained with a similar apical localization to littermate control cells (Q, arrows). Asterisks (M,Q) denote lumen of adjacent tubule. Scale bars = 50μm (A-F) and 10μm (G-R). n=3-4 kidneys each.

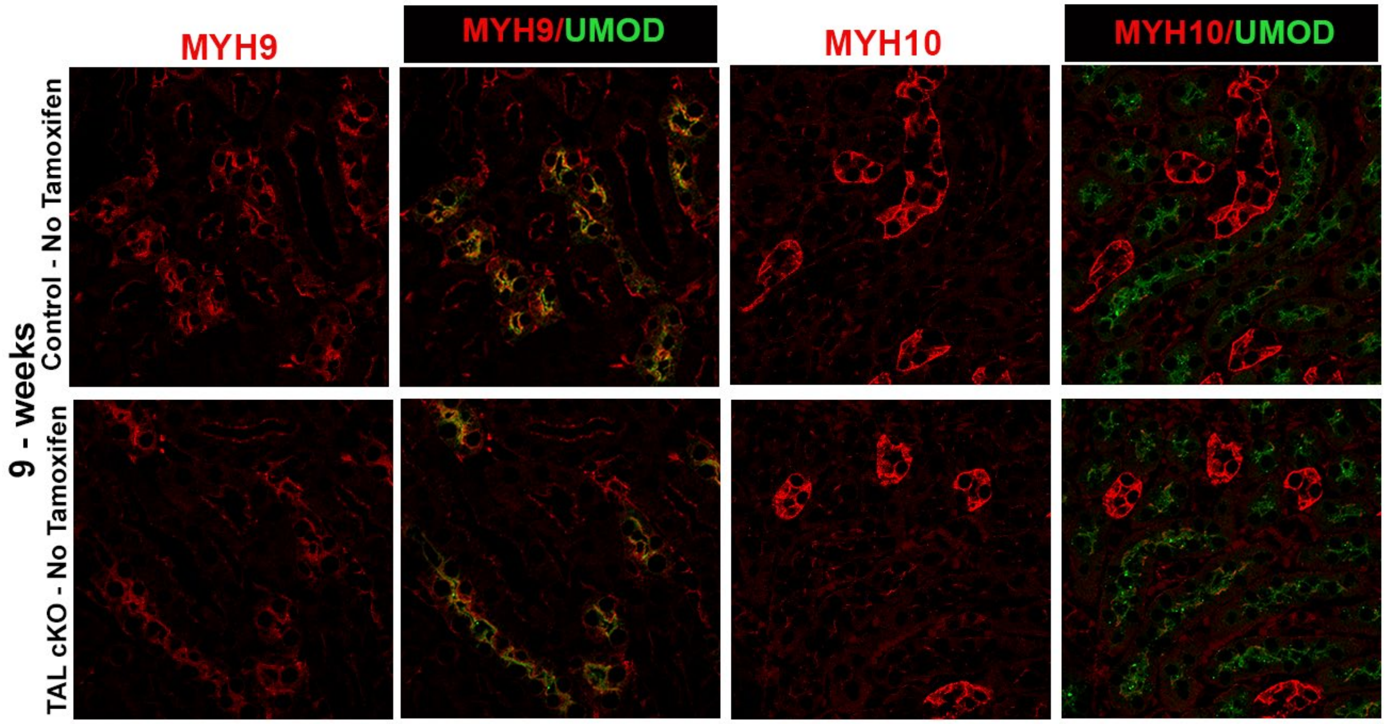

Figure S2. Immunostaining of 9-week-old littermate control (*Myh9*$^{f/f}$; *Myh10*$^{f/f}$; *Umod*$^{+/+}$) and *Myh9&10* TAL-cKO (TAL-cKO) kidneys from mice which did not receive tamoxifen. MYH9 and MYH10 protein localization and expression appear unchanged in untreated (No Tamoxifen) TAL-cKO kidneys compared to untreated (No Tamoxifen) littermate controls.

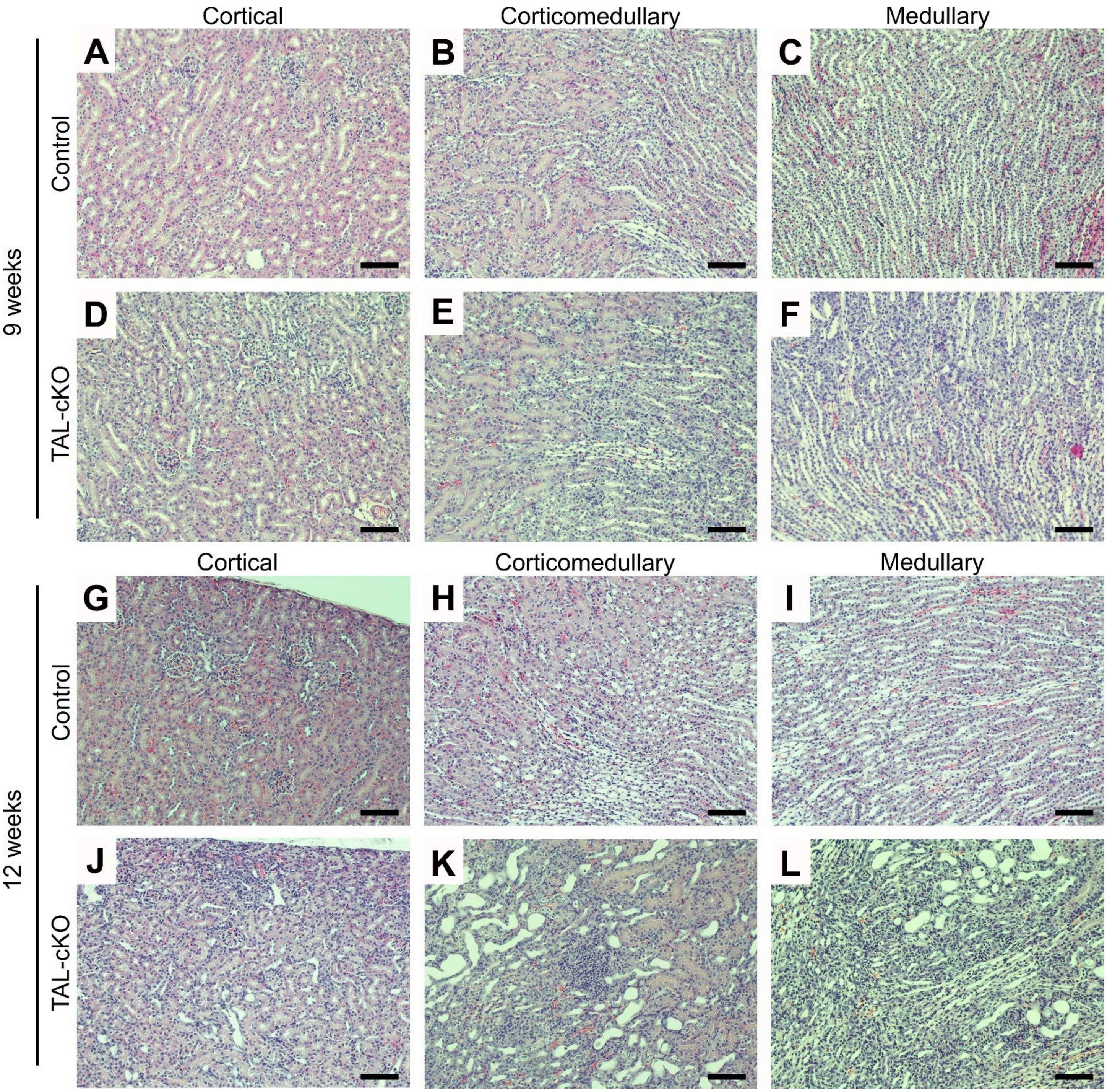

Figure S3. H&E staining of male *Myh9&10* TAL-cKO kidneys shows progressive histological changes.

A-C. Representative 9-week-old littermate control (*Myh9*$^{f/f}$; *Myh10*$^{f/f}$; *Umod*$^{+/+}$) kidney images from cortical, corticomedullary, and medullary regions show healthy renal tissue. **D-F.** 9-week-old *Myh9&10* TAL-cKO mice have minimal changes in tubular diameter and cellular density. **G-I.** Representative images of 12-week-old littermate control kidney from the cortical, corticomedullary, and medullary regions also show healthy renal tissue. **J-L.** Evaluation of 12-week-old male *Myh9&10* TAL-cKO kidneys shows extensive tubular dilation in the corticomedullary and medullary regions with focal areas of dilation in the cortex. Infiltrating cells are also observed in all three regions. Scale bars = 100µm (n=3-4 kidneys each).

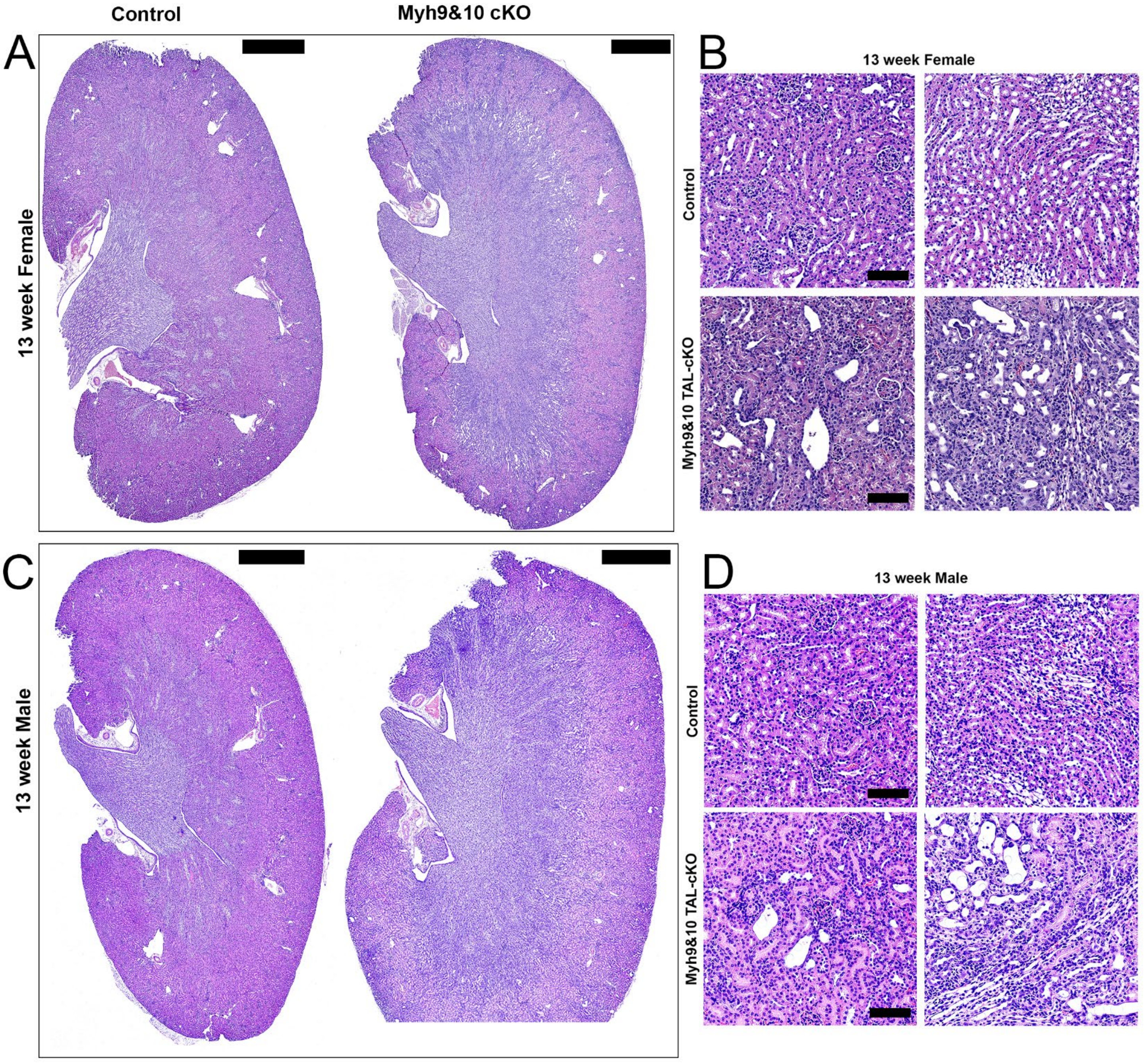

Figure S4. Slide scanner images and selected high magnification insets of 13-week-old littermate control (*Myh9*$^{f/f}$; *Myh10*$^{f/f}$; *Umod*$^{+/+}$) and *Myh9&10* TAL-cKO (TAL-cKO) male and female kidneys. **A, C**. Large slide scanner image of 13-week old littermate control and TAL-cKO mice show pathological changes in the cKO kidneys compared to the littermate controls. Dilated tubules and cellular infiltration are apparent in both male and female TAL-cKO kidneys. **B, D.** Regions of interest were chosen from the slide scanner image and high magnification images show the damage to the tubules and interstitium in TAL-cKO kidneys. Scale bars: A,C = 1000µm; B,D = 100µm.

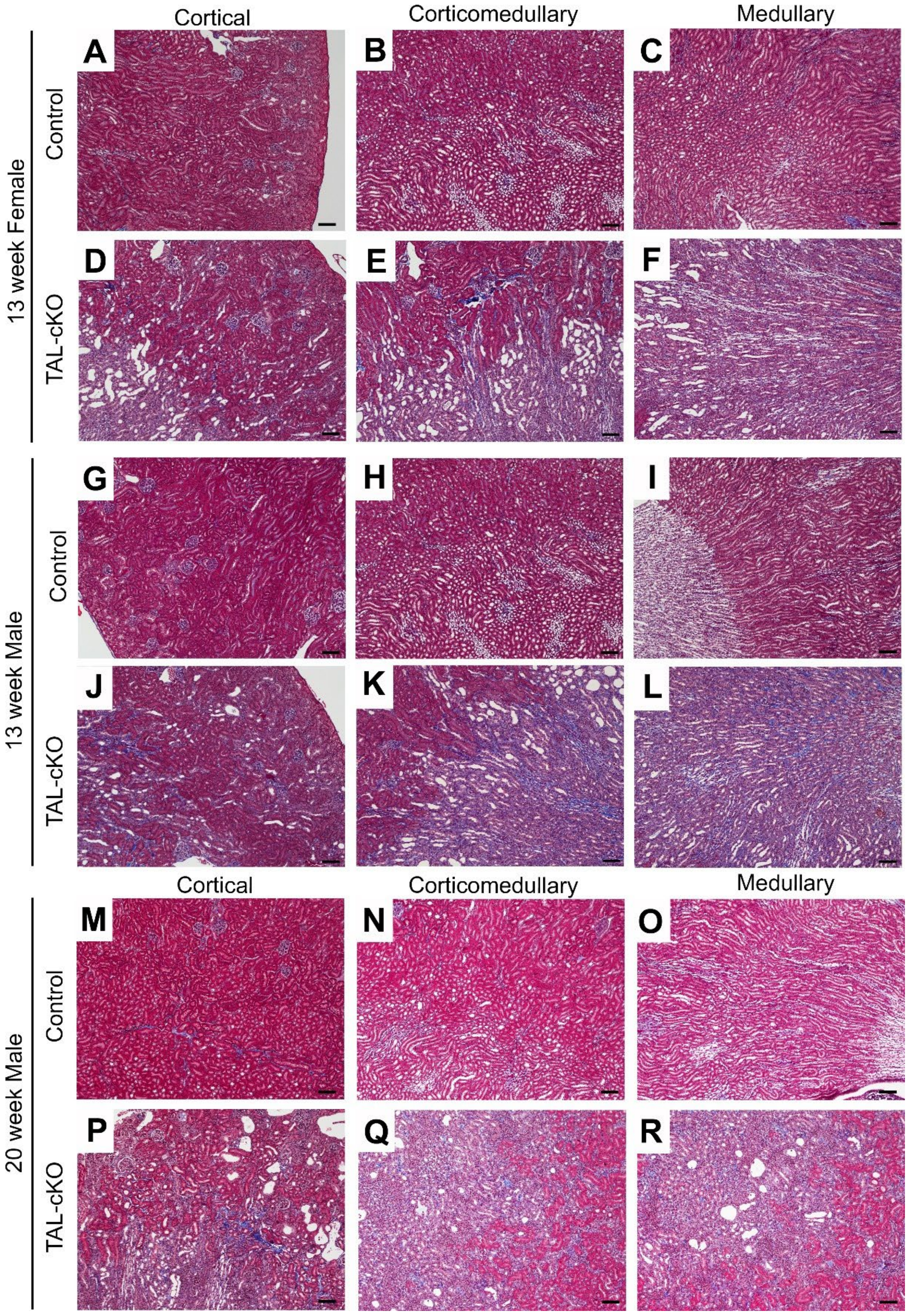

Cortical
Corticomedullary
Medullary
13 week Female
Control
TAL-cKO
A
B
C
D
E
F
13 week Male
Control
TAL-cKO
G
H
I
J
K
L
Cortical
Corticomedullary
Medullary
20 week Male
Control
TAL-cKO
M
N
O
P
Q
R

Figure S5. Masson's trichrome staining shows fibrosis in *Myh9&10* TAL-cKO mice. A-C. Representative images of female 13-week-old littermate control (*Myh9*$^{f/f}$; *Myh10*$^{f/f}$; *Umod*$^{+/+}$) kidneys show healthy renal tissue. **D-F.** Female 13-week-old *Myh9&10* TAL-cKO (TAL-cKO) kidneys show fibrosis as evidenced by the presence of positive collagen staining throughout the cortical, corticomedullary and medullary regions. **G-I.** Representative images of male 13-week-old littermate control kidneys show healthy renal tissue. **J-L.** 13-week-old male TAL-cKO mice exhibit collagen staining throughout the kidney indicating fibrosis. **M-O.** Representative images of male 20-week-old littermate control kidneys show healthy tubular structure. **P-R.** TAL-cKO kidneys from 20-week-old males show extensive tubular damage and fibrosis. Scale bars = 100µm. (n=3 kidneys each).

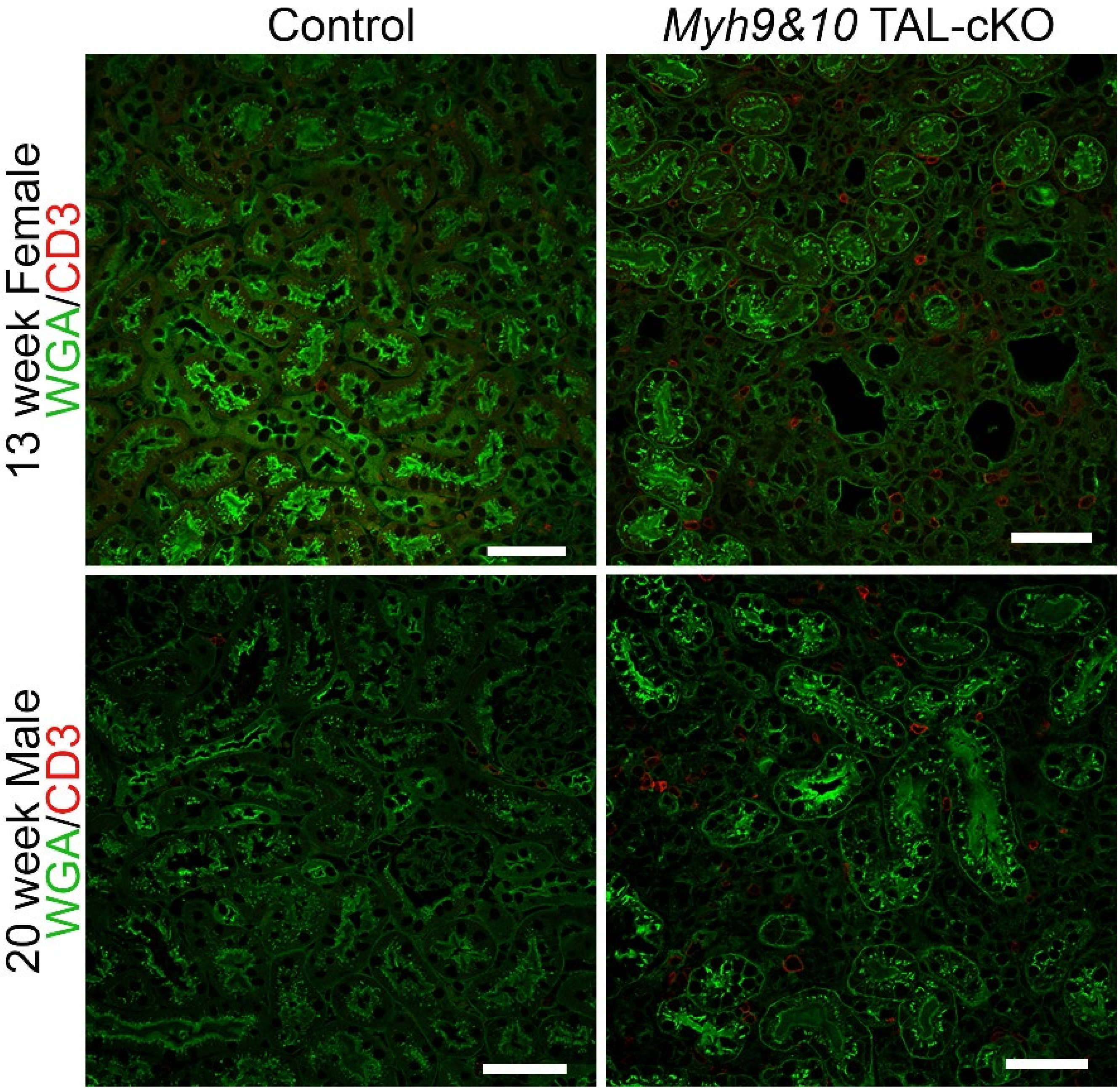

Figure S6. ***Myh9&10*** **TAL-cKO kidneys exhibit higher numbers of CD3+ cell infiltration.**

Representative images of CD3 immunostaining in 13-week-old littermate control (*Myh9*$^{f/f}$; *Myh10*$^{f/f}$; *Umod*$^{+/+}$) and *Myh9&10* TAL-cKO female and 20-week-old littermate control and *Myh9&10* TAL-cKO male kidneys show significantly more CD3-positive cells in *Myh9&10* TAL-cKO kidneys. Scale bar = 50µm. (n=3-4 kidneys each).

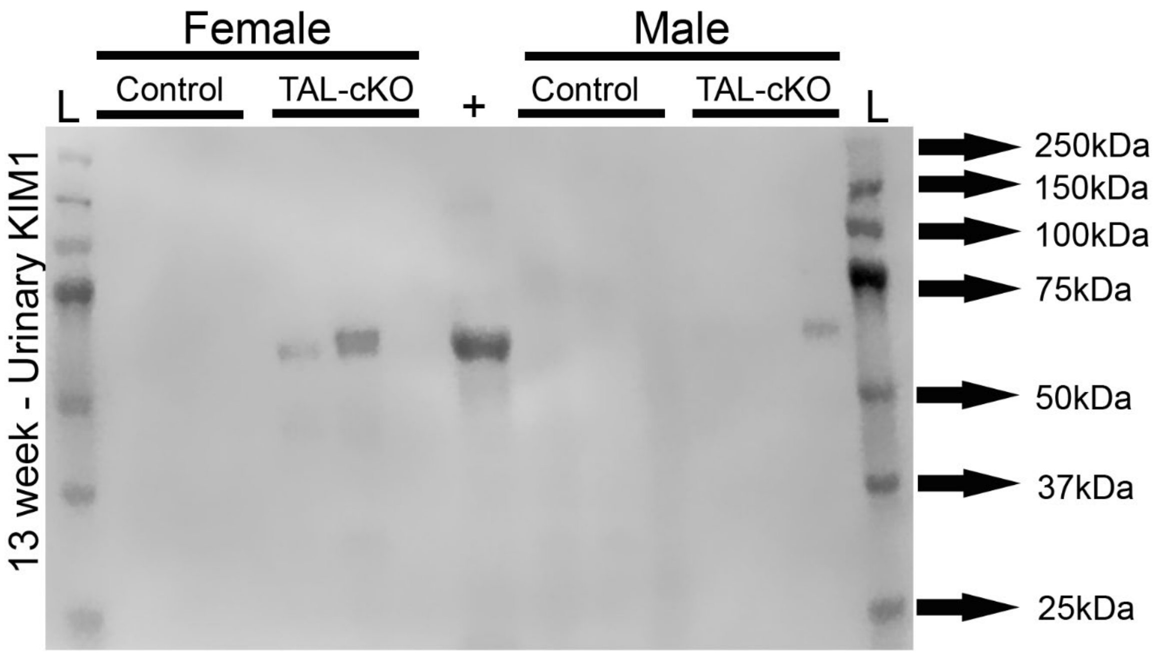

Figure S7. Some *Myh9&10* TAL-cKO mice have detectable levels of proximal tubular injury marker KIM1 in the urine at 13 weeks of age.

Urine collected from 24-hour metabolic cages was concentrated, quantified, and analyzed by immunoblot. In addition to the positive control (+) urine from 12-week-old *Myh9&10 Pax8*-cKO mice, two female and one male 13-week-old *Myh9&10* TAL-cKO mice had KIM1 present in their urine. All the littermate controls (*Myh9*$^{f/f}$; *Myh10*$^{f/f}$; *Umod*$^{+/+}$) and the remaining three *Myh9&10* TAL-cKO mice did not have detectable levels of KIM1 in the urine (n=3 for each sex).

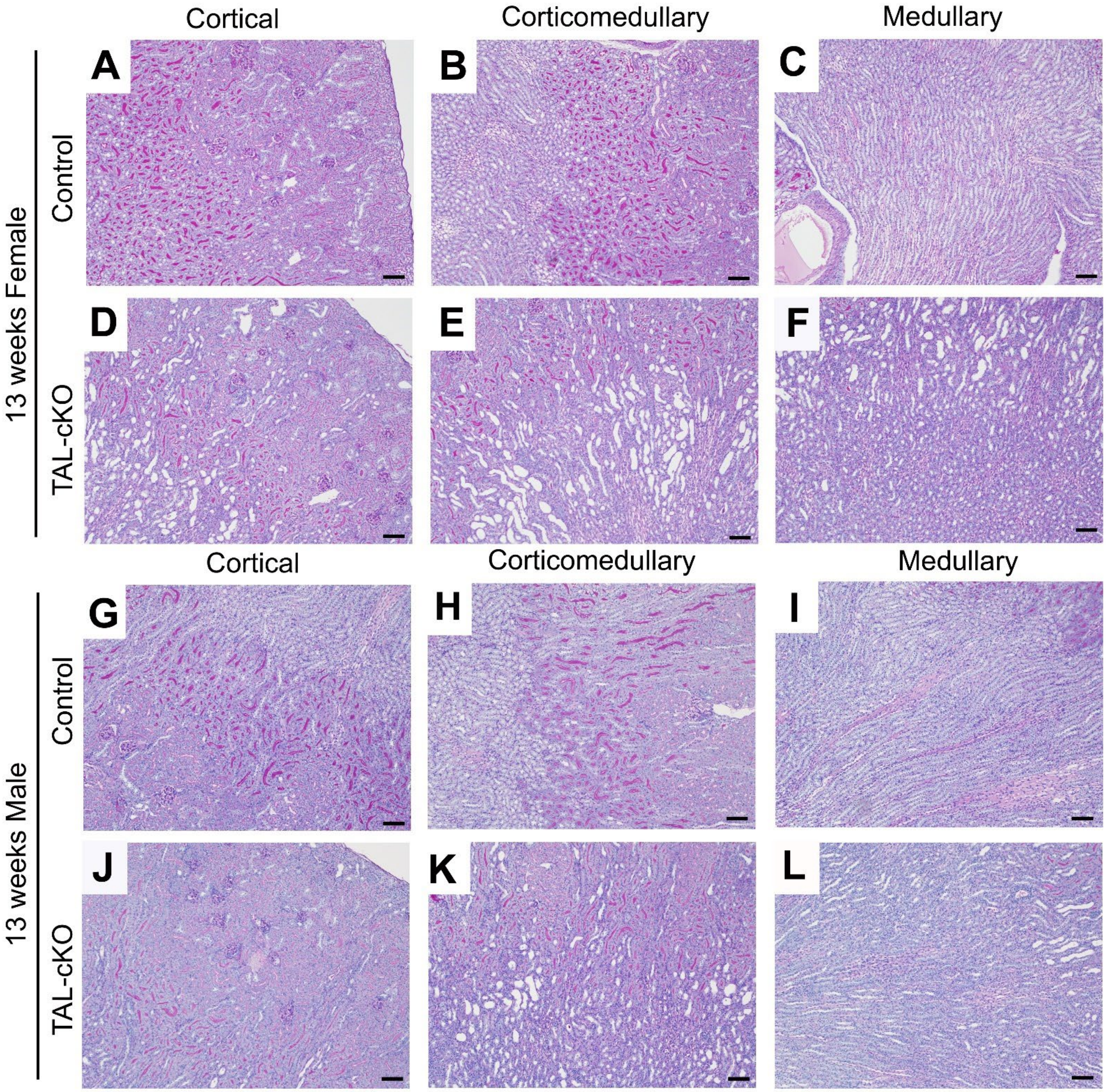

Figure S8. Periodic acid Schiff (PAS) staining shows lower brush border intensity in *Myh9&10* TAL-cKO kidneys.

A-C. Representative images of 13-week-old female littermate control (*Myh9*$^{f/f}$; *Myh10*$^{f/f}$; *Umod*$^{+/+}$) kidneys show robust staining of the proximal tubule brush border in the cortical and corticomedullary regions. **D-F.** Female 13-week-old *Myh9&10* TAL-cKO mice show lower intensity of brush border staining in some proximal tubules, but no loss of brush borders. **G-I**. Representative images of 13-week-old male littermate control kidneys also show robust proximal tubule brush border staining in the cortical and corticomedullary regions. **J-L.** 13-week-old male *Myh9&10* TAL-cKO mice show lower intensity in PAS staining within some tubules. Scale bars = 100µm. (n=3 kidneys each).

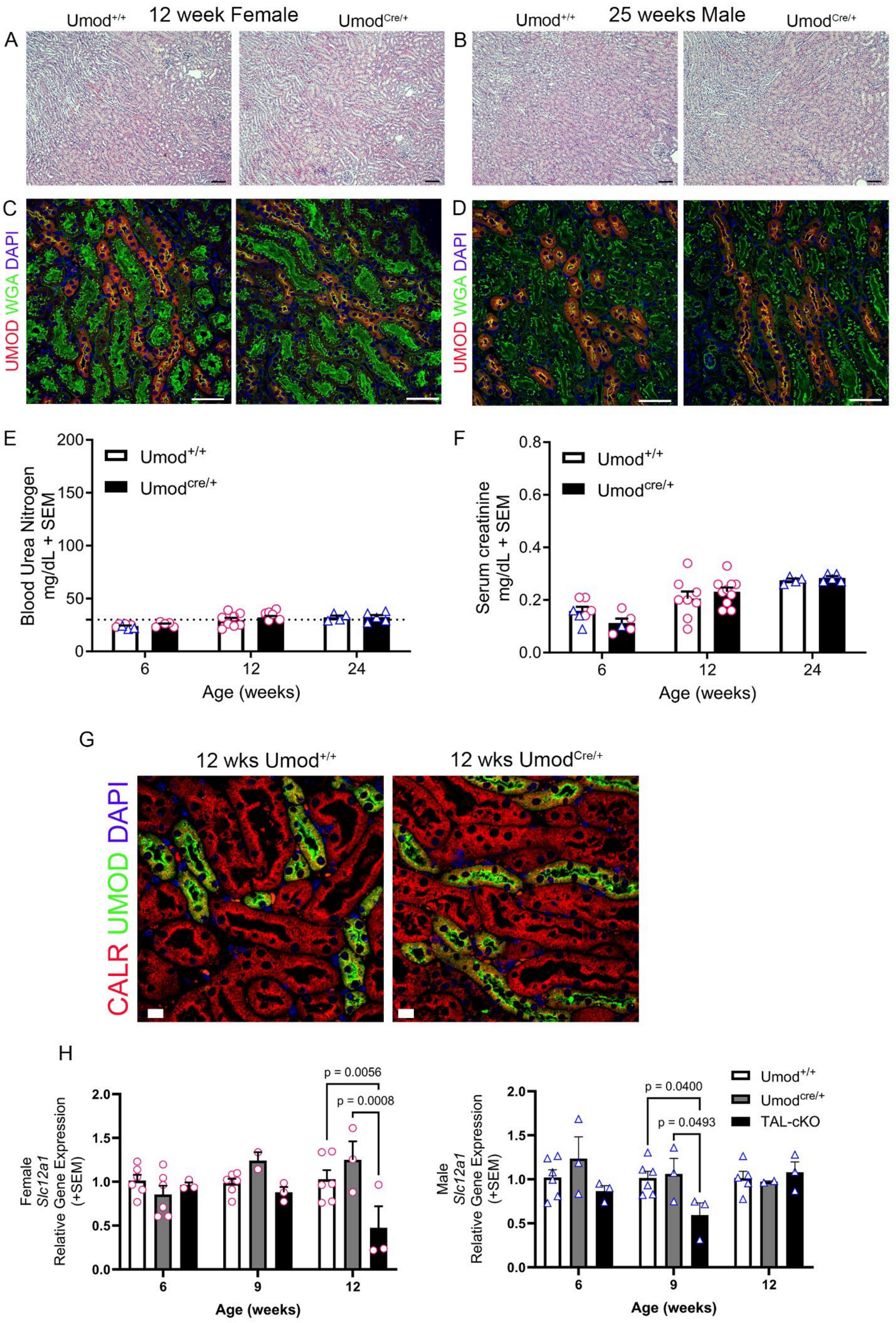

12 week Female
Umod+/+
UmodCre/+
25 weeks Male
Umod+/+
UmodCre/+
A
B
C
D
UMOD WGA DAPI
E
F
Blood Urea Nitrogen mg/dL + SEM
Umod+/+
Umodcre/+
Age (weeks)
Serum creatinine mg/dL + SEM
G
12 wks Umod+/+
12 wks UmodCre/+
CALR UMOD DAPI
H
Female Slc12a1 Relative Gene Expression (+SEM)
Male Slc12a1 Relative Gene Expression (+SEM)
p = 0.0056
p = 0.0008
p = 0.0400
p = 0.0493
Umod+/+
Umodcre/+
TAL-cKO
Age (weeks)

Figure S9. ***Umod*$^{+/\mathrm{CreERT2}}$ (*Umod*$^{\mathrm{Cre}/+}$) mice have lower uromodulin (UMOD) expression but do not develop kidney disease. *Slc12a1* (NKCC2) gene expression is not altered in *Umod*$^{+/\mathrm{CreERT2}}$.**

A,B. Histological evaluation of 12-week-old female *Umod*$^{\mathrm{Cre}/+}$ (**A**) and 25-week-old male *Umod*$^{\mathrm{Cre}/+}$ (**B**) kidneys shows no indications of disease compared to littermate controls (*Myh9*$^{f/f}$; *Myh10*$^{f/f}$; *Umod*$^{+/+}$). Scale bars = 100µm (n=3 kidneys each). **C,D.** Immunostaining for TAL marker UMOD illustrating normal localization in 12-week-old female *Umod*$^{\mathrm{Cre}/+}$ (**C**) and 25-week-old male *Umod*$^{\mathrm{Cre}/+}$ (**D**) kidneys compared to littermate controls. Scale bars = 50µm (n=3). **E.** Blood urea nitrogen measurements from female (circles) and male (triangles) *Umod*$^{\mathrm{Cre}/+}$ mice were normal and were not different from littermate controls. Normal BUN value (30mg/dL) represented as dotted line. **F.** Serum creatinine measurements from female (circles) and male (triangles) *Umod*$^{\mathrm{Cre}/+}$ mice were not different from littermate controls. **G.** Immunostaining for TAL protein UMOD and ER chaperone calreticulin (CALR) illustrating normal localization of UMOD and CALR in 12-week-old *Umod*$^{\mathrm{Cre}/+}$ kidneys compared to littermate controls (*Umod*$^{+/+}$). Scale bars = 10µm (n=3). **H.** Quantitative PCR analysis of *Slc12a1* (NKCC2) gene expression in female (circles) and male (triangles) kidneys. Significantly lower *Slc12a1* transcript level is observed at 12 weeks of age in female TAL-cKO kidneys compared to *Umod*$^{+/+}$ and *Umod*$^{\mathrm{cre}/+}$ kidneys and at 9 weeks of age in male TAL-cKO kidneys compared to *Umod*$^{+/+}$ and *Umod*$^{\mathrm{cre}/+}$ kidneys (n≥2 kidneys per genotype and age). Serum data was analyzed independently for each age using an unpaired t test with either Welch's correction or Mann-Whitney test. Transcript data was analyzed by two-way ANOVA with post hoc Tukey's multiple comparisons test.

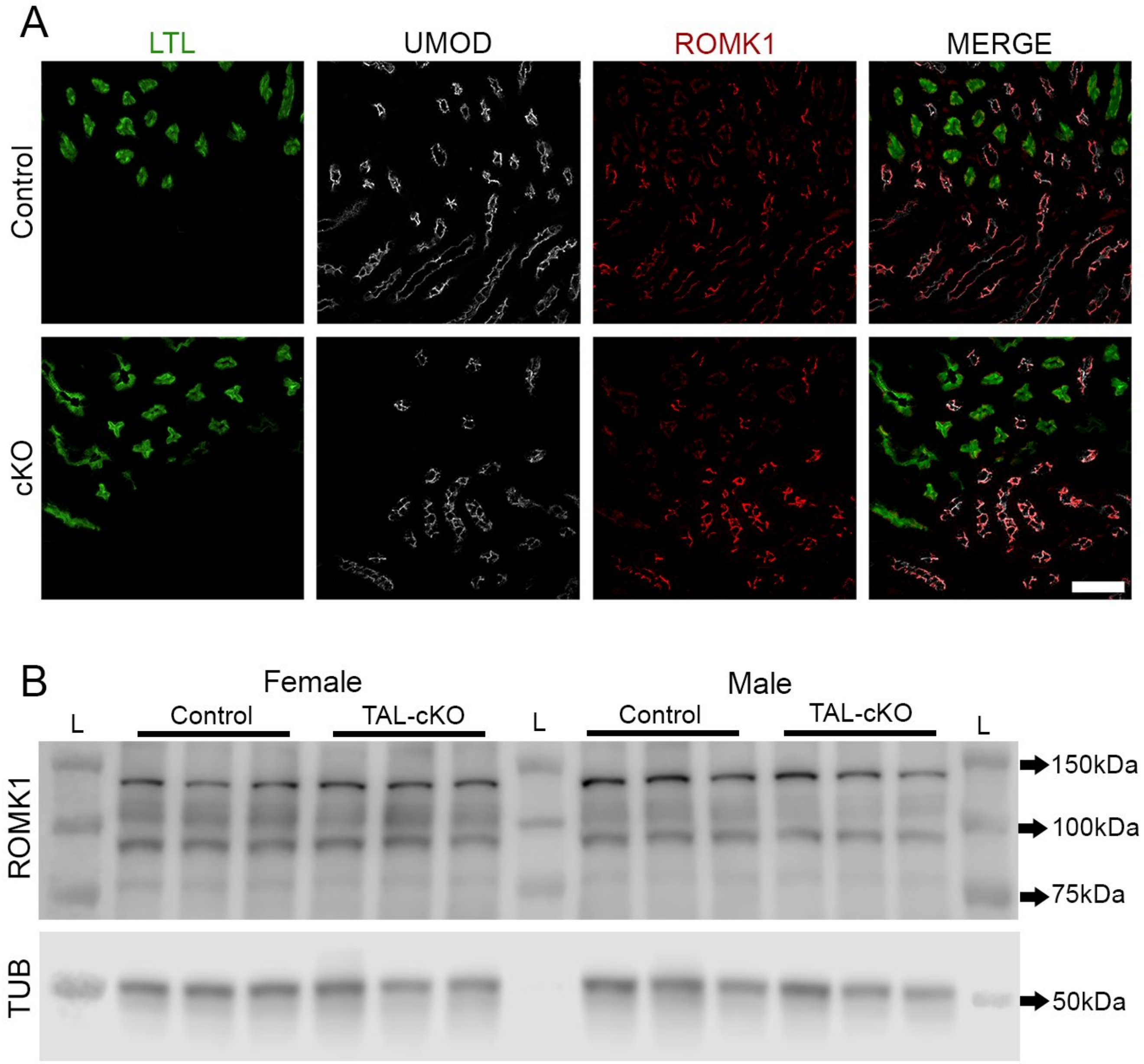

Figure S10. ROMK1 protein localizes to thick ascending limb (TAL) cell apical membrane and total protein expression is unchanged in 9-week-old *Myh9&10* TAL-cKO kidneys.

A. Fluorescence immunostaining and super resolution imaging of male and female control and *Myh9&10* TAL-cKO kidneys shows that ROMK1 localizes to the apical membrane in UMOD-positive TAL tubules. ROMK1 expression within the proximal convoluted tubule (PCT) also look unchanged in TAL-cKO kidneys. Scale bar = 50µm. **B.** Immunoblots for ROMK1 in female and male whole kidney lysates show no difference in total ROMK1 expression (~ 90kDa band) in *Myh9&10* TAL-cKO kidneys compared to controls. Tubulin (TUB) was used as the loading control. L = ladder lane. n=3.

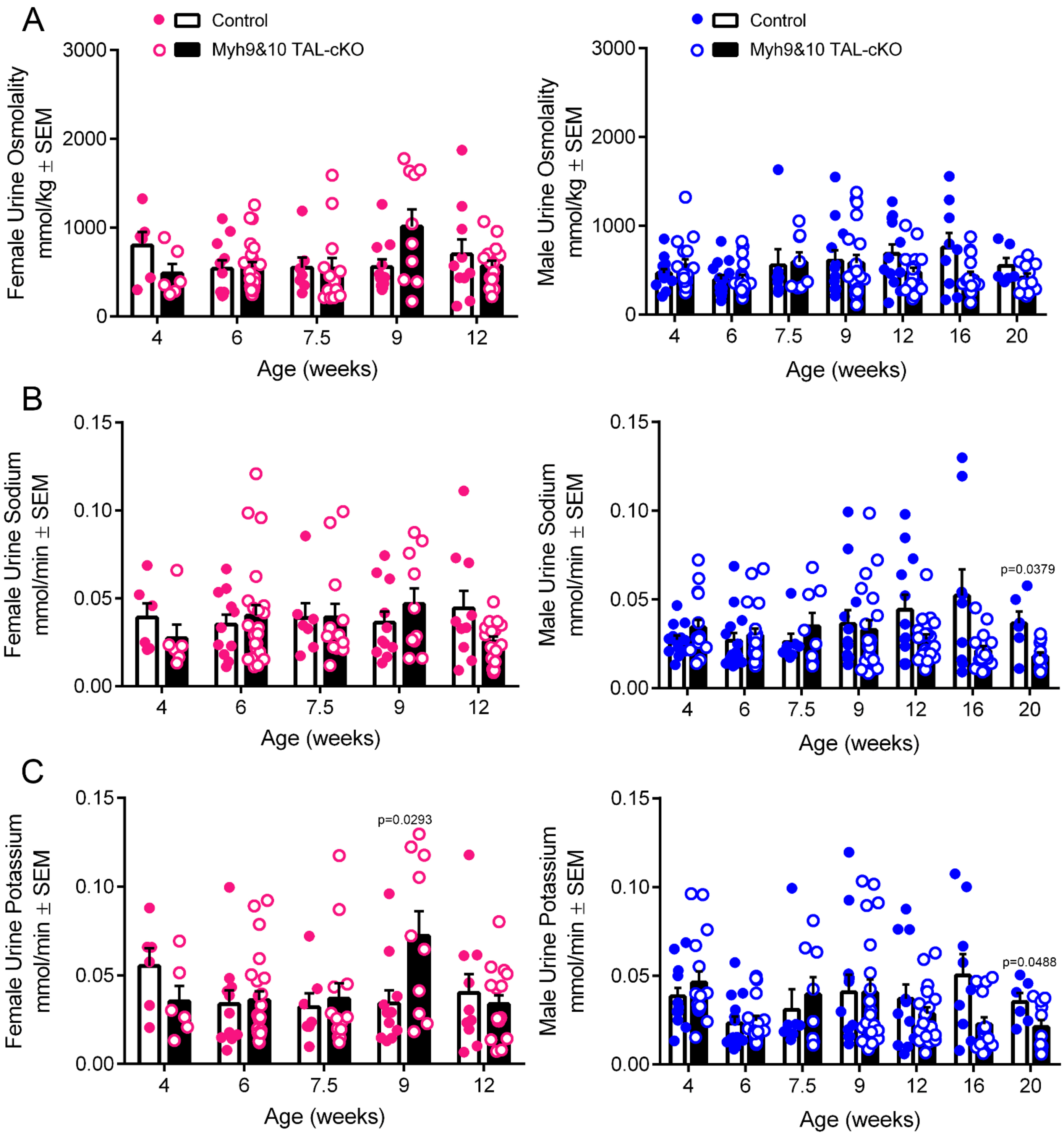

Figure S11. Urine chemistry indicates changes in electrolyte excretion in older *Myh9&10* TAL-cKO mice.

A. Urine osmolality of female (pink) and male (blue) *Myh9&10* TAL-cKO mice did not differ from controls. **B.** Urinary sodium excretion was unchanged in female *Myh9&10* TAL-cKO mice. Male *Myh9&10* TAL-cKO mice have lower sodium excretion starting at 12 weeks of age that becomes statistically significant by 20 weeks of age. **C.** Female *Myh9&10* TAL-cKO mice exhibit transiently higher potassium excretion at 9 weeks of age which returns to control levels by 12 weeks of age. Potassium excretion is lower in male *Myh9&10* TAL-cKO mice starting at 16 weeks of age and becomes statistically significant at 20 weeks of age. Sample numbers for each timepoint can be found in supplemental tables 8 and 9. Unpaired t test with Welch's correction was used to determine statistical significance between control and TAL-cKO groups for each timepoint.

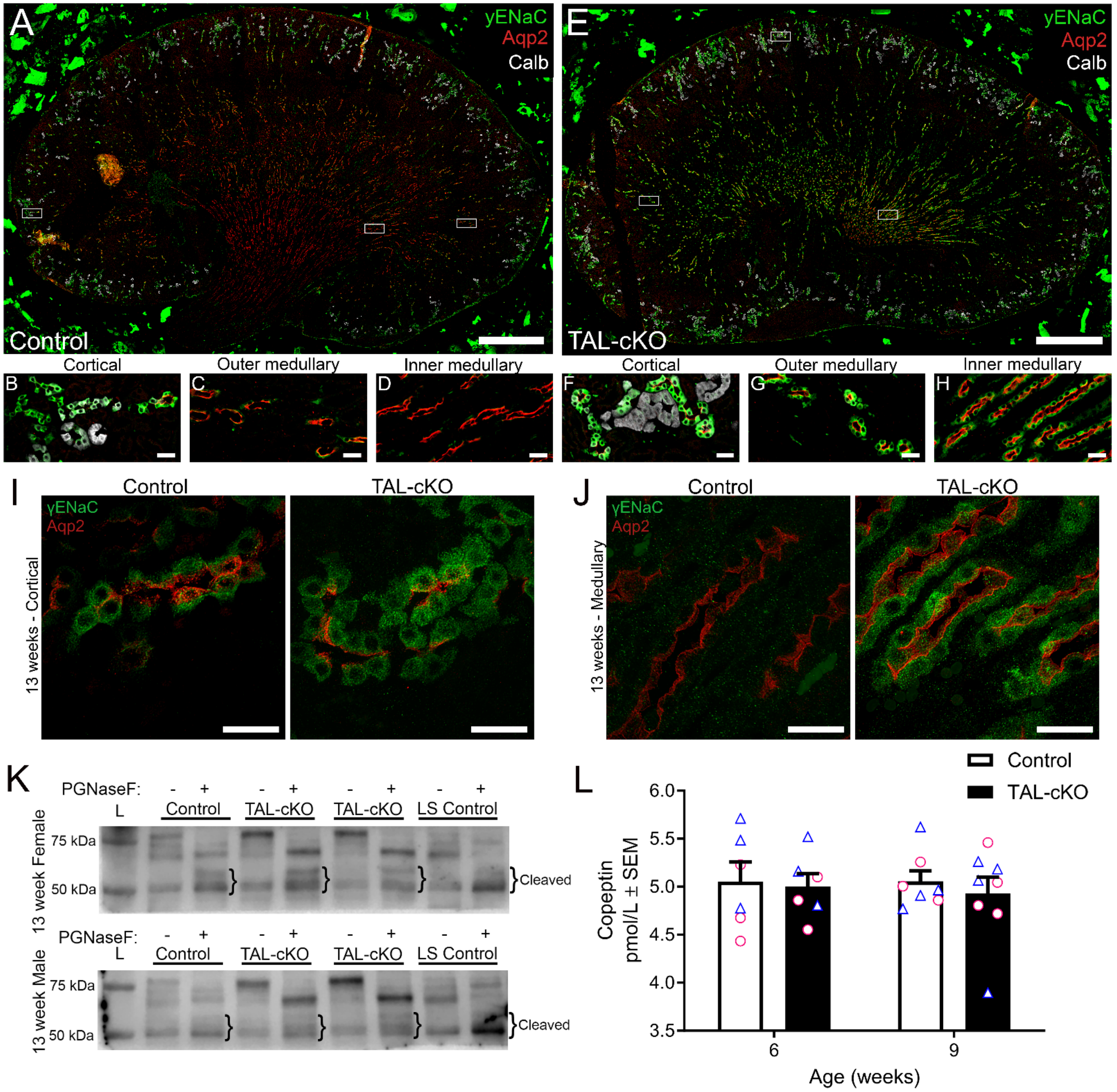

Figure S12. γEnaC expression is expanded to the medullary region in 13-week-old female *Myh9&10* TAL-cKO mice. Immunoblot of PNGaseF treated kidney lysate confirms higher γEnaC expression in *Myh9&10* TAL-cKO kidneys and copeptin levels does not change in *Myh9&10* TAL-cKO mice.

A. Fluorescence immunostaining of 13-week-old control female mouse kidney sections shows γENaC expression in the calbindin (Calb)- and aquaporin 2 (Aqp2)-positive cells of the cortex and outer medullary region of littermate control kidneys. Boxes indicate regions shown enlarged in panels B-D. **B.** In the cortex, γENaC is expressed in the distal convoluted tubule and cortical collecting ducts. **C.** Little γENaC expression is observed in the outer medullary collecting ducts. **D**. The inner medullary collecting ducts of littermate control animals do not express γENaC. **E.** Fluorescence immunostaining of 13-week-old female *Myh9&10* TAL-cKO (TAL-cKO) kidney sections show higher γENaC expression in the cortical epithelial cells and unexpected expression in the inner and outer medullary regions. Boxes indicate regions shown enlarged in panels F-H. **F.** Expression of γENaC in the

cortex of TAL-cKO kidney is higher in both distal convoluted tubules and collecting ducts. **G.** γENaC expression is observed within the outer medullary of 13-week-old female TAL-cKO mice. **H.** Inner medullary collecting ducts also express γENaC in TAL-cKO mice. Scale bars in A,E = 1000μm. Scale bars in B-D and F-H = 25μm. (n=3-4 kidneys each). **I.** Maximum intensity projections of γENaC within cortical tubules expressing Aqp2 show higher expression with loss of MYH9&10 in the TAL segment. **J.** Maximum intensity projections also show higher γENaC expression in the medullary collecting ducts (Aqp2-positive) of TAL-cKO kidneys compared to littermate controls. Scale bars in I, J = 20μm. **K.** PGNaseF treatment of whole kidney lysates clearly shows the cleavage pattern for γENaC (72 kDa=uncleaved full length, 57kDa=furin cleaved and 52kDa=distally cleaved). Although TAL-cKO kidneys have higher γENaC expression compared to littermate controls, the intensity of the cleaved bands is not different. To determine what maximum cleavage of γENaC looks like with PGNaseF treatment, kidneys from mice placed on a low salt diet were used as positive controls (LS control). **L.** Measurement of copeptin levels in the serum of littermate control and TAL-cKO male (triangles) and female (circles) mice at 6 and 9 weeks of age indicate that vasopressin secretion is not altered with loss of MYH9&10 in the TAL segment (n=6-8). Lack of significance was determined by two-way ANOVA.

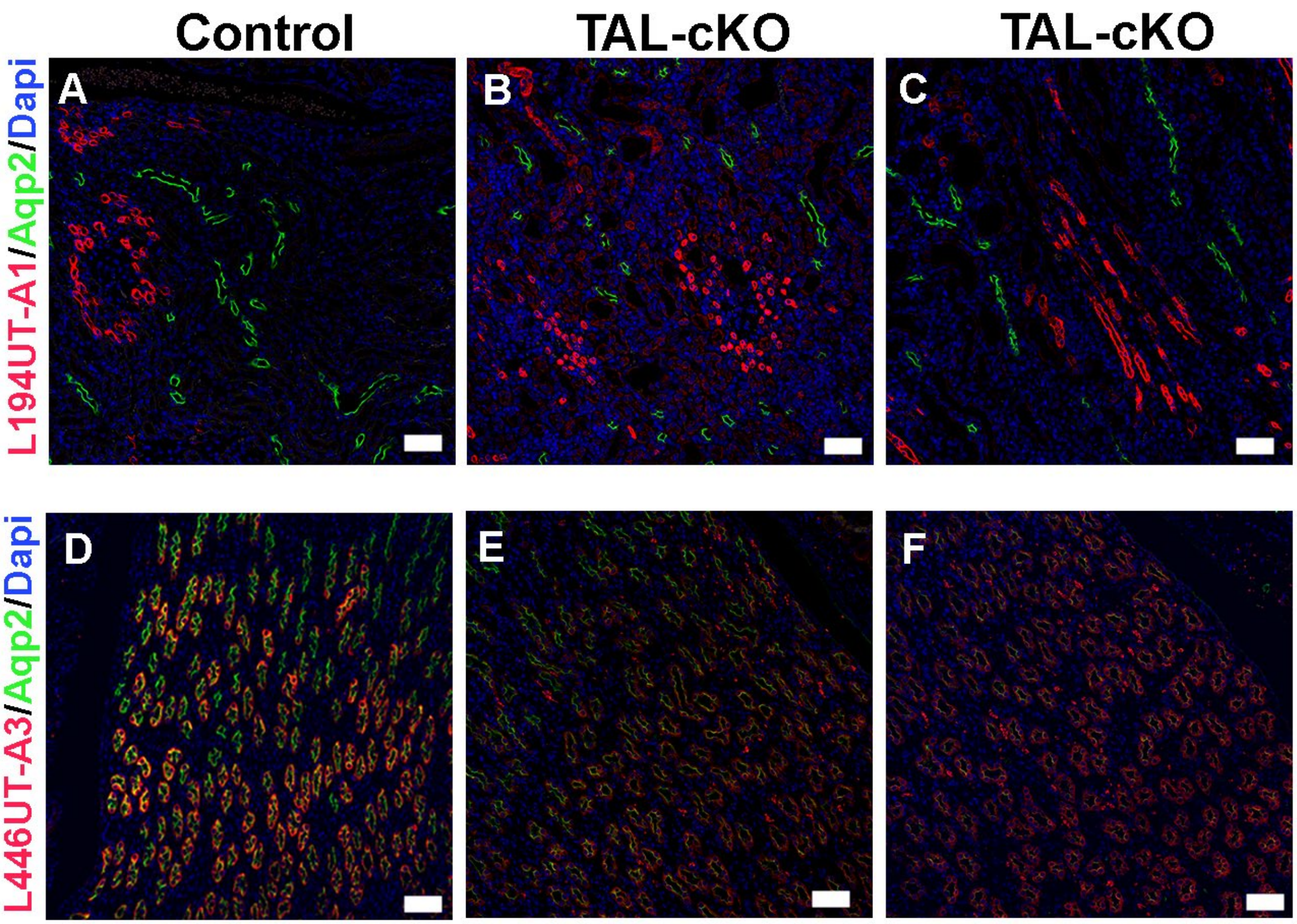

Figure S13. Expression of the urea transporter A3 isoform (UT-A3) is altered in the papillary collecting duct of *Myh9&10* TAL-cKO kidneys.

A. Immunofluorescence staining of 13 week old control and *Myh9&10* TAL-cKO kidneys for the C-terminus of UT-A shows expression of the UT-A1 in the thin limbs of the loop of Henle. **B, C.** Expression of UT-A1 in the thin limb is unchanged with loss of MYH9&10 in the TAL. **D.** Immunofluorescence staining 13 week old control and *Myh9&10* TAL-cKO kidneys with the L446 UT-A antibody shows expression of the UT-A3 isoform in the papillary collecting duct. **E, F.** Expression of the UT-A3 isoform is lower in the Myh9&10 TAL-cKO kidneys. Scale bars = 50µm. Images are representative of n=3 kidneys each.

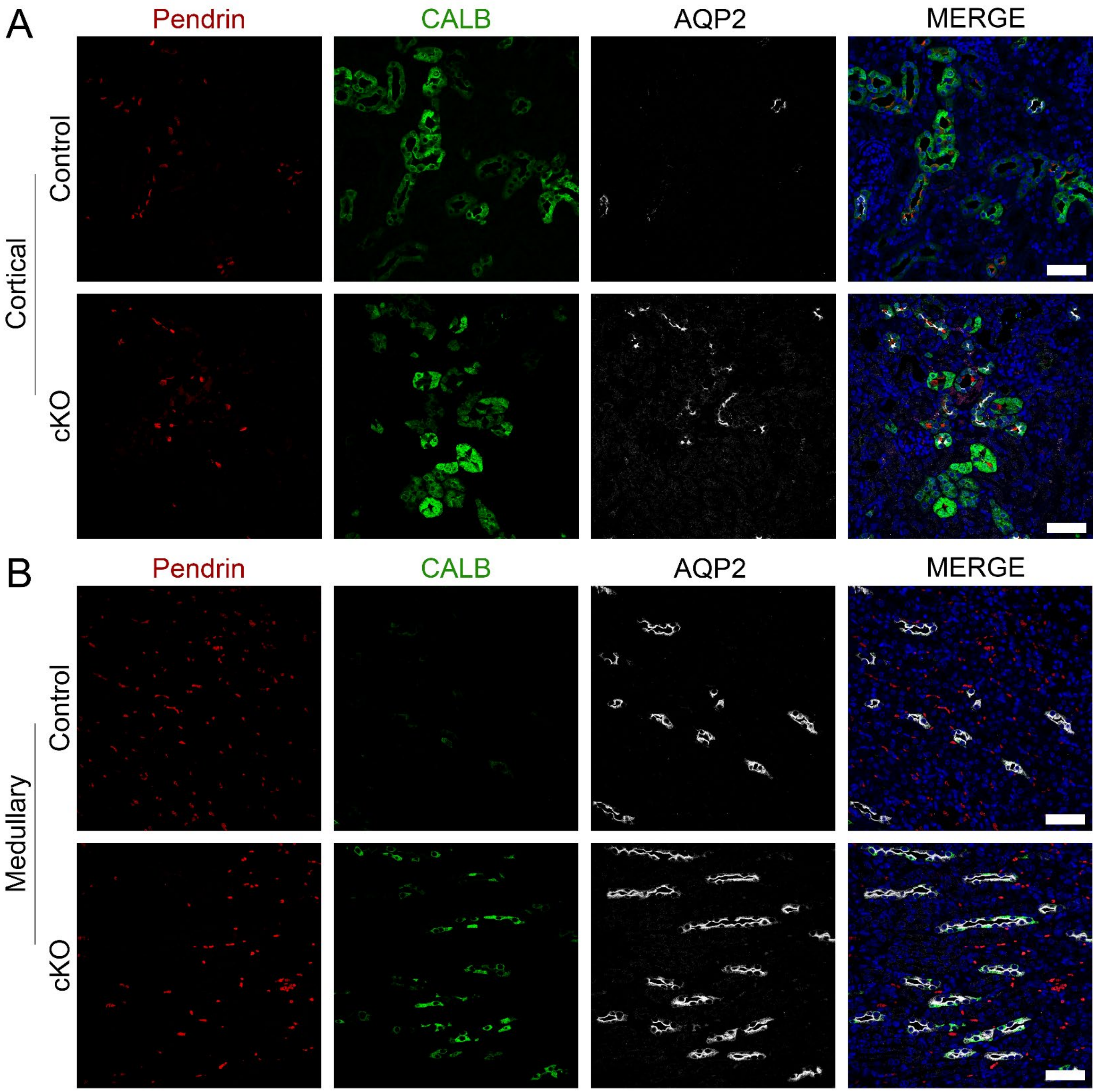

Figure S14. Male and female control and TAL-cKO kidney sections were immunostained for Pendrin, calbindin (CALB) and aquaporin-2 (AQP2) and imaged by confocal microscopy. Pendrin protein expression in the cortical (**A**) and medullary (**B**) regions of TAL-cKO kidney appears unchanged compared to control kidney at 13 weeks of age. Interstitial cells have non-specific staining in the pendrin channel on **(B)**. There was no apparent difference between male (n = 1 control, 2 TAL-cKO) and female (n = 1 control, 3 TAL-cKO) kidneys. Scale bars = 50µm.

Supplement Movie 1: Z-series images from 13-week-old control kidney section stained for reticulon-4 (RTN4, green) and uromodulin (UMOD, red) illustrating the typical subcellular distribution of each protein. RTN4 exhibits cytoplasmic distribution while UMOD is primarily enriched along the apical (luminal) region (z = 2.2µm).

Supplemental Movie 2: Z-series images from 13-week-old *Myh9&10* TAL-cKO kidney section stained for reticulon-4 (RTN4, green) and uromodulin (UMOD, red) showing gross accumulation of UMOD within TAL cells and more intense RTN4 staining than littermate control TAL cells, suggesting an ER-associated cellular phenotype (z = 3.0µm).

Supplemental Movie 3: Z- series images from 13 week old control kidney section stained for γENaC (green) within cortical tubules expressing Aqp2 (red) show expression of both proteins in the cortical collecting duct.

Supplemental Movie 4: Z- series images from 13 week old *Myh9&10* TAL-cKO kidney section stained for γENaC (green) within cortical tubules expressing Aqp2 (red) show slightly higher expression of γENaC in the cortical collecting duct.

Supplemental Movie 5: Z- series images from 13 week old control kidney section stained for γENaC (green) within medullary collecting duct tubules expressing Aqp2 (red) demonstrate no expression of γENaC (green) within the Aqp2 positive tubules as expected.

Supplemental Movie 6: Z- series images from 13 week old *Myh9&10* TAL-cKO kidney section stained for γENaC (green) within medullary collecting duct tubules expressing Aqp2 (red) demonstrate uncharacteristic expression of γENaC (green) within the Aqp2 positive tubules.

TABLE S1. Antibodies used for immunostaining and immunoblot evaluation.

Antibody	Dilution[#]	Catalog number	Company
Aquaporin 2 (AQP2)	1:200	NBP1-70378	Novus Biologicals
ATF-6α	1:2000	Sc-166659	Santa Cruz Biotechnology
Calbindin D28K (CALB)	1:1000	NBP2-50028	Novus Biologicals
Calnexin (CANX)	1:200, 1:10000	PA5-34754	Invitrogen
Calreticulin (CALR)	1:200,1:1000	Ab92516	Abcam
CD3	1:50	Ab16669	Abcam
Gapdh	1:20000	AM4300	Thermo Scientific
Hoechst 33342 (DAPI)	1:5000	#62249	Thermo Scientific
KIM1	1:1000	AF1817-SP	R&D Systems
L194 UT-A	1:200	-	Dr. Jeff Sands
L446 UT-A	1:100	-	Dr. Mark Knepper
MYH10	1:500	909901	Biolegend
MYH9	1:500	Custom Antibody	Sino Biologicals
NCC	1:200, 1:500	906-925	MRC PPU
pNCC (Thr53)	1:500	NBP2-60775	Novus Biologicals
NKCC2	2µg/mL	T9	Developmental Hybridoma
Parvalbumin	2µg/mL	SMC-563C	StressMarq
ROMK1	1:200	APC-001	Alamone
RTN4	1:500	AHP1799	BioRad
TFRC	1:1000	Ab84036	Abcam
UMOD	1:200	PA5-47706	Thermo Scientific
Wheat germ agglutinin (WGA) Oregon Green 488	1:200	W6748	Invitrogen
XBP1	1:500	Ab85546	Abcam
α-Tubulin	1:10000	2144S	Cell Signaling Technology
β-Tubulin	1:5000	MA5-16308	Thermo Scientific
γ ENaC	1:100, 1:500	SPC-405	StressMarq

[#]First listed dilution is for immunostaining and the second is for immunoblotting if antibody dilution varied between methodologies.

TABLE S2. Primers used for gene expression analysis.

Gene	Forward Primer Sequence	Reverse Primer Sequence
Gapdh	TTCAACAGCAACTCCCACTCTTCCACCTTCGATG	TCCACCACCCTGTTGCTGTAGCCGTATTCATTGTC
Slc12a1	CTGGAAAGTCTTTGAAGAGATGATTG	GAGGTTGGCGGCTCTGGA
Slc12a3	TCCCTTCGGCAGGTGAGACTGAGTGAGATACTGC	AGGTGAGCACATTCTCCTGGTTGCCTCGGATGAG
Umod	GTTCAGGCATCAGAAACACAAGG	GTGAACCAGGAGGTTACCATCATTA
Scnn1g	AAAGCTCAACAAGACTGACCTGGCCAAGCTCTTG	TCCACAGGCCGAGCTGTCCACCAAAGTTAGACAG
Elf5	AGTGGTTAAGTCAGAAGCCCTGGCAAAGATGTGG	AAATTTGTACACTAACCTCCGGTCAACCCGCTCC
Akr1b1	ACGAGGATGAGGATGAGATCGCACAAGTCACTAA	AATAGAGTGGCCACTCTTTCTGTAGATGTCATGT
Ranbp3l	TGTGGTGCACGTTCCATGACAAGAGCATGGTGAA	AAATCGGTGTCACTAGGTATCACGTTCCCTGAGG

TABLE S3. Metabolic measurements for female UMOD$^{+/+}$ and UMOD$^{cre/+}$ mice.

	6 Weeks			12 weeks		
	Umod$^{+/+}$ n =6	Umod$^{Cre/+}$ n = 5	Sig	Umod$^{+/+}$ n = 6	Umod$^{Cre/+}$ n = 5	Sig
**Weight g ± SEM**	18.10 ± 0.22	18.40 ± 1.09	0.7999	19.07 ± 0.29	19.54 ± 1.09	0.6937
**Water drank mL/g ± SEM**	0.45 ± 0.06	0.41 ± 0.06	0.6211	0.46 ± 0.07	0.43 ± 0.05	0.6938
**Urine produced mL/g ± SEM**	0.12 ± 0.02	0.11 ± 0.02	0.7537	0.13 ± 0.04	0.06 ± 0.02	0.1957
**Urine pH ± SEM**	6.08 ± 0.24	6.13 ± 0.05	0.8625	6.07 ± 0.10	6.06 ± 0.05	0.9123
**Osmolality mmol/kg ± SEM**	553.2 ± 119.3	535.8 ± 79.9	0.9066	393.7 ± 64.4	1107.0 ± 359.7	0.1183
**Total urinary protein mmol/min ± SEM**	0.0005 ± 0.0001	0.0008 ± 0.0002	0.2290	0.0005 ± 0.0001	0.0015 ± 0.0004	0.0575
**Sodium urinary levels mmol/min ± SEM**	0.05 ± 0.01	0.05 ± 0.01	0.8942	0.04 ± 0.01	0.09 ± 0.04	0.2211
**Potassium urinary levels mmol/min ± SEM**	0.039 ± 0.008	0.038 ± 0.006	0.9150	0.027 ± 0.004	0.067 ± 0.022	0.1459
**Glucose urinary levels mmol/min ± SEM**	0.0002 ± 0.0001	0.0003 ± 0.0001	0.5225	0.0001 ± 0.00004	0.0006 ± 0.0003	0.1868

TABLE S4. Metabolic measurements for male UMOD$^{+/+}$ and UMOD$^{cre/+}$ mice.

	6 Weeks			12 weeks			16 weeks			24 weeks		
	Umod$^{+/+}$ n =5	Umod$^{Cre/+}$ n = 6	Sig	Umod$^{+/+}$ n = 6	Umod$^{Cre/+}$ n = 5	Sig	Umod$^{+/+}$ n = 6	Umod$^{Cre/+}$ n = 6	Sig	Umod$^{+/+}$ n = 6	Umod$^{Cre/+}$ n = 6	Sig
**Weight g ± SEM**	20.36 ± 0.41	21.02 ± 0.38	0.2671	23.25 ± 0.33	22.64 ± 0.51	0.3486	25.02 ± 0.85	25.03 ± 0.52	0.9871	29.43 ± 1.46	28.67 ± 0.77	0.6564
**Water drank mL/g ± SEM**	0.51 ± 0.05	0.45 ± 0.08	0.5646	0.52 ± 0.03	0.37 ± 0.07	0.0898	0.46 ± 0.06	0.37 ± 0.07	0.3699	0.41 ± 0.06	0.29 ± 0.03	0.1160
**Urine produced mL/g ± SEM**	0.15 ± 0.06	0.18 ± 0.06	0.7675	0.18 ± 0.06	0.08 ± 0.03	0.1712	0.14 ± 0.04	0.09 ± 0.02	0.2321	0.18 ± 0.04	0.11 ± 0.01	0.1555
**Urine pH ± SEM**	6.38 ± 0.12	6.32 ± 0.06	0.6665	6.25 ± 0.07	6.26 ± 0.07	0.8846	6.23 ± 0.05	6.25 ± 0.11	0.8850	6.17 ± 0.03	6.21 ± 0.03	0.2852
**Osmolality mmol/kg ± SEM**	555.2 ± 98.2	578.2 ± 103.0	0.8754	453.2 ± 138.2	719.6 ± 177.4	0.2702	466.8 ± 113.2	505.7 ± 163.9	0.8498	313.0 ± 70.8	386.7 ± 30.0	0.3711
**Total urinary protein mmol/min ± SEM**	0.0006 ± 0.0010	0.0077 ± 0.0009	0.3055	0.0019 ± 0.0006	0.0037 ± 0.0012	0.2308	0.0023 ± 0.0008	0.0031 ± 0.0012	0.6039	0.0015 ± 0.0004	0.0025 ± 0.0003	0.0772
**Sodium urinary levels mmol/min ± SEM**	0.051 ± 0.011	0.047 ± 0.012	0.8169	0.039 ± 0.016	0.064 ± 0.015	0.2746	0.038 ± 0.009	0.051 ± 0.016	0.4962	0.021 ± 0.002	0.029 ± 0.003	0.0587
**Potassium urinary levels mmol/min ± SEM**	0.045 ± 0.01	0.043 ± 0.01	0.9037	0.029 ± 0.01	0.050 ± 0.01	0.1877	0.034 ± 0.01	0.041 ± 0.01	0.5655	0.021 ± 0.003	0.029 ± 0.002	0.0653
**Glucose mmol/min ± SEM**	0.0002 ± 0.0001	0.0002 ± 0.0001	0.5733	0.0002 ± 0.0001	0.0003 ± 0.0001	0.4773	0.0002 ± 0.0001	0.0003 ± 0.0001	0.5433	0.0001 ± 0.00004	0.0001 ± 0.00004	0.6624

TABLE S5. Serum measurements of $UMOD^{+/+}$ and $UMOD^{cre/+}$ mice.

	12 Weeks Female			24 Weeks Male		
	**$Umod^{+/+}$ n = 8**	**$Umod^{Cre/+}$ n = 10**	**Sig**	**$Umod^{+/+}$ n = 4**	**$Umod^{Cre/+}$ n = 5**	**Sig**
**Sodium mmol ± SEM**	148.6 ± 1.4	150.4 ± 1.0	0.3222	149.5 ± 0.6	151.8 ± 0.7	0.0510
**Potassium mmol ± SEM**	4.68 ± 0.11	4.20 ± 0.12	**0.0106**	4.65 ± 0.10	4.76 ± 0.09	0.4252
**Calcium mg/dL ± SEM**	9.33 ± 0.18	10.06 ± 0.39	0.1121	10.00 ± 0.21	10.58 ± 0.33	0.1853
**Glucose mg/dL ± SEM**	273.1 ± 16.6	300.8 ± 14.3	0.2252	349.3 ± 9.7	330.2 ± 6.5	0.1584
**Urea nitrogen mg/dL ± SEM**	29.5 ± 2.3	32.1 ± 1.5	0.3540	32.3 ± 1.7	32.4 ± 2.1	0.9563
**Creatinine mg/dL ± SEM**	0.21 ± 0.03	0.23 ± 0.02	0.4276	0.28 ± 0.01	0.28 ± 0.01	0.3687
**Albumin g/dL ± SEM**	2.96 ± 0.16	3.12 ± 0.08	0.4094	3.25 ± 0.03	3.54 ± 0.12	0.0664

TABLE S6: Serum measurements of female control and *Myh9&10* TAL-cKO mice.

	6 Weeks			9 Weeks			12 weeks		
	**Control n = 8**	**Myh9&10 TAL-cKO n = 10**	**Sig**	**Control n =18**	**Myh9&10 TAL-cKO n =13**	**Sig**	**Control n = 8**	**Myh9&10 TAL-cKO n = 9**	**Sig**
**Calcium mg/dL ± SEM**	8.46 ± 0.56	8.52 ± 0.38	0.9339	9.53 ± 0.32	9.12 ± 0.23	0.3031	8.15 ± 0.29	9.54 ± 0.44	**0.0194**
**Glucose mg/dL ± SEM**	222.4 ± 7.6	225.4 ± 7.9	0.7866	235.3 ± 7.4	259.7 ± 8.5	**0.0395**	217.1 ± 12.5	183.9 ± 11.0	0.0641
**Urea nitrogen mg/dL ± SEM**	25.6 ± 2.0	22.7 ± 1.2	0.2305	27.2 ± 1.1	46.5 ± 4.4	**<0.0001**	27.5 ± 0.9	126.6 ± 9.1	**<0.0001**
**Creatinine mg/dL ± SEM**	0.10 ± 0.02	0.12 ± 0.02	0.4620	0.14 ± 0.01	0.19 ± 0.01	**0.0035**	0.11 ± 0.02	0.25 ± 0.01	**<0.0001**
**Albumin g/dL ± SEM**	2.74 ± 0.09	2.66 ± 0.04	0.4565	2.82 ± 0.06	3.04 ± 0.05	**0.0079**	2.60 ± 0.05	3.02 ± 0.07	**0.0003**

TABLE S7: Serum measurements of male control and *Myh9&10* TAL-cKO mice.

	6 Weeks			9 Weeks			12 Weeks			20 Weeks		
	Control n = 7	Myh9&10 TAL-cKO n = 8	Sig	Control n =15	Myh9&10 TAL-cKO n =22	Sig	Control n = 6	Myh9&10 TAL-cKO n = 9	Sig	Control n = 9	Myh9&10 TAL-cKO n = 7	Sig
**Calcium mmol ± SEM**	9.54 ± 0.30	9.01 ± 0.21	0.1744	9.45 ± 0.20	9.33 ± 0.08	0.5663	8.55 ± 0.37	10.23 ± 0.49	**0.0174**	9.50 ± 0.29	10.81 ± 0.58	0.0719
**Glucose mg/dL ± SEM**	232.3 ± 6.3	249.0 ± 12.4	0.2574	220.3 ± 9.3	236.0 ± 5.4	0.1601	198.2 ± 10.3	218.7 ± 21.2	0.4021	200.0 ± 13.9	189.7 ± 17.4	0.6524
**Urea nitrogen mg/dL ± SEM**	23.14 ± 0.91	23.25 ± 1.01	0.9385	29.53 ± 1.88	43.50 ± 4.49	**0.0078**	23.67 ± 1.73	103.20 ± 6.52	**<0.0001**	25.89 ± 1.11	149.40 ± 6.74	**<0.0001**
**Creatinine mg/dL ± SEM**	0.10 ± 0.02	0.10 ± 0.01	0.7318	0.15 ± 0.01	0.17 ± 0.01	0.0608	0.10 ± 0.01	0.27 ± 0.02	**<0.0001**	0.14 ± 0.01	0.51 ± 0.04	**<0.0001**
**Albumin g/dL ± SEM**	2.86 ± 0.13	2.75 ± 0.07	0.4931	3.01 ± 0.08	2.99 ± 0.04	0.7692	2.73 ± 0.10	3.30 ± 0.18	**0.0175**	3.09 ± 0.07	3.21 ± 0.13	0.4055

TABLE S8. Metabolic measurements for control and *MYH9&10* TAL-cKO female mice.

	6 Weeks			7.5 weeks			9 weeks			12 weeks		
	**Control n =11**	***Myh9&10* TAL-cKO n = 23**	**Sig**	**Control n = 7**	***Myh9&10* TAL-cKO n = 13**	**Sig**	**Control n = 11**	***Myh9&10* TAL-cKO n = 10**	**Sig**	**Control n = 10**	***Myh9&10* TAL-cKO n = 16**	**Sig**
**Weight g ± SEM**	16.41 ± 0.44	16.36 ± 0.26	0.9238	17.91 ± 0.35	17.04 ± 0.34	0.0903	18.00 ± 0.36	17.24 ± 0.48	0.2225	19.57 ± 0.37	16.27 ± 0.34	**<0.0001**
**Water drank mL/g ± SEM**	0.48 ± 0.05	0.41 ± 0.04	0.2789	0.41 ± 0.07	0.49 ± 0.03	0.3626	0.42 ± 0.05	0.33 ± 0.06	0.2470	0.36 ± 0.08	0.41 ± 0.04	0.5761
**Urine produced mL/g ± SEM**	0.13 ± 0.03	0.10 ± 0.01	0.3484	0.12 ± 0.003	0.10 ± 0.02	0.7035	0.10 ± 0.02	0.05 ± 0.02	0.0629	0.10 ± 0.03	0.10 ± 0.02	0.9008
**Urine pH ± SEM**	6.20 ± 0.10	6.29 ± 0.05	0.4873	5.99 ±0.08	6.26 ± 0.08	**0.0314**	6.14 ± 0.09	6.02 ± 0.10	0.4089	5.92 ± 0.05	5.38 ± 0.04	**<0.0001**
**Osmolality mmol/kg ± SEM**	536.3 ± 95.2	550.1 ± 62.2	0.9046	549.1 ± 115.8	539.0 ± 120.3	0.9523	557.2 ± 87.7	1012.0 ± 193.6	0.0524	698.1 ± 169.1	568.7 ± 58.5	0.4845
**Total urinary protein mmol/min ± SEM**	0.0005 ± 0.0001	0.0007 ± 0.0001	0.2682	0.0004 ± 0.0001	0.0009 ± 0.0004	0.2962	0.0006 ± 0.0002	0.0028 ± 0.0008	**0.0244**	0.0009 ± 0.0004	0.0011 ± 0.0002	0.6464
**Sodium urinary levels mmol/min ± SEM**	0.035 ± 0.006	0.040 ± 0.006	0.5593	0.039 ± 0.008	0.039 ± 0.007	0.9709	0.036 ± 0.006	0.047 ± 0.009	0.3483	0.044 ± 0.010	0.025 ± 0.003	0.0944
**Potassium urinary levels mmol/min ± SEM**	0.034 ± 0.008	0.036 ± 0.005	0.8165	0.032 ± 0.008	0.037 ± 0.009	0.6843	0.034 ± 0.008	0.072 ± 0.014	**0.0293**	0.040 ± 0.011	0.034 ± 0.005	0.5902
**Glucose mmol/min ± SEM**	0.0002 ± 0.0001	0.0002 ± 0.00004	0.7489	0.0002 ± 0.0001	0.0003 ± 0.0001	0.6314	0.0002 ± 0.0001	0.0009 ± 0.0002	**0.0132**	0.0004 ± 0.0001	0.0005 ± 0.0001	0.4236

TABLE S9. Metabolic measurements for control and *MYH9&10* TAL-cKO male mice.

	6 weeks			7.5 weeks			9 weeks			12 weeks			16 weeks			20 weeks		
	**Control n = 14**	**Myh9&10 TAL-cKO n = 19**	**Sig**	**Control n = 7**	**Myh9&10 TAL-cKO n = 8**	**Sig**	**Control n = 12**	**Myh9&10 TAL-cKO n = 20**	**Sig**	**Control n = 12**	**Myh9&10 TAL-cKO n = 19**	**Sig**	**Control n = 9**	**Myh9&10 TAL-cKO n = 15**	**Sig**	**Control n = 6**	**Myh9&10 TAL-cKO n = 11**	**Sig**
**Weight g ± SEM**	20.65 ± 0.41	20.09 ± 0.47	0.3274	21.86 ± 0.80	20.64 ± 0.64	0.2542	23.50 ± 0.66	21.59 ± 0.49	**0.0301**	24.26 ± 0.78	19.87 ± 0.43	**0.0001**	25.56 ± 1.27	20.14 ± 0.64	**0.0024**	29.96 ± 1.49	21.54 ± 1.06	**0.0010**
**Water drank mL/g ± SEM**	0.43 ± 0.05	0.37 ± 0.03	0.2231	0.38 ± 0.04	0.31 ± 0.04	0.2412	0.30 ± 0.04	0.30 ± 0.04	0.9443	0.29 ± 0.03	0.42 ± 0.04	**0.0177**	0.31 ± 0.04	0.46 ± 0.04	**0.0074**	0.23 ± 0.05	0.44 ± 0.05	**0.0118**
**Urine produced mL/g ± SEM**	0.20 ± 0.03	0.18 ± 0.02	0.5608	0.18 ± 0.03	0.13 ± 0.04	0.4024	0.12 ± 0.03	0.11 ± 0.02	0.6910	0.10 ± 0.02	0.13 ± 0.02	0.1863	0.08 ± 0.02	0.17 ± 0.02	**0.0122**	0.08 ± 0.02	0.17 ± 0.03	**0.0330**
**Urine pH ± SEM**	6.39 ± 0.07	6.35 ± 0.04	0.6178	6.15 ± 0.14	6.34 ± 0.05	0.2153	6.19 ± 0.06	5.99 ± 0.05	**0.0122**	6.30 ± 0.07	5.67 ± 0.05	**<0.0001**	6.19 ± 0.09	5.63 ± 0.06	**0.0001**	6.07 ± 0.11	5.54 ± 0.08	**0.0027**
**Osmolality mmol/kg ± SEM**	394.9 ± 50.5	408.1 ± 41.6	0.8425	553.7 ± 182.4	592.1 ± 109.6	0.8604	608.2 ± 117.9	581.6 ± 89.2	0.8587	682.8 ± 108.5	477.5 ± 50.1	0.1053	755.3 ± 162.2	430.6 ± 52.4	0.0868	549.3 ± 88.5	415.3 ± 49.5	0.2217
**Total urinary protein mmol/min ± SEM**	0.0018 ± 0.0005	0.0015 ± 0.0003	0.6800	0.0019 ± 0.0007	0.0025 ± 0.0006	0.5204	0.0042 ± 0.0001	0.0032 ± 0.0008	0.5179	0.0031 ± 0.0006	0.0022 ± 0.0004	0.2767	0.0032 ± 0.0001	0.0013 ± 0.0004	0.1159	0.0020 ± 0.0002	0.0008 ± 0.0003	**0.0074**
**Sodium urinary levels mmol/min ± SEM**	0.027 ± 0.004	0.030 ± 0.004	0.5912	0.026 ± 0.005	0.035 ± 0.007	0.3380	0.036 ± 0.008	0.033 ± 0.005	0.7251	0.044 ± 0.009	0.027 ± 0.003	0.0874	0.052 ± 0.015	0.021 ± 0.003	0.0717	0.036 ± 0.007	0.018 ± 0.002	**0.0379**
**Potassium urinary levels mmol/min ± SEM**	0.023 ± 0.004	0.024 ± 0.003	0.7969	0.031 ± 0.011	0.039 ± 0.010	0.5802	0.041 ± 0.010	0.041 ± 0.007	0.9926	0.037 ± 0.008	0.028 ± 0.004	0.3612	0.050 ± 0.012	0.023 ± 0.004	0.0547	0.035 ± 0.005	0.021 ± 0.004	**0.0488**
**Glucose mmol/min ± SEM**	0.0001 ± 0.00004	0.0001 ± 0.00002	0.4194	0.0002 ± 0.0001	0.0002 ± 0.0001	0.9986	0.0002 ± 0.0001	0.0004 ± 0.0001	0.1865	0.0003 ± 0.0001	0.0003 ± 0.0001	0.4417	0.0003 ± 0.0001	0.0001 ± 0.00003	**0.0404**	0.0002 ± 0.0001	0.0001 ± 0.00002	0.1473
