# Supplementary material for: Thick Ascending Limb Specific Inactivation of Myh9 and Myh10 Myosin Motors Results in Progressive Kidney Disease and Drives Sex-specific Cellular Adaptation in the Distal Nephron and Collecting Duct
Source: Function (Oxf). 2024 Nov 5;6(1):zqae048. doi: 10.1093/function/zqae048 (PMC11815580; doi:10.1093/function/zqae048)

UNCUT Western Blot Images

UMOD Blots

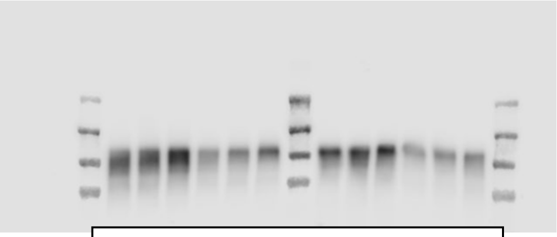

6 week UMOD Blot gel 1

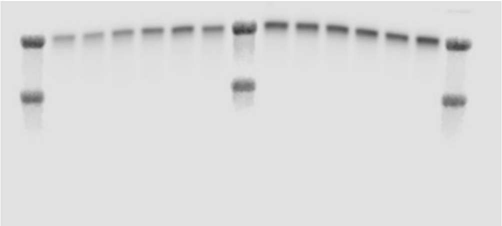

6 week tubulin band for UMOD Blot gel 1

6 week UMOD Blot gel 2

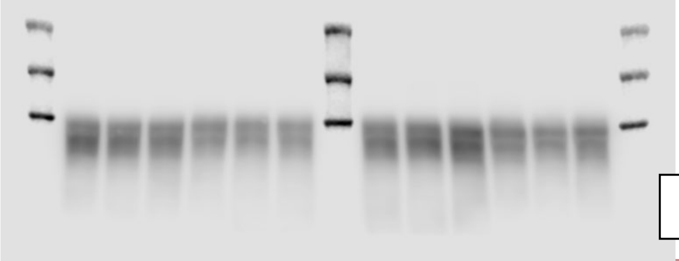

Ponceau for 6 week UMOD Blot gel 2

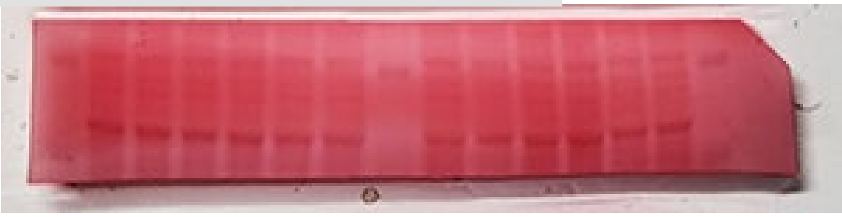

9 week UMOD Blot gel 1

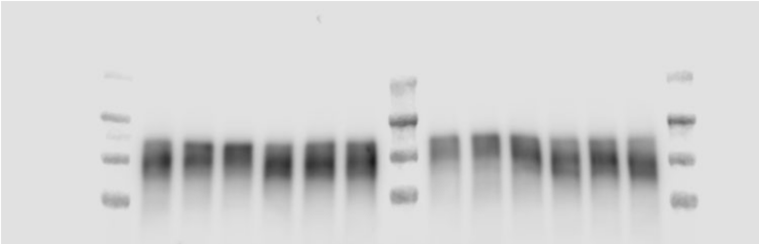

9 week UMOD Blot gel 2

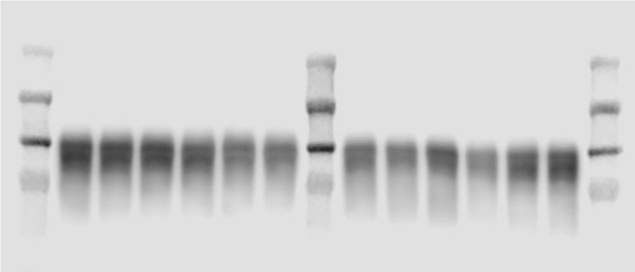

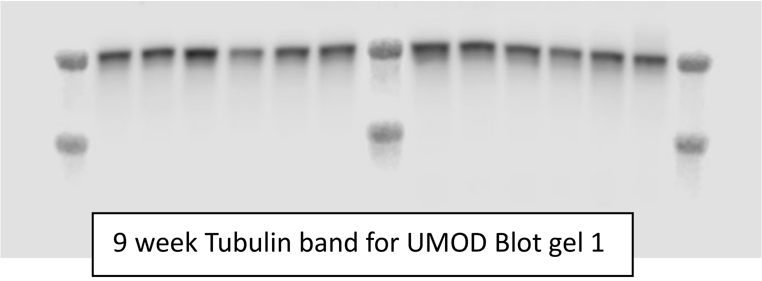

9 week Tubulin band for UMOD Blot gel 1

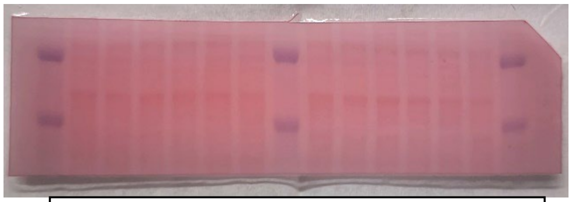

9 week ponceau gel 2

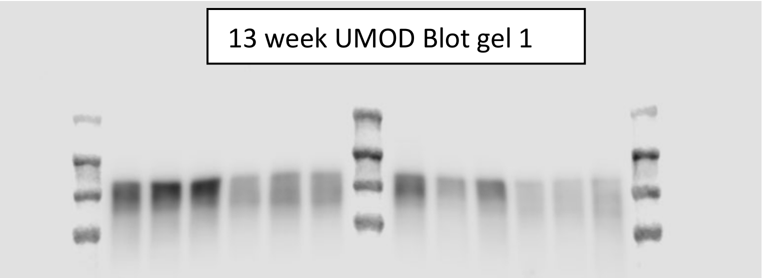

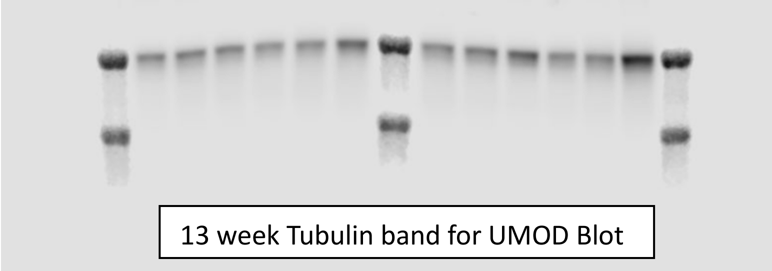

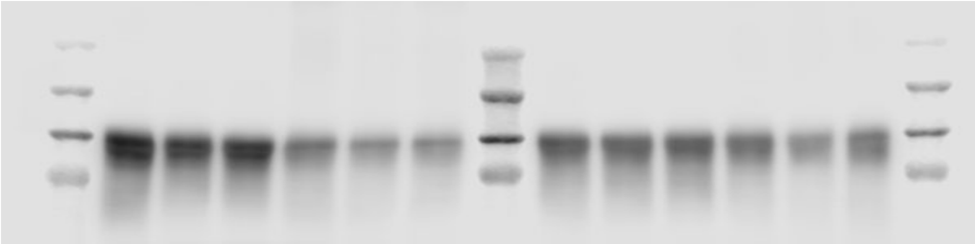

13 week UMOD Blot gel 2

13 week GAPDH for UMOD blot gel 2

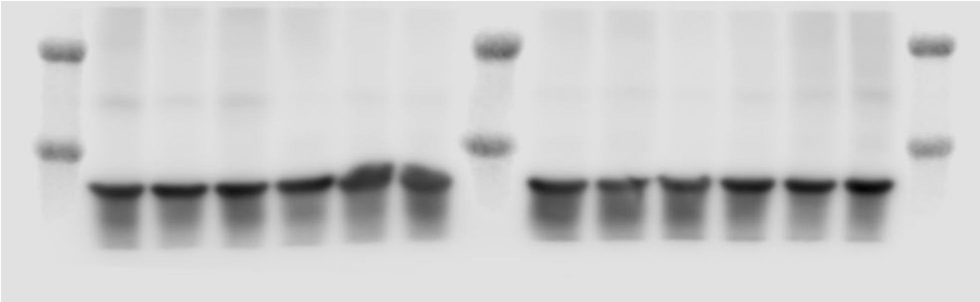

Male NKCC2 Blots

6 week male NKCC2 blot gel 1

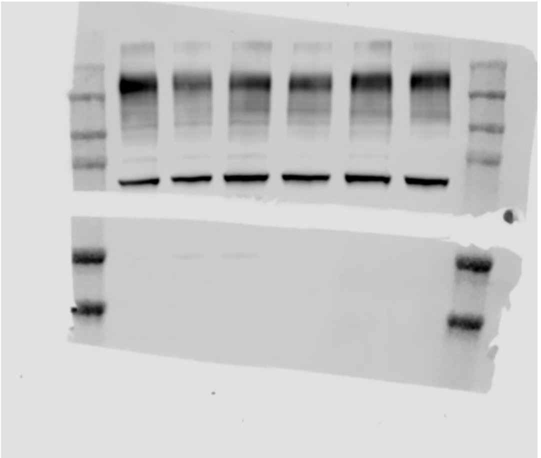

6 week male Tubulin band for NKCC2 blot gel 1

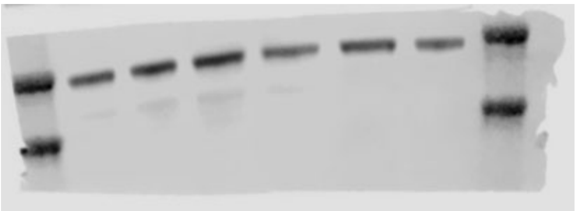

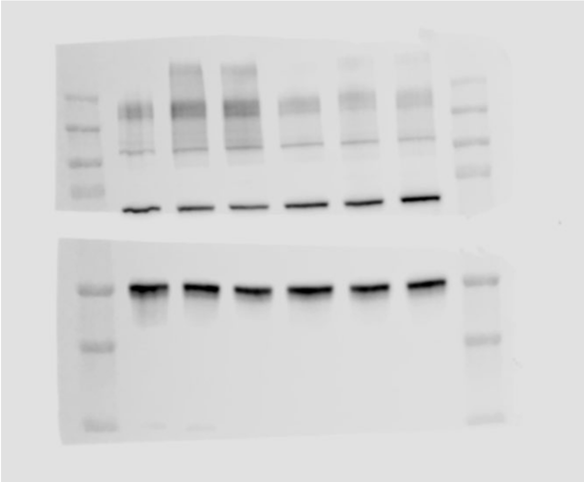

9 week male NKCC2 blot gel 1 with tubulin loading control

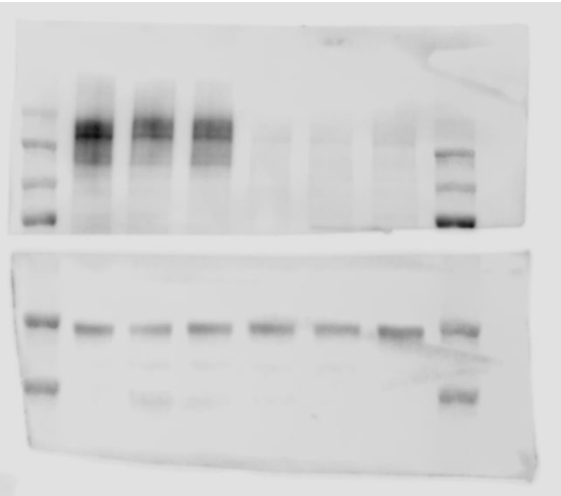

13 week male NKCC2 blot gel 1 with tubulin loading control

6 week male NKCC2 blot gel 2

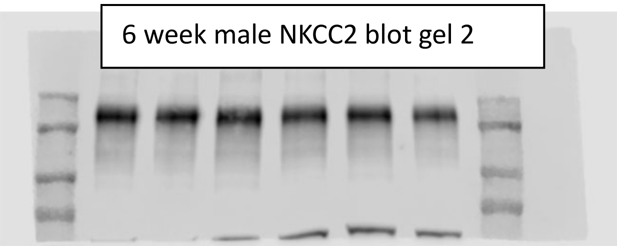

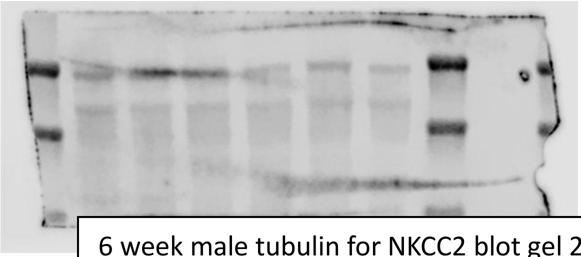

6 week male tubulin for NKCC2 blot gel 2

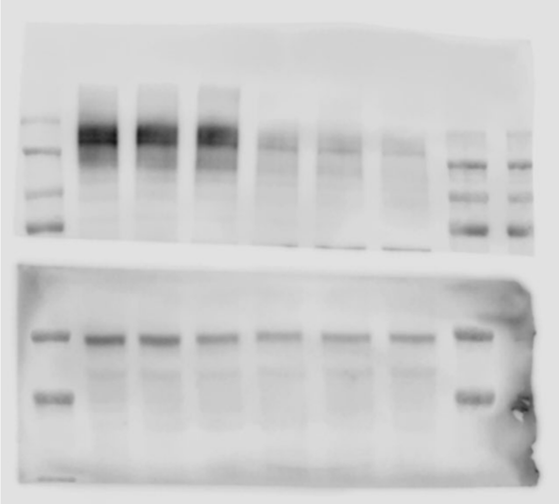

9 week male tubulin for NKCC2 blot gel 2 with tubulin control below.

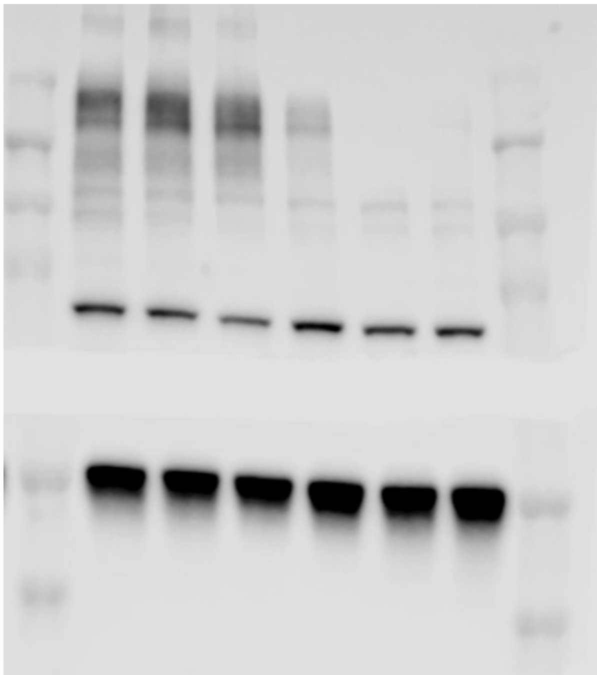

13 week male tubulin for NKCC2 blot gel 2 with tubulin control below.

NKCC2 Female blots

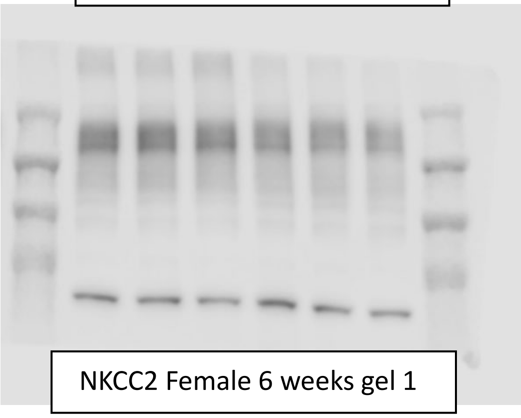

NKCC2 Female 6 weeks gel 1

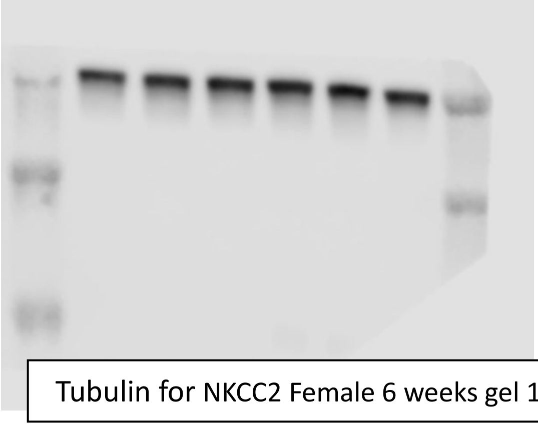

Tubulin for NKCC2 Female 6 weeks gel 1

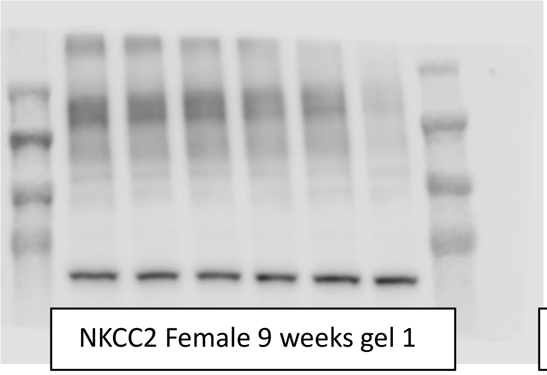

NKCC2 Female 9 weeks gel 1

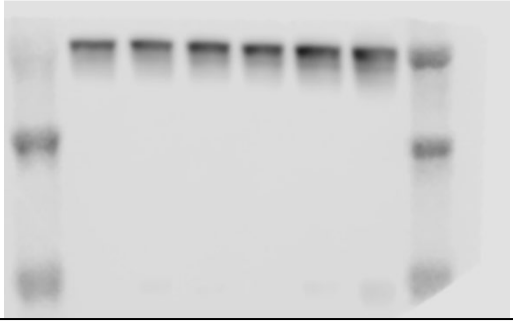

Tubulin for NKCC2 Female 9 weeks gel 1

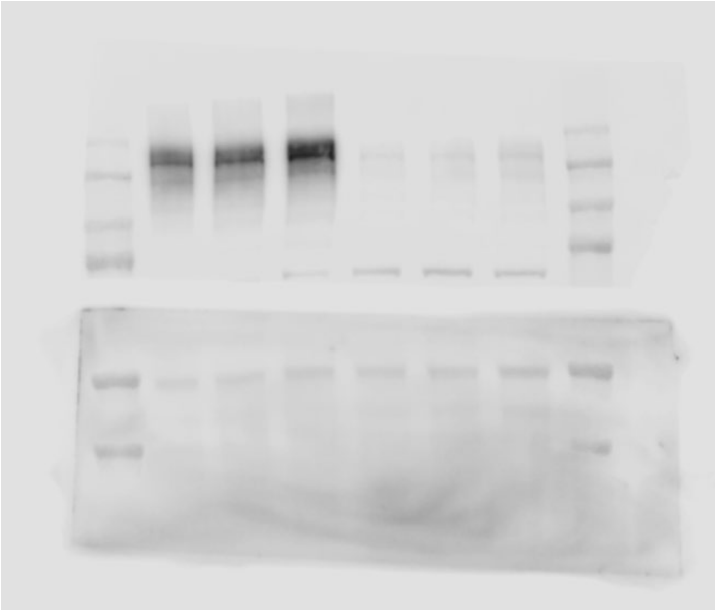

NKCC2 Female 13 weeks gel 1
with tubulin loading control

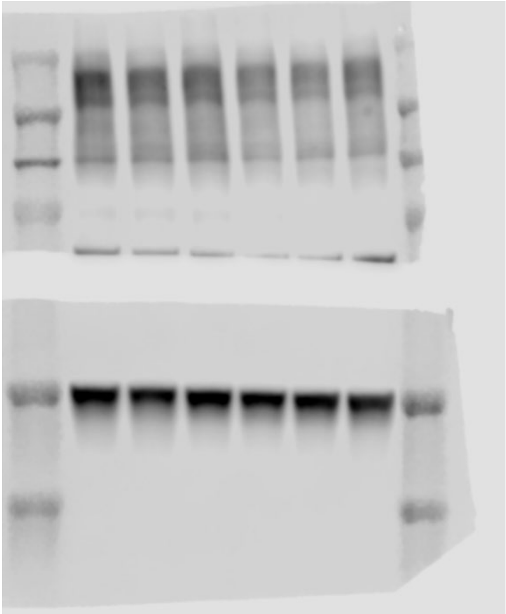

NKCC2 Female 6 weeks gel 2 with tubulin loading control

NKCC2 Female 9 weeks gel 2 with tubulin loading control

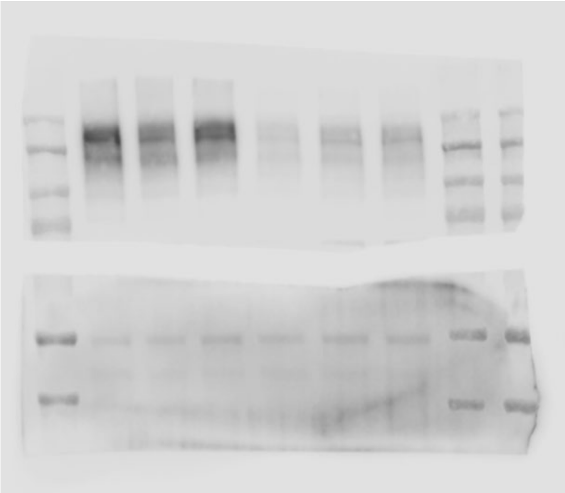

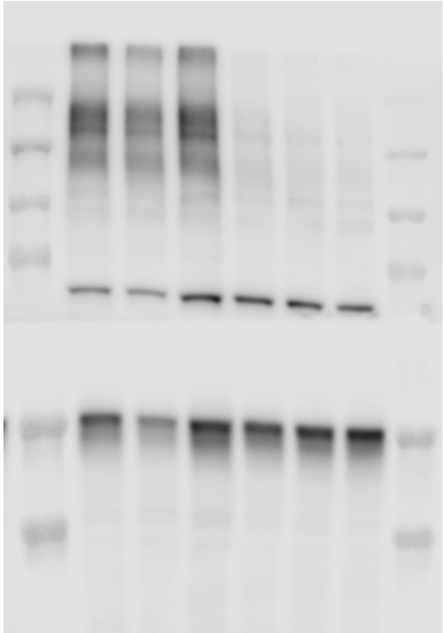

NKCC2 Female 13 weeks gel 2 with tubulin loading control

NCC blots

9 week male and female NCC blots gel 1

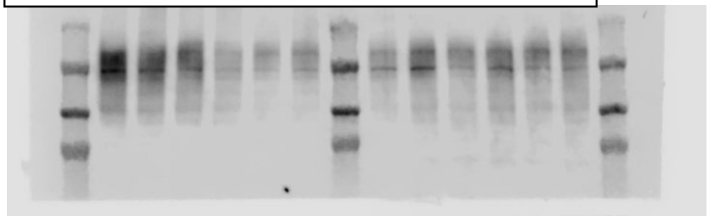

Tubulin 9 week male and female NCC blots gel 1

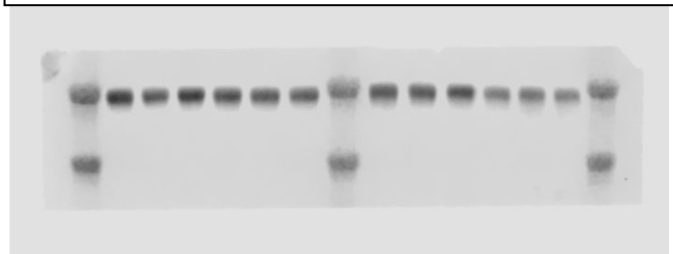

9 week male NCC blots gel 2

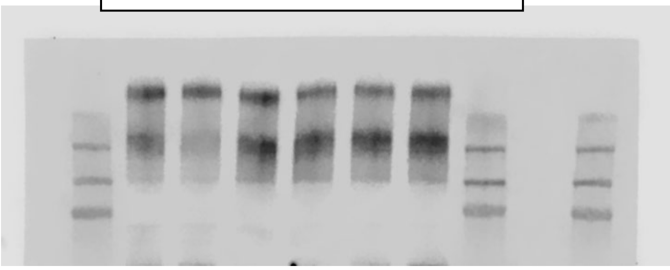

Tubulin 9 week male NCC blots gel 2

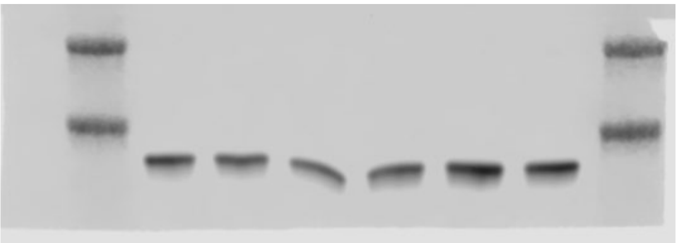

9 week female NCC blots gel 2

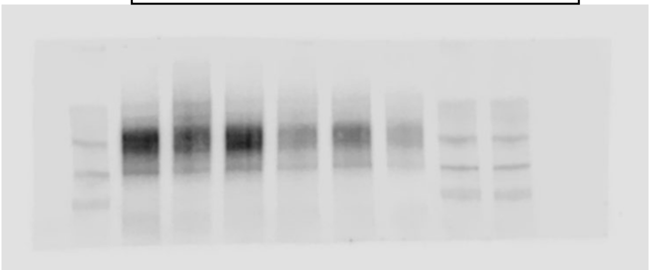

Tubulin 9 week female NCC blots gel 2

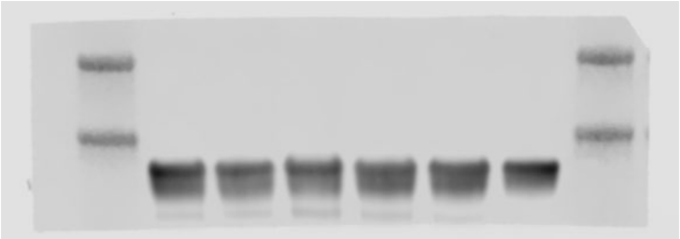

NCC blots

13 weeks NCC Male Blot with Tubulin

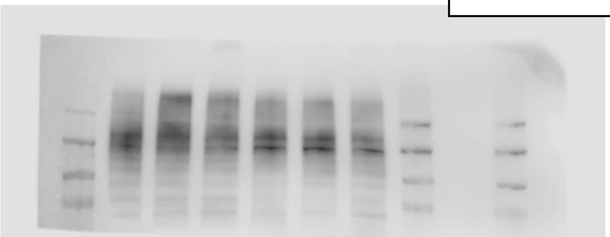

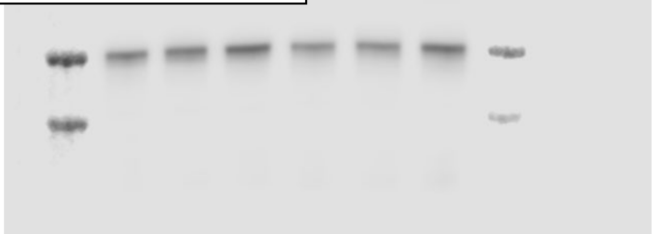

13 weeks NCC Female Blot with Tubulin

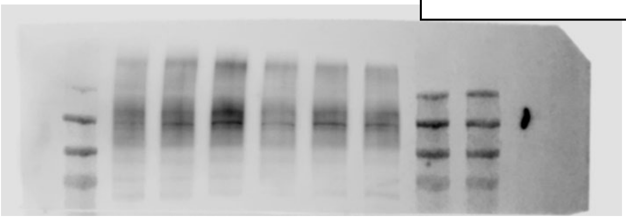

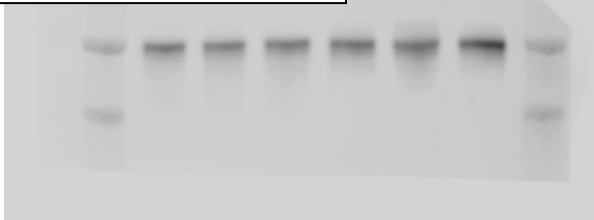

pNCC Blots

9 weeks Male pNCC blot with Gapdh gel 1

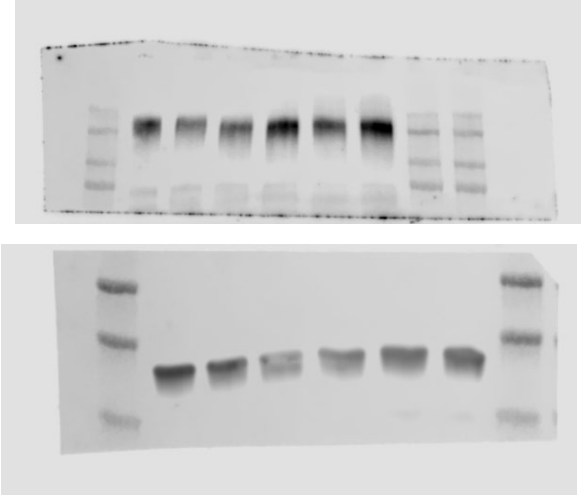

9 week Female pNCC blot with gapdh gel 1

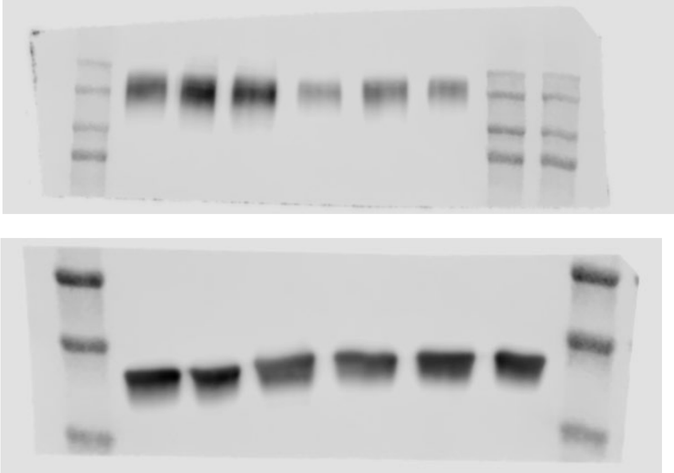

9 weeks Male pNCC blot with Gapdh gel 2

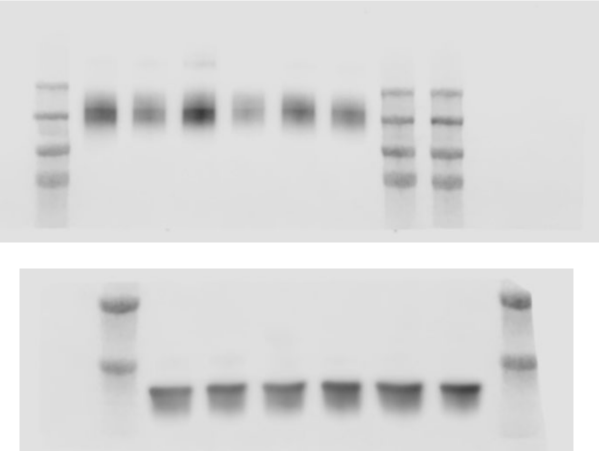

9 weeks Female pNCC blot with Gapdh gel 2

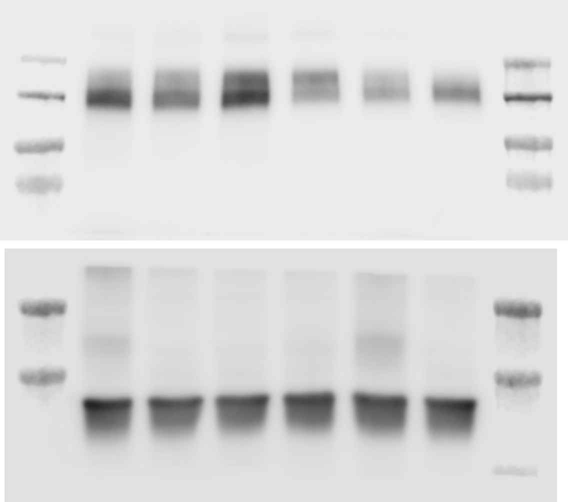

13 weeks Male pNCC blot with Gapdh

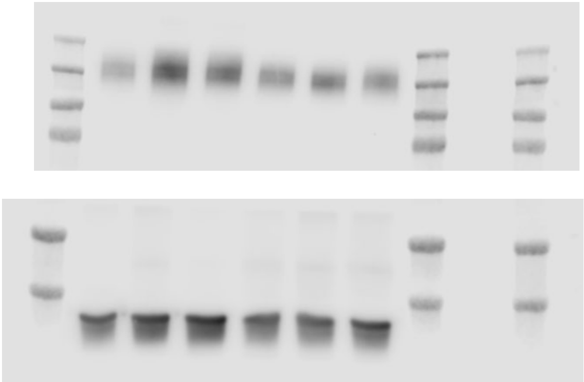

13 weeks Female pNCC blot with Gapdh

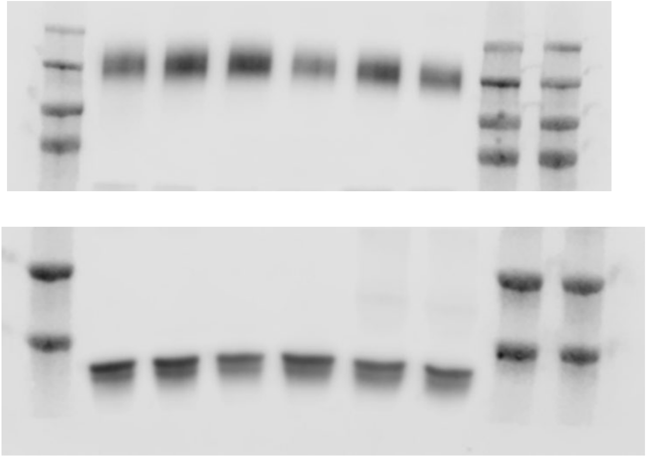

ENAC blots

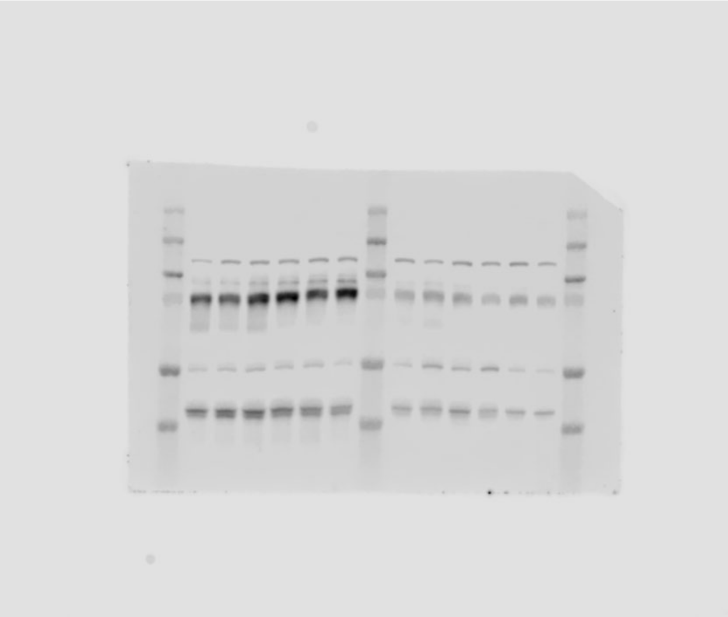

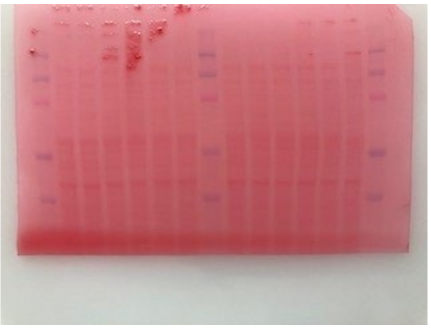

9 week female and male ENAC and ponceau loading control

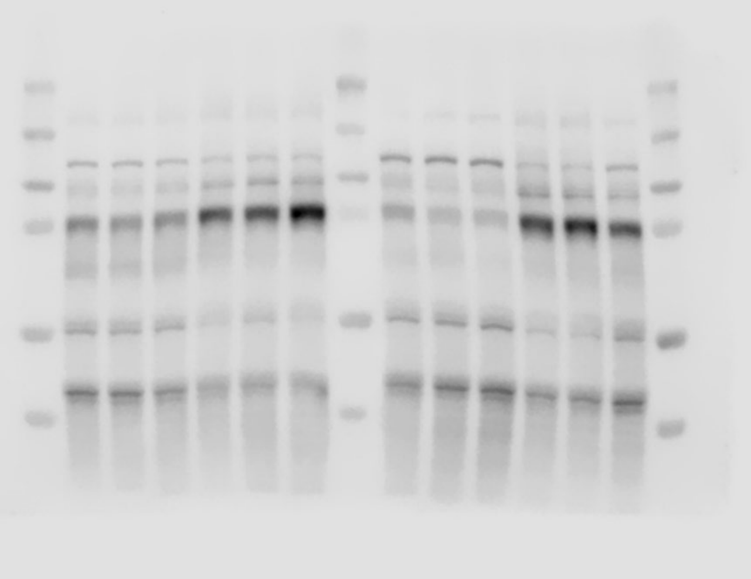

13 week female and male ENAC blot gel 1

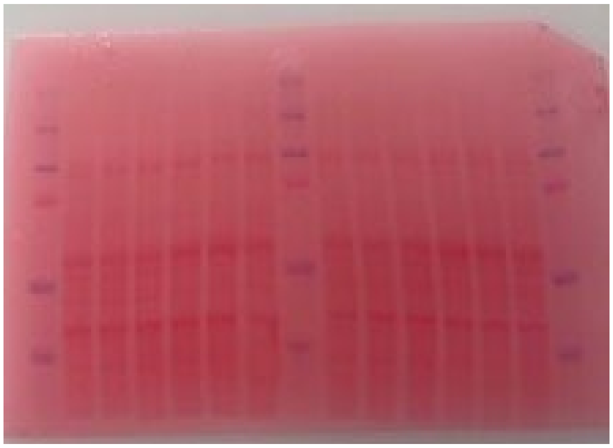

13 week female and male ENAC blot gel 2

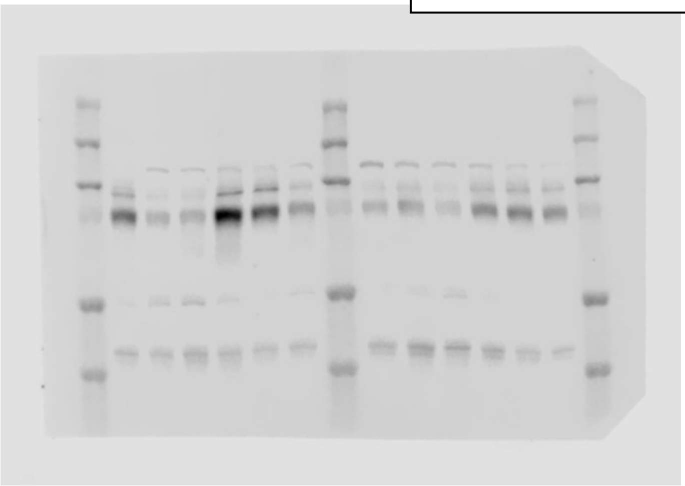

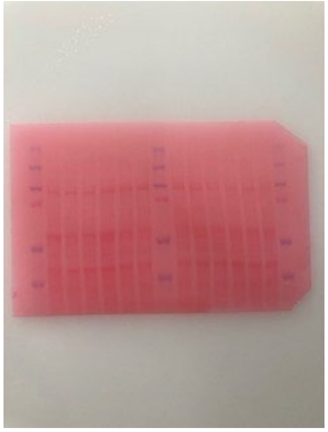

UMOD CRE Only blots

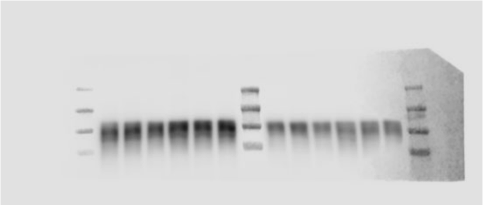

Fig. 3K 6 weeks

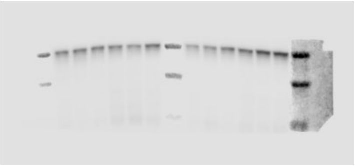

Fig. 3K 12 weeks

Membrane cut site

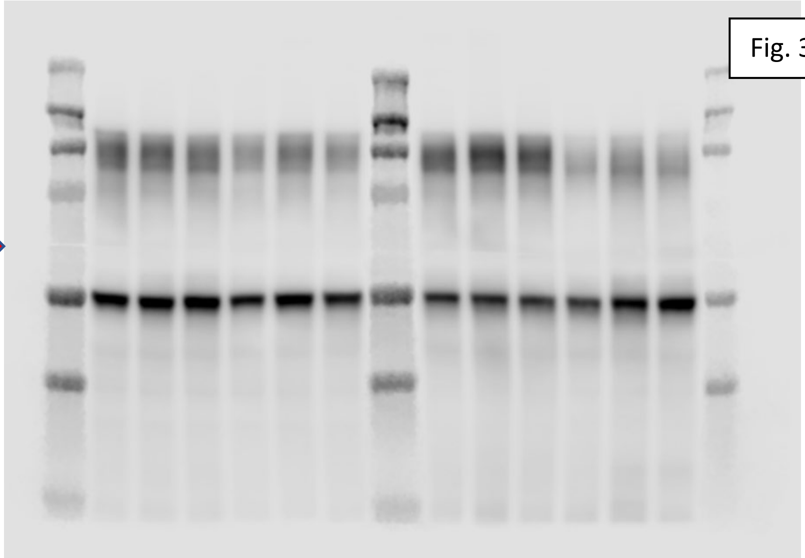

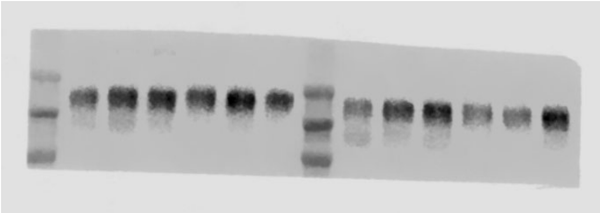

Fig. 5O NKCC2 blot 6 weeks

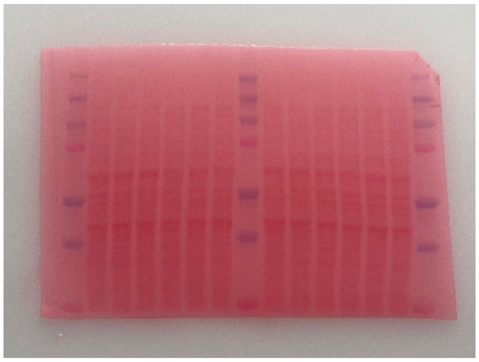

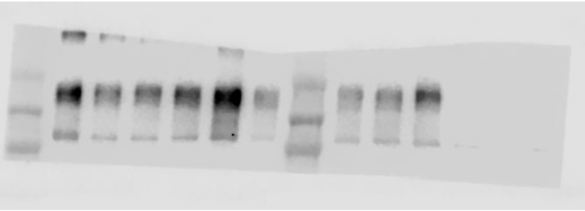

Fig. 5O NKCC2 blot 12 weeks

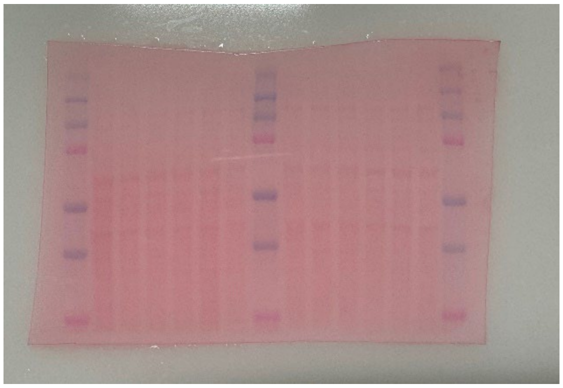

Fig S9B blot 6 weeks

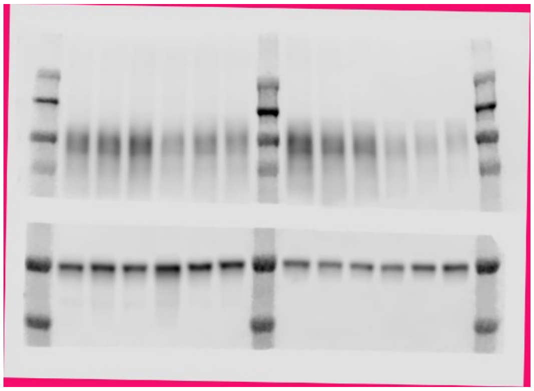

ROMK blot

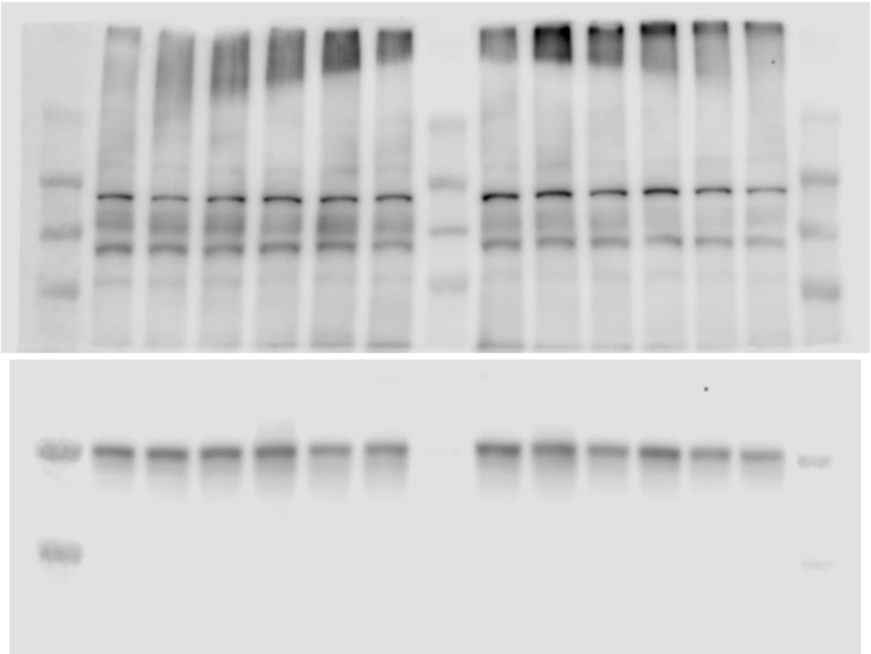

ROMK Female and Male

Tubulin Female and Male

ATF6 blots gel 1 Fig. 4

ATF6 band enclosed in red rectangle

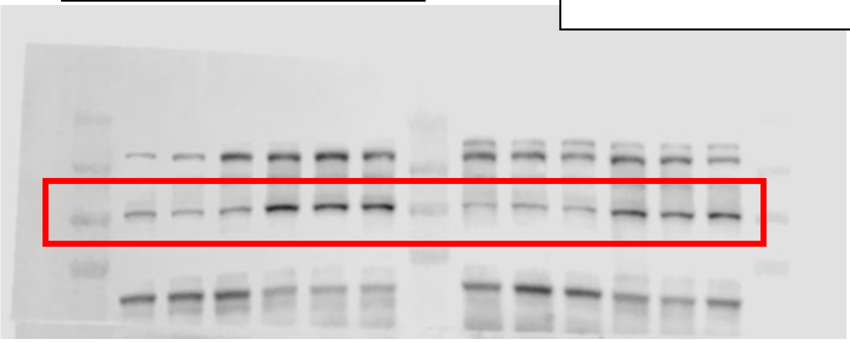

13 week ATF6 and Tubulin loading control.

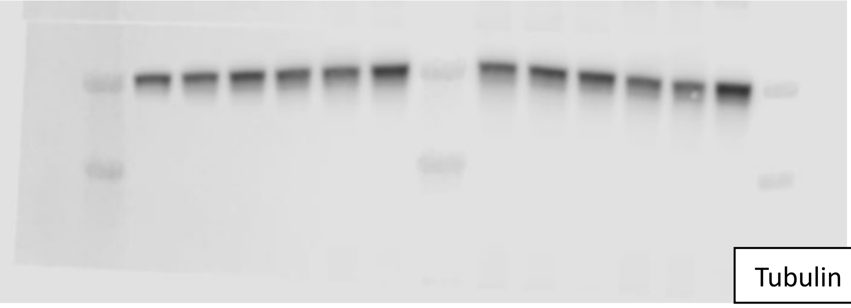

Tubulin

ATF6 blots gel 2 Fig. 4

ATF6 band enclosed in red rectangle

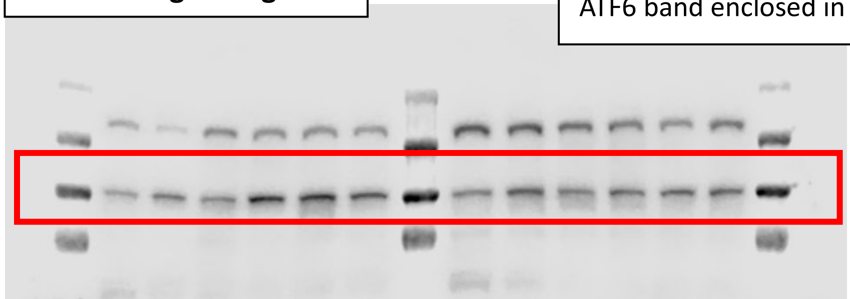

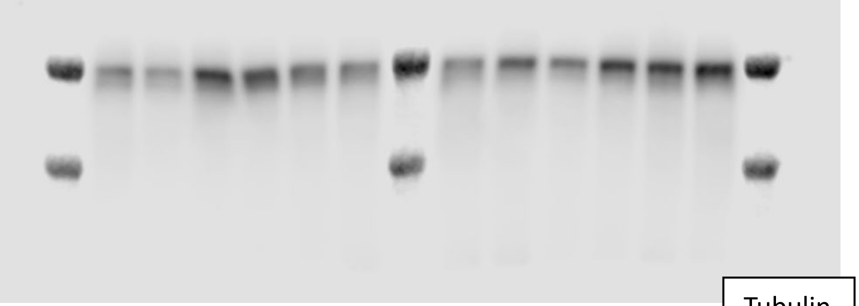

Tubulin

Xbp1 blots gel 1

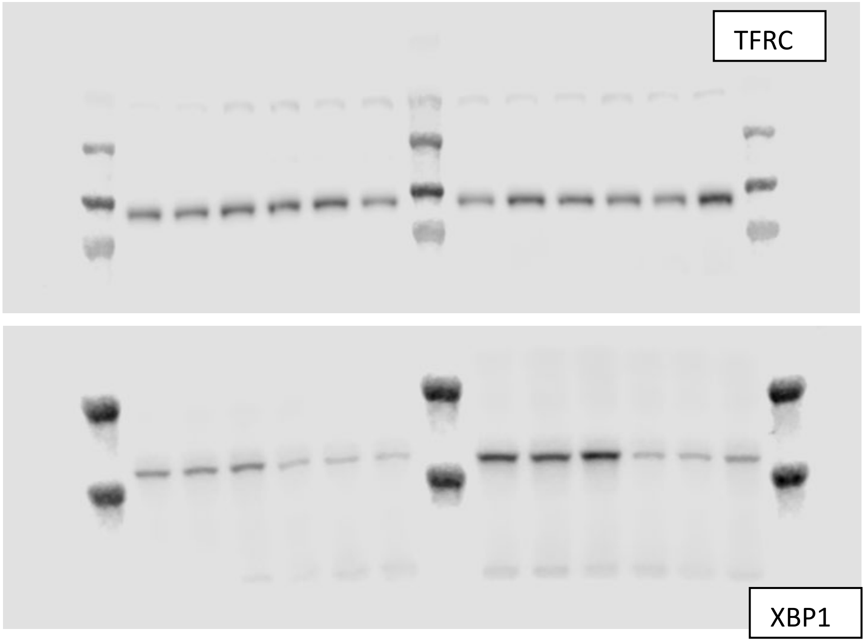

13 week XBP1 and TFRC loading control.

Xbp1 blots gel 2

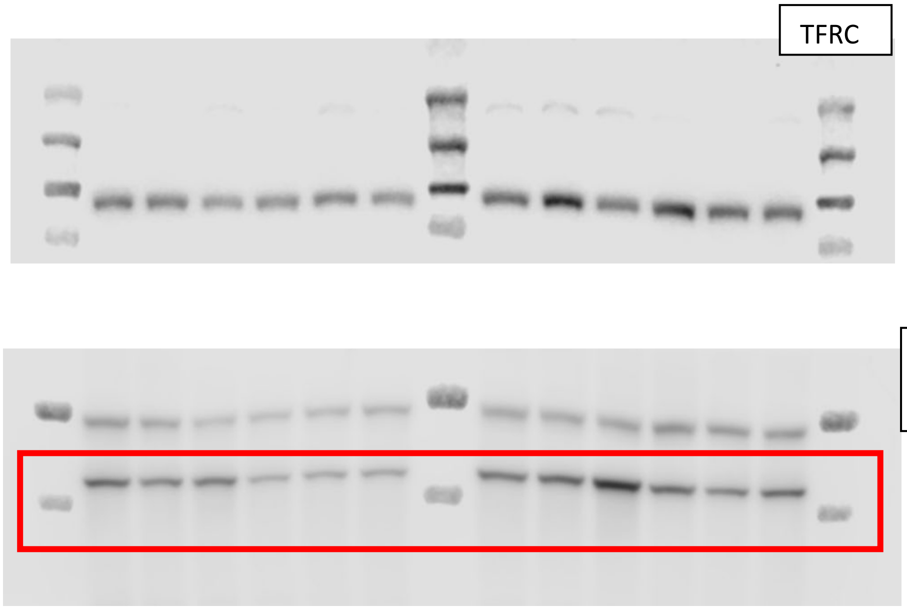

XBP1 bands within red rectangle

CALR blots Female 13 weeks Fig. 4

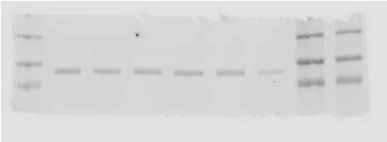

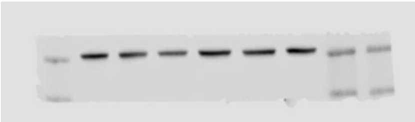

TFRC loading control and calreticulin

CALR blots Male 13 weeks Fig. 4

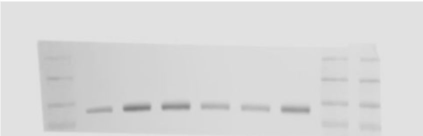

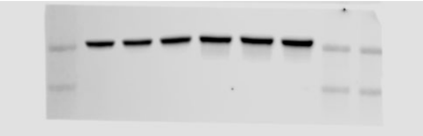

TFRC loading control and calreticulin

CANX blots Fig. 4

13 weeks Male CANX and GAPDH blots

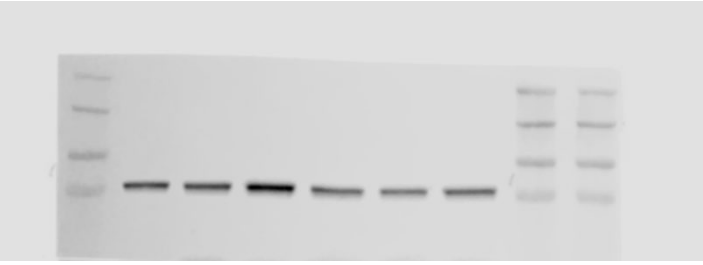

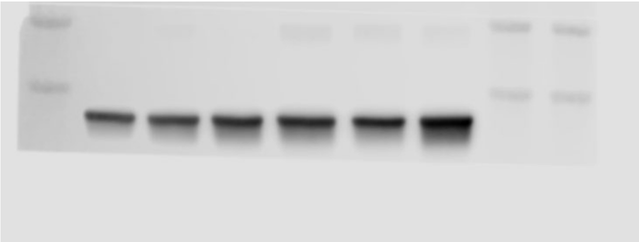

13weeks Female CANX and GAPDH blots

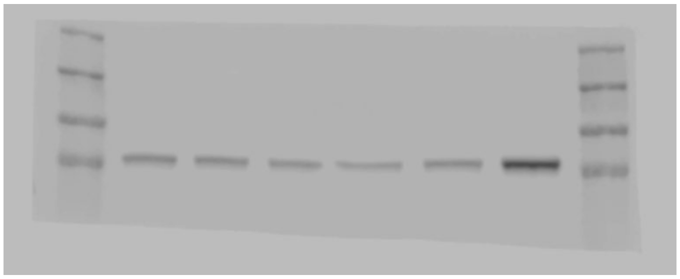

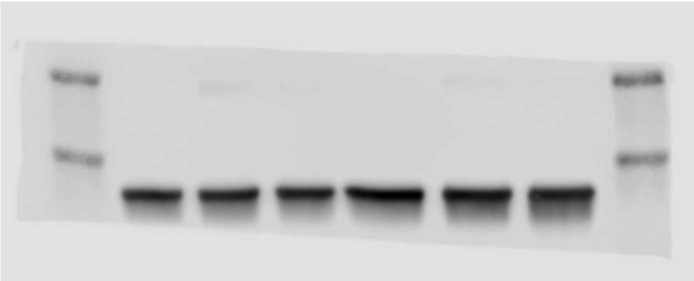

ENaC blots in Fig. S12

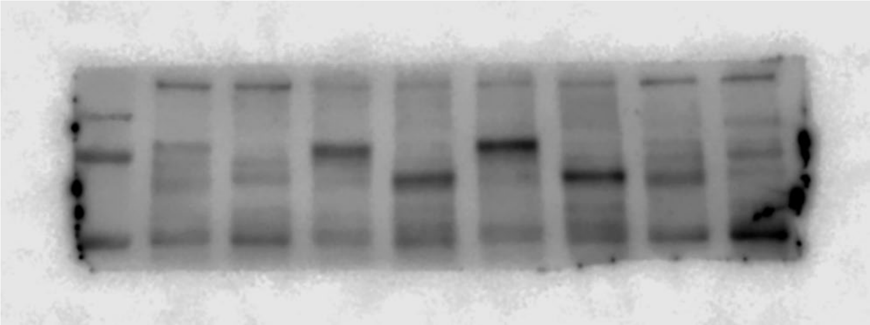

13 week male ENaC with and without PNGase F

Ponceau for 13 week male ENaC blot

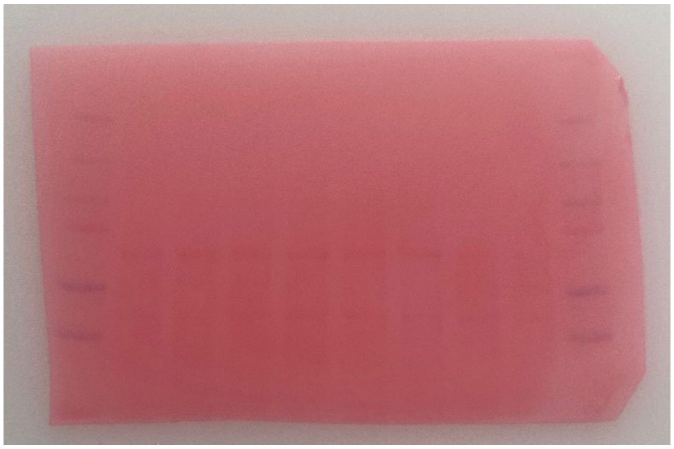

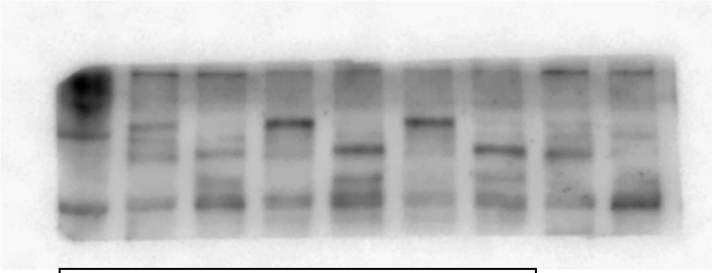

13 weeks female ENaC blot with and without PNGaseF treatment

Ponceau for 13 weeks female ENaC blot

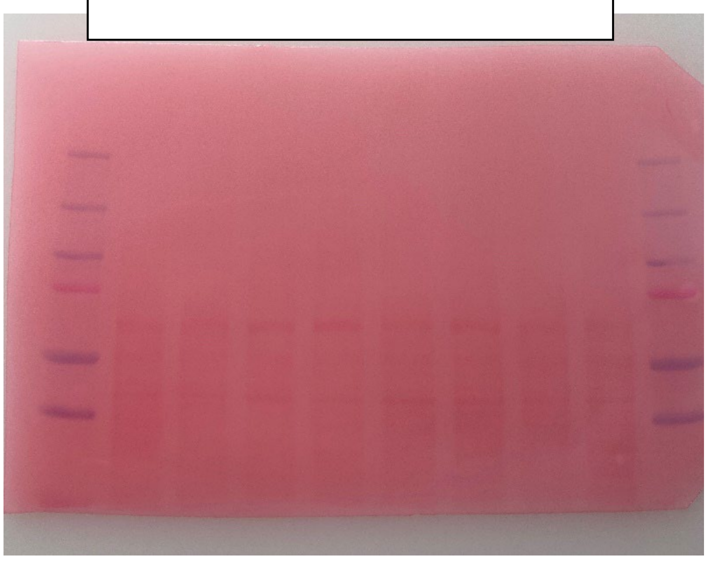

Supplement: zqae048_Supplemental_Files [file zqae048_supplemental_files.zip › Uncut western blot images Final.pdf]
